# Supplementary material for: H3K4 methylation by SETD1A/BOD1L facilitates RIF1-dependent NHEJ
Source: Mol Cell. 2022 May 19;82(10):1924–1939.e10. doi: 10.1016/j.molcel.2022.03.030 (PMC9616806; doi:10.1016/j.molcel.2022.03.030)
Supplement: Document S1. Figures S1–S7 and Table S1 [file mmc1.pdf]

Molecular Cell, Volume *82*

Supplemental information

H3K4 methylation by SETD1A/BOD1L

facilitates RIF1-dependent NHEJ

Rachel Bayley, Valerie Borel, Rhiannon J. Moss, Ellie Sweatman, Philip Ruis, Alice Ormrod, Amalia Goula, Rachel M.A. Mottram, Tyler Stanage, Graeme Hewitt, Marco Saponaro, Grant S. Stewart, Simon J. Boulton, and Martin R. Higgs

Figure S1:

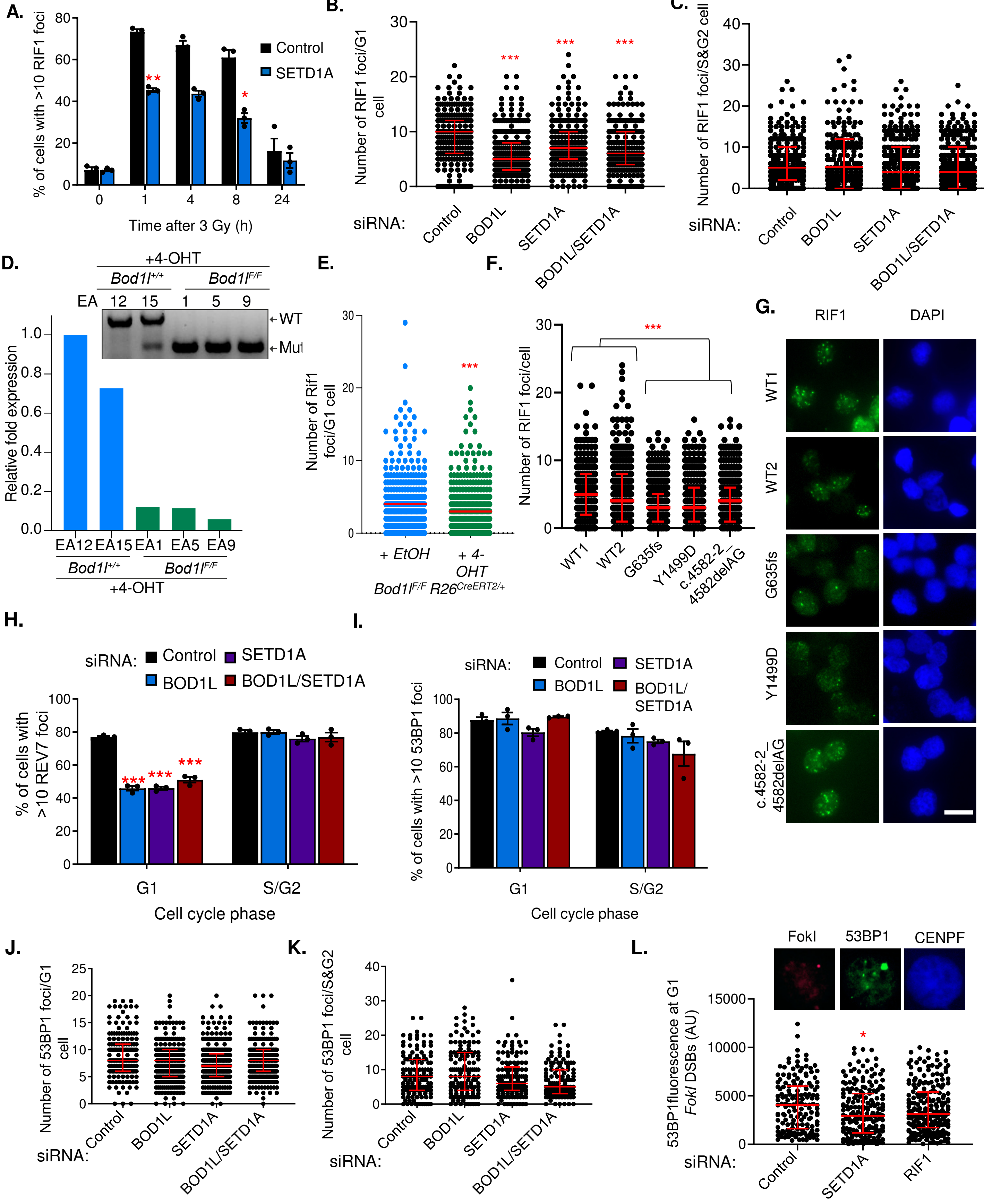
A.
% of cells with >10 RIF1 foci
Control
SETD1A
Time after 3 Gy (h)
B.
Number of RIF1 foci/G1 cell
siRNA:
Control
BOD1L
SETD1A
BOD1L/SETD1A
C.
Number of RIF1 foci/S&G2 cell
D.
+4-OHT
Bod1l+/+
Bod1lF/F
EA 12 15 1 5 9
WT
Mut
Relative fold expression
EA12 EA15 EA1 EA5 EA9
E.
Number of Rif1 foci/G1 cell
+ EtOH
+ 4-OHT
Bod1lF/F R26CreERT2/+
F.
Number of RIF1 foci/cell
WT1
WT2
G635fs
Y1499D
c.4582-2_4582delAG
G.
RIF1
DAPI
H.
siRNA:
Control
SETD1A
BOD1L
BOD1L/SETD1A
% of cells with >10 REV7 foci
G1
S/G2
Cell cycle phase
I.
% of cells with >10 53BP1 foci
J.
Number of 53BP1 foci/G1 cell
K.
Number of 53BP1 foci/S&G2 cell
L.
FokI
53BP1
CENPF
53BP1fluorescence at G1 FokI DSBs (AU)
RIF1

Figure S1, related to Figure 2: SETD1A and BOD1L are required for recruitment of RIF1, but not 53BP1, to DNA double-strand breaks. (A) HeLa cells were transfected with the indicated siRNA, exposed to ionizing radiation (IR), and RIF1 foci formation enumerated by immunofluorescence microscopy at the indicated times. **(B-C)** RIF1 foci formation was enumerated in CENPF-negative (B) or positive (C) cells from (A) at 8 h post IR. Lines denote mean values from three independent experiments. **(D)** *Bod1l*$^{F/F}$ or *Bod1l*$^{+/+}$ MEFs were treated with 4-OHT, and the expression of mutant *Bod1l* alleles (above) and relative quantification of *Bod1l* gene expression by qPCR (below) was assessed. **(E)** MEFs from (D) were irradiated, and immunostained with antibodies to CENPF and RIF1. Foci formation was enumerated by fluorescence microscopy, and the number of foci per G1 cell is indicated. Lines denote mean values from three independent experiments. **(F-G)** RIF1 foci formation and representative images of RIF1 foci in cells bearing heterozygous SETD1A mutations after exposure to IR (see Figure 2E). Scale bars = 10 µm. **(H)** HeLa cells were transfected with the indicated siRNA, irradiated, and immunostained with antibodies to CENPF and REV7. Foci formation was enumerated by fluorescence microscopy, and the number of cells with >10 foci per cell is denoted. **(I-K)** HeLa cells were transfected with the indicated siRNA, irradiated, and immunostained with antibodies to CENPF and 53BP1. Foci formation was enumerated by fluorescence microscopy, and the number of cells with >10 foci per cell (I), as well as the number of foci per cell (J-K) is indicated. Lines denote mean values from three independent experiments. **(L)** U-2-OS-FokI cells were transfected with the indicated siRNA, treated with 4-OHT and immunostained with antibodies to CENPF and 53BP1. Representative images are shown above, and fluorescence intensity per FokI-positive focus was quantified using ImageJ. Lines denote mean values from three independent experiments. Plots in all cases represent mean data from three independent experiments. Error bars = mean ± SEM, *P* values: unpaired two-tailed t-tests (A, H) or Mann-Whitney (B, C, E, F, J-L). *p≤0.05, **p ≤ 0.01 and ***p ≤ 0.001.

Figure S2:

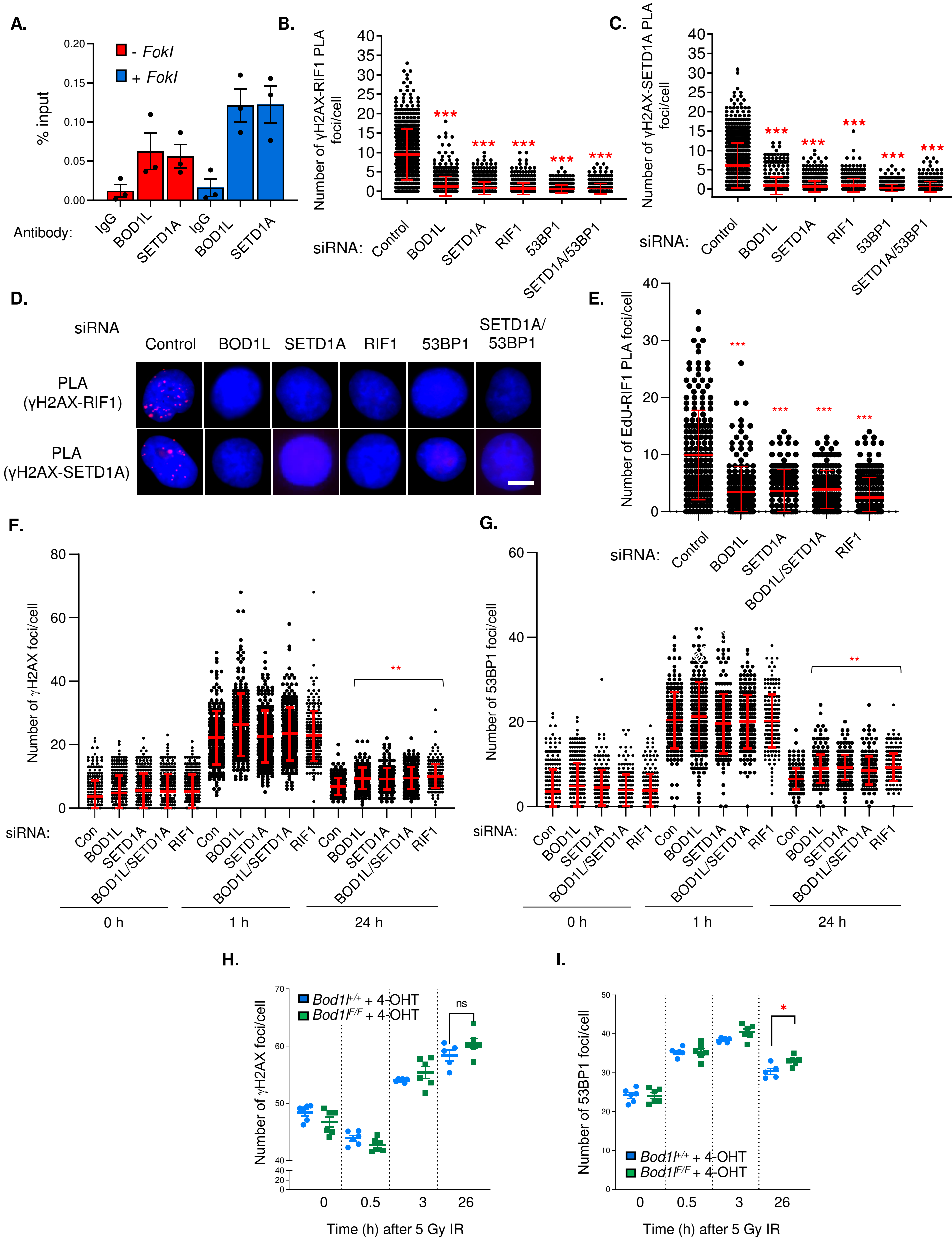
A.
- FokI
+ FokI
% input
0.20
0.15
0.10
0.05
0.00
Antibody:
IgG
BOD1L
SETD1A
IgG
BOD1L
SETD1A
B.
Number of γH2AX-RIF1 PLA foci/cell
siRNA:
Control
BOD1L
SETD1A
RIF1
53BP1
SETD1A/53BP1
***
C.
Number of γH2AX-SETD1A PLA foci/cell
siRNA:
Control
BOD1L
SETD1A
RIF1
53BP1
SETD1A/53BP1
***
D.
siRNA
Control
BOD1L
SETD1A
RIF1
53BP1
SETD1A/ 53BP1
PLA (γH2AX-RIF1)
PLA (γH2AX-SETD1A)
E.
Number of EdU-RIF1 PLA foci/cell
siRNA:
Control
BOD1L
SETD1A
BOD1L/SETD1A
RIF1
***
F.
Number of γH2AX foci/cell
siRNA:
Con
BOD1L
SETD1A
BOD1L/SETD1A
RIF1
0 h
1 h
24 h
**
G.
Number of 53BP1 foci/cell
siRNA:
Con
BOD1L
SETD1A
BOD1L/SETD1A
RIF1
0 h
1 h
24 h
**
H.
Bod1l+/+ + 4-OHT
Bod1lF/F + 4-OHT
ns
Number of γH2AX foci/cell
0
0.5
3
26
Time (h) after 5 Gy IR
I.
*
Number of 53BP1 foci/cell
Bod1l+/+ + 4-OHT
Bod1lF/F + 4-OHT
0
0.5
3
26
Time (h) after 5 Gy IR

Figure S2, related to Figure 2: SETD1A and BOD1L suppress DSB resection after IR. (A) U-2-OS-FokI cells were treated with 4-OHT, chromatin was isolated and immunoprecipitated with the indicated antibodies and quantified by qPCR. **(B-D)** Quantification of PLA signals between γH2AX and RIF1 (B), or between γH2AX and SETD1A (C), in HeLa cells transfected with the indicated siRNAs and exposed to IR. Representative images are shown in (D). **(E)** Quantification of PLA signals between EdU and RIF1 in HeLa cells transfected with the indicated siRNAs and exposed to 5 mM hydroxyurea for 4 hours. **(F-G)** Enumeration of gH2AX (F) and 53BP1 (G) foci in HeLa cells from Figure 2G. Lines denote mean values from three independent experiments. **(H-I)** Enumeration of γH2AX (H) and 53BP1 (I) foci in MEFs from Figure 2J. Plots in all cases represent mean data from three independent experiments. Error bars = mean ± SEM, *P* values: One-way ANOVA (H, I); Mann-Whitney (B, C, E, F, G). $^{*}p \leq 0.05$, $^{**}p \leq 0.01$ and $^{***}p \leq 0.001$.

Figure S3:

A.

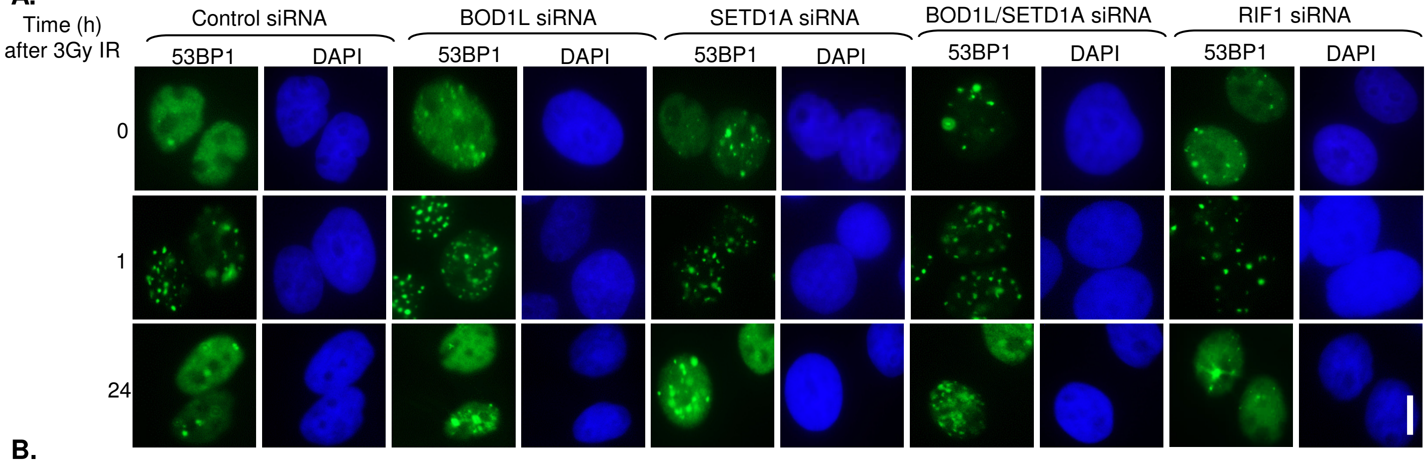

B.

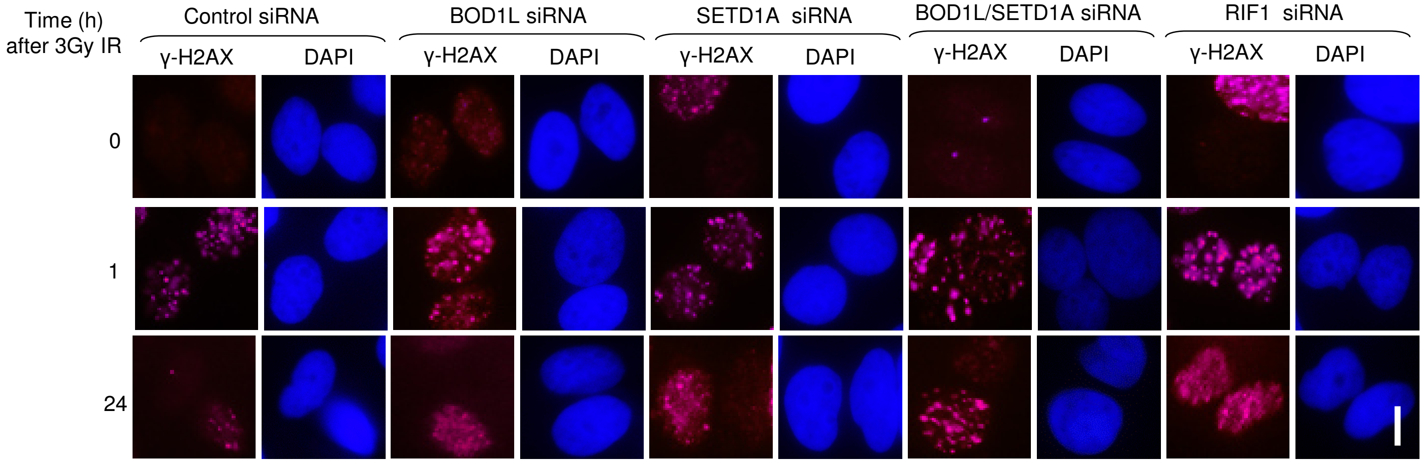

C.

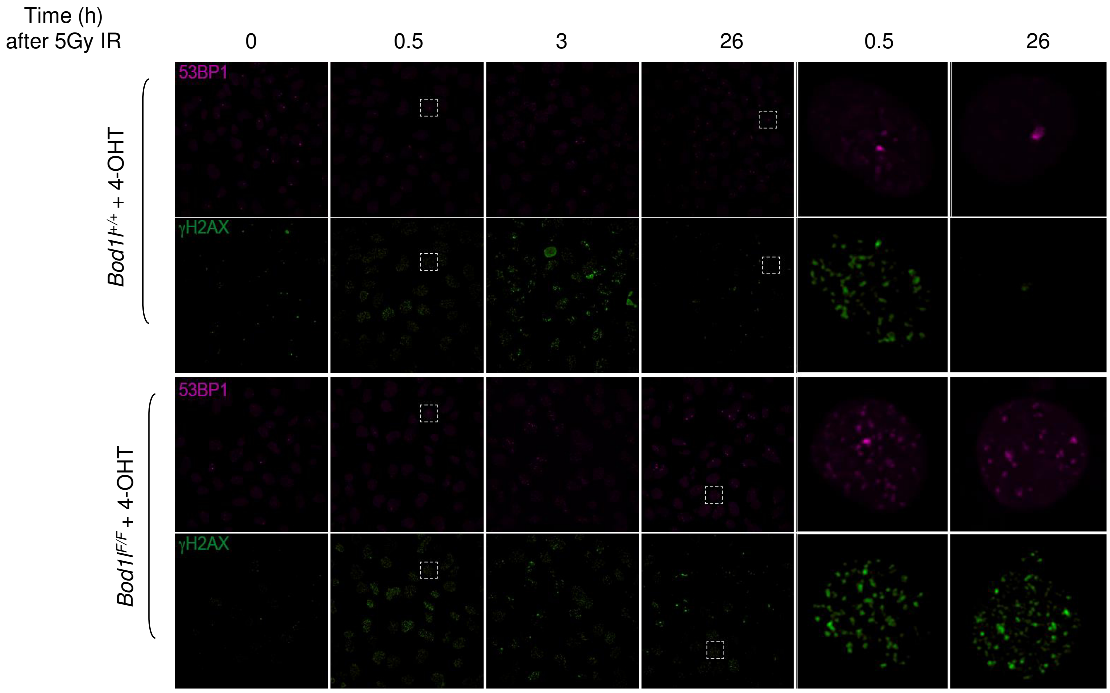

Figure S3, related to Figure 2: Loss of SETD1A and BOD1L compromises efficient DSB repair (A-C) Representative images of γH2AX or 53BP1 foci formation in irradiated HeLa cells or MEFs from Figure 2G-K. Scale bars = 10 µm.

Figure S4.

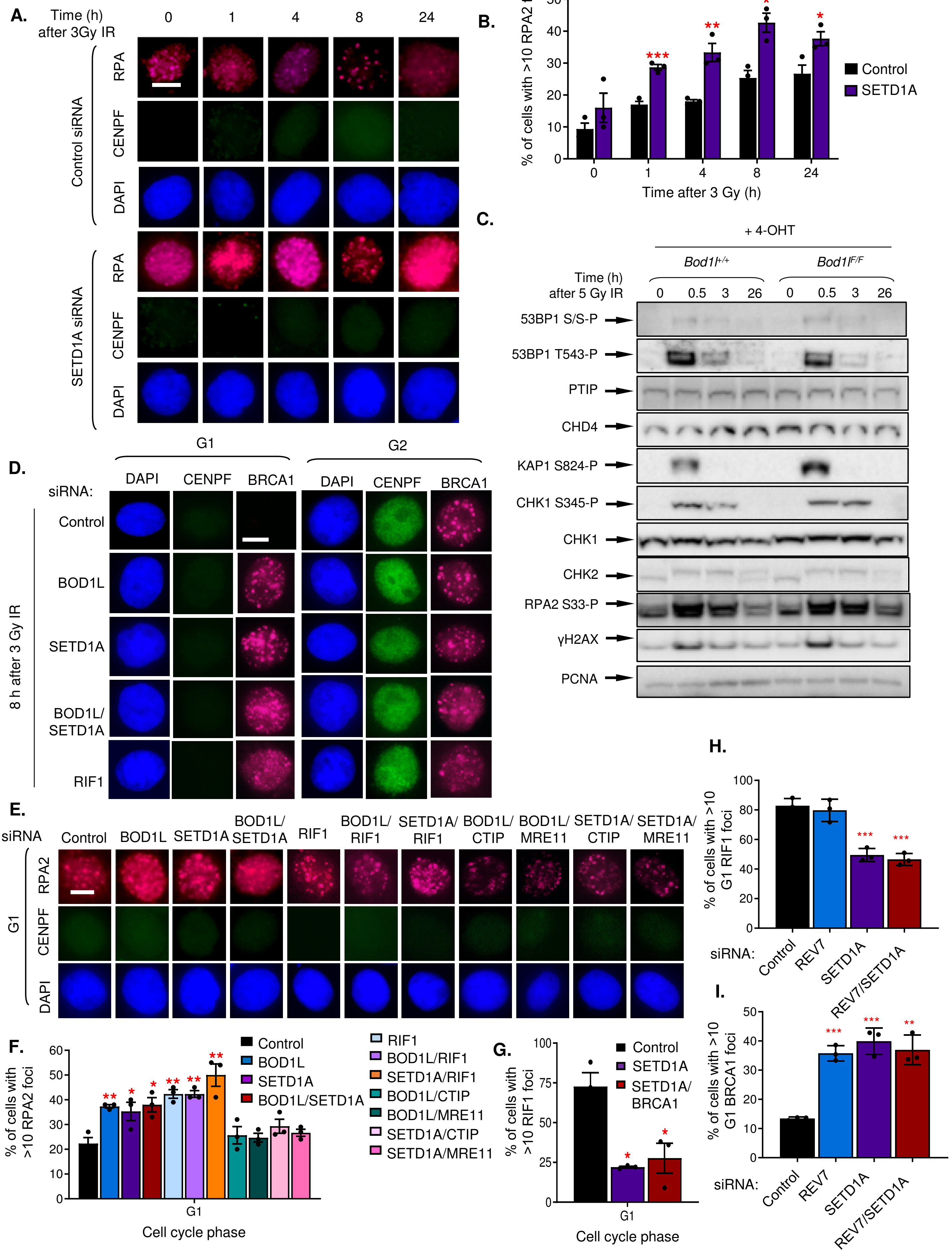

A.
Time (h) after 3Gy IR
0 1 4 8 24
Control siRNA
SETD1A siRNA
RPA
CENPF
DAPI
B.
% of cells with >10 RPA2 foci
Time after 3 Gy (h)
Control
SETD1A
C.
+ 4-OHT
Bod1l+/+
Bod1lF/F
Time (h) after 5 Gy IR
0 0.5 3 26
53BP1 S/S-P
53BP1 T543-P
PTIP
CHD4
KAP1 S824-P
CHK1 S345-P
CHK1
CHK2
RPA2 S33-P
γH2AX
PCNA
D.
G1
G2
siRNA:
DAPI
CENPF
BRCA1
Control
BOD1L
SETD1A
BOD1L/ SETD1A
RIF1
8 h after 3 Gy IR
E.
siRNA
Control
BOD1L
SETD1A
BOD1L/ SETD1A
RIF1
BOD1L/ RIF1
SETD1A/ RIF1
BOD1L/ CTIP
BOD1L/ MRE11
SETD1A/ CTIP
SETD1A/ MRE11
G1
RPA2
CENPF
DAPI
F.
% of cells with >10 RPA2 foci
Control
BOD1L
SETD1A
BOD1L/SETD1A
RIF1
BOD1L/RIF1
SETD1A/RIF1
BOD1L/CTIP
BOD1L/MRE11
SETD1A/CTIP
SETD1A/MRE11
G1
Cell cycle phase
G.
% of cells with >10 RIF1 foci
Control
SETD1A
SETD1A/ BRCA1
G1
Cell cycle phase
H.
% of cells with >10 G1 RIF1 foci
siRNA:
Control
REV7
SETD1A
REV7/SETD1A
I.
% of cells with >10 G1 BRCA1 foci
siRNA:
Control
REV7
SETD1A
REV7/SETD1A

Figure S4, related to Figure 3: Loss of BOD1L and SETD1A increases BRCA1-, CtIP- and MRE11-dependent end-resection of DNA double-strand breaks. (A-B) HeLa cells were transfected with the denoted siRNAs, irradiated, and immunostained with antibodies against RPA2 and CENPF. Representative images (A) and percentage of cells with >10 RPA2 foci (B) enumerated by immunofluorescence microscopy are shown. Scale bars = 10 µm. **(C)** *Bod1l*$^{F/F}$ or *Bod1l*$^{+/+}$ MEFs were treated with 4-OHT, exposed to ionizing radiation, and whole cell extracts from the indicated time points immunoblotted with the indicated antibodies. **(D)** Representative images of ionizing radiation-induced BRCA1 foci in HeLa cells from Figure 3J following BOD1L, SETD1A or RIF1 depletion. **(E-F)** HeLa cells were transfected with the denoted siRNAs, irradiated, and immunostained with antibodies against RPA2 and CENPF. Representative images (E) and percentage of cells with >10 RPA2 foci (F) enumerated by immunofluorescence microscopy are shown. **(G-I)** HeLa cells were transfected with the denoted siRNAs, irradiated, immunostained with antibodies against either RIF1 and CENPF (G-H) or BRCA1 and CENPF (I), and foci enumerated by immunofluorescence microscopy. Plots in all cases represent mean data from three independent experiments. Error bars = mean ± SEM, *P* values: unpaired two-tailed t-test (A, F, G, H, I). *p≤0.05, **p ≤ 0.01 and ***p ≤ 0.001.

Figure S5.

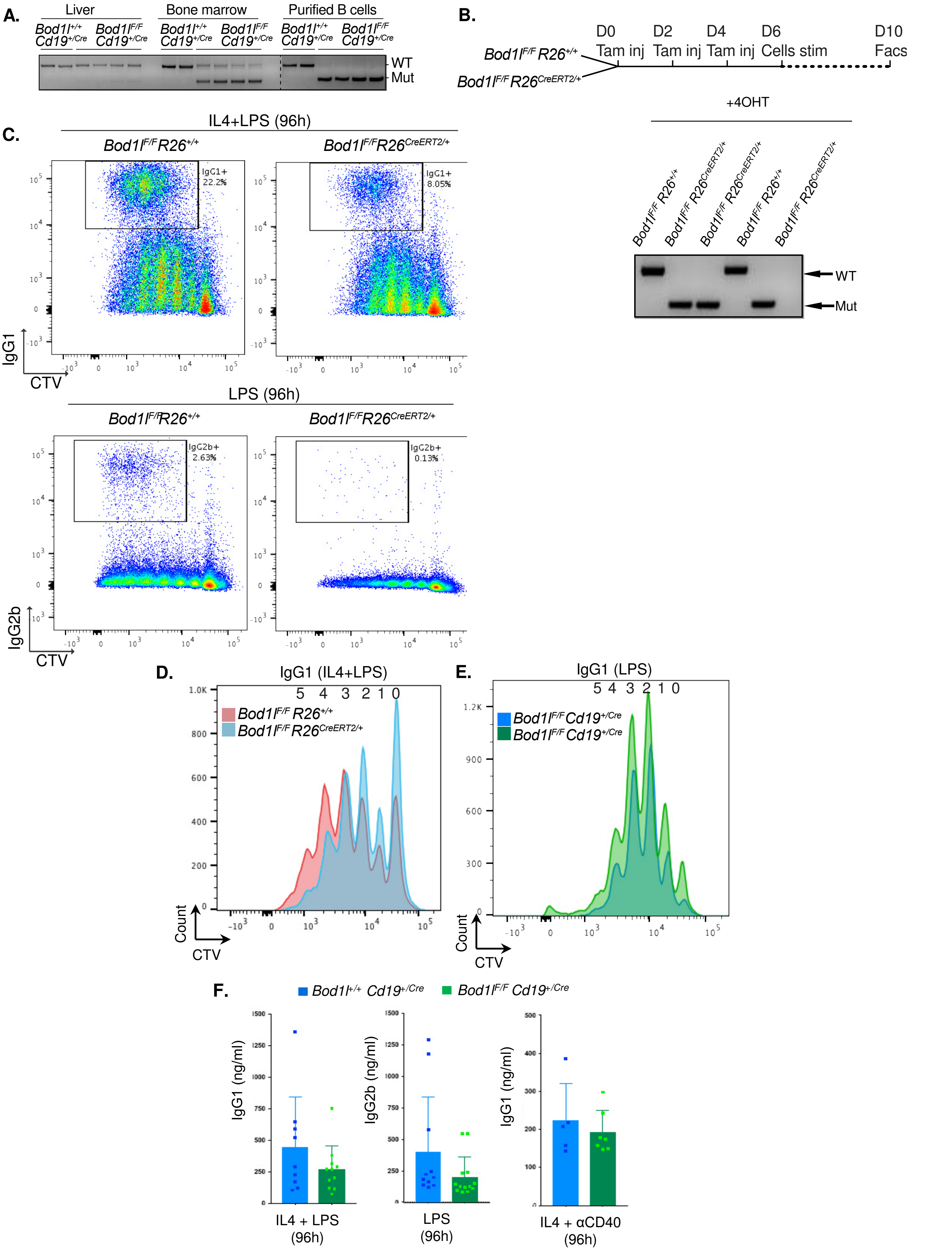

A.
Liver
Bone marrow
Purified B cells
Bod1l+/+ Cd19+/Cre
Bod1lF/F Cd19+/Cre
WT
Mut
B.
D0 Tam inj
D2 Tam inj
D4 Tam inj
D6 Cells stim
D10 Facs
Bod1lF/F R26+/+
Bod1lF/F R26CreERT2/+
+4OHT
Bod1lF/F R26+/+
Bod1lF/F R26CreERT2/+
Bod1lF/F R26CreERT2/+
Bod1lF/F R26+/+
Bod1lF/F R26CreERT2/+
WT
Mut
C.
IL4+LPS (96h)
Bod1lF/F R26+/+
Bod1lF/F R26CreERT2/+
IgG1+ 22.2%
IgG1+ 8.05%
IgG1
CTV
LPS (96h)
Bod1lF/F R26+/+
Bod1lF/F R26CreERT2/+
IgG2b+ 2.63%
IgG2b+ 0.13%
IgG2b
CTV
D.
IgG1 (IL4+LPS)
5 4 3 2 1 0
Bod1lF/F R26+/+
Bod1lF/F R26CreERT2/+
Count
CTV
E.
IgG1 (LPS)
5 4 3 2 1 0
Bod1lF/F Cd19+/Cre
Bod1lF/F Cd19+/Cre
Count
CTV
F.
Bod1l+/+ Cd19+/Cre
Bod1lF/F Cd19+/Cre
IgG1 (ng/ml)
IL4 + LPS (96h)
IgG2b (ng/ml)
LPS (96h)
IgG1 (ng/ml)
IL4 + αCD40 (96h)

Figure S5, related to Figure 5: Bod1l deletion leads to defective class switch recombination. (A) Cells were isolated from *Bod1l*$^{+/+}$*Cd19*$^{+/Cre}$ or *Bod1l*$^{F/F}$*Cd19*$^{+/Cre}$ mice, and the expression of mutant *Bod1l* alleles was analysed by PCR. **(B)** Schematic of tamoxifen treatments in *Bod1l*$^{F/F}$*R26*$^{+/+}$ or *Bod1l*$^{F/F}$*R26*$^{CreERT2/+}$ mice, and genotyping of *Bod1l* alleles in splenic cells (below). **(C)** Splenic B cells from *Bod1l*$^{F/F}$*R26*$^{+/+}$ or *Bod1l*$^{F/F}$*R26*$^{CreERT2/+}$ mice were stimulated as indicated and stained for surface IgG1 or IgE. **(D-E)** Cell trace violet (CTV) dilutions in purified B cells isolated from the denoted mouse genotype, cultured for 96 h in the presence of LPS and IL-4 and then assessed by FACS. **(F)** CD19+ B cells were isolated from *Bod1l*$^{+/+}$*Cd19*$^{+/Cre}$ or *Bod1l*$^{F/F}$*Cd19*$^{+/Cre}$ mice 96 h after *in vitro* stimulation with the indicated factors, and immunoglobulins quantified by ELISA. Plots in all cases represent mean data from n=3 mice. Error bars = mean ± SEM.

Figure S6:

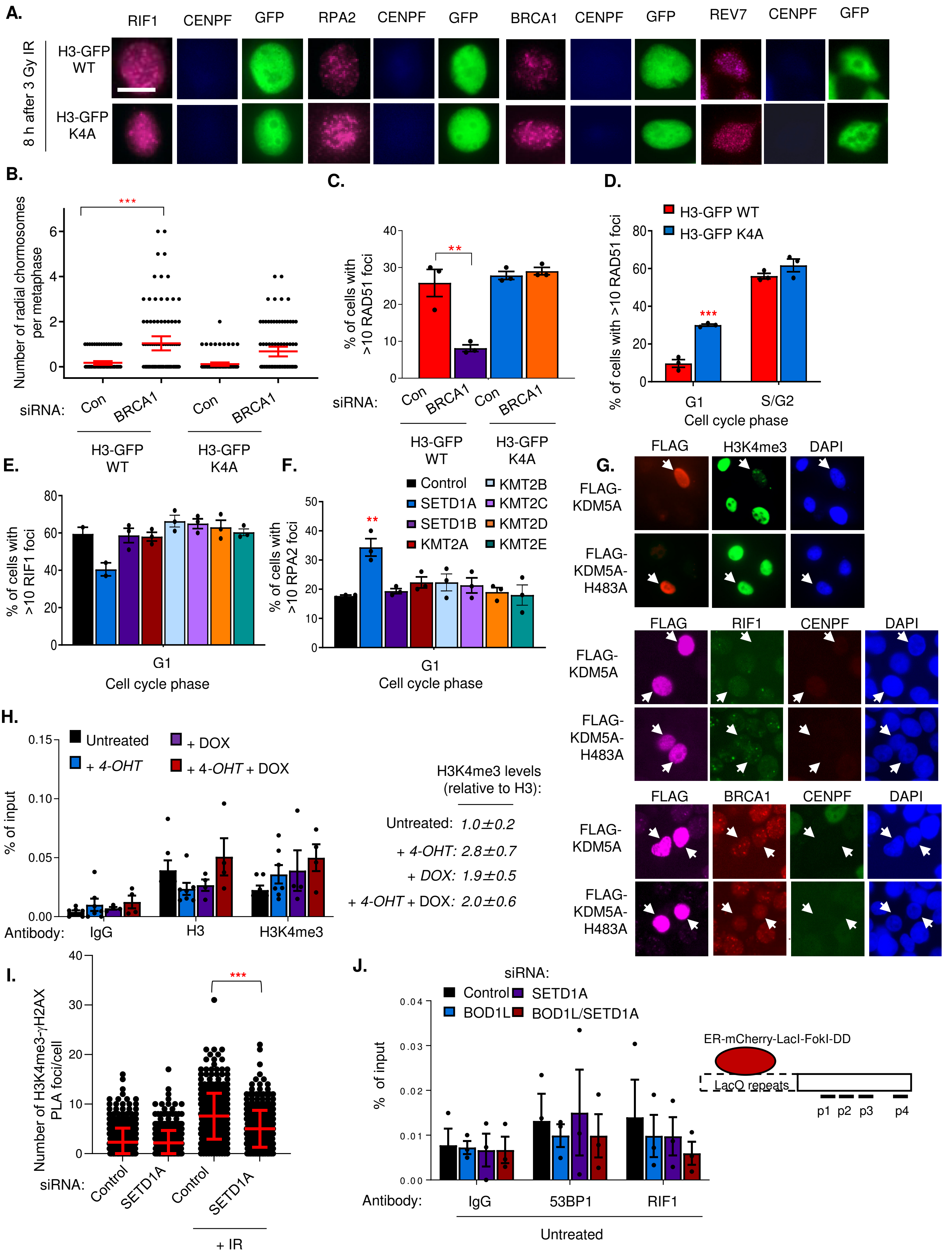
A.
RIF1
CENPF
GFP
RPA2
CENPF
GFP
BRCA1
CENPF
GFP
REV7
CENPF
GFP
8 h after 3 Gy IR
H3-GFP WT
H3-GFP K4A
B.
Number of radial chromosomes per metaphase
***
siRNA:
Con
BRCA1
Con
BRCA1
H3-GFP WT
H3-GFP K4A
C.
% of cells with >10 RAD51 foci
**
siRNA:
Con
BRCA1
Con
BRCA1
H3-GFP WT
H3-GFP K4A
D.
H3-GFP WT
H3-GFP K4A
% of cells with >10 RAD51 foci
***
G1
S/G2
Cell cycle phase
E.
% of cells with >10 RIF1 foci
G1
Cell cycle phase
F.
Control
SETD1A
SETD1B
KMT2A
KMT2B
KMT2C
KMT2D
KMT2E
% of cells with >10 RPA2 foci
**
G1
Cell cycle phase
G.
FLAG
H3K4me3
DAPI
FLAG-KDM5A
FLAG-KDM5A-H483A
FLAG
RIF1
CENPF
DAPI
FLAG-KDM5A
FLAG-KDM5A-H483A
FLAG
BRCA1
CENPF
DAPI
FLAG-KDM5A
FLAG-KDM5A-H483A
H.
Untreated
+ DOX
+ 4-OHT
+ 4-OHT + DOX
% of input
Antibody:
IgG
H3
H3K4me3
H3K4me3 levels (relative to H3):
Untreated: 1.0±0.2
+ 4-OHT: 2.8±0.7
+ DOX: 1.9±0.5
+ 4-OHT + DOX: 2.0±0.6
I.
Number of H3K4me3-γH2AX PLA foci/cell
***
siRNA:
Control
SETD1A
Control
SETD1A
+ IR
J.
siRNA:
Control
SETD1A
BOD1L
BOD1L/SETD1A
% of input
Antibody:
IgG
53BP1
RIF1
Untreated
ER-mCherry-LacI-FokI-DD
LacO repeats
p1
p2
p3
p4

Figure S6, related to Figure 6: H3K4 methylation promotes RIF1 recruitment to DNA double-strand breaks and supresses BRCA1-mediated end resection. (A) Representative images of ionizing radiation-induced RIF1, RPA2 and BRCA1 foci in H3-GFP WT and K4A from Figure 6B-G. Scale bars = 10 µm. **(B)** Radial chromosome formation in cells from Figure 6H was analysed by Giemsa staining and light microscopy. **(C)** Cells from Figure 6H were harvested 24 h post olaparib treatment, immunostained with an antibody against RAD51, and foci formation enumerated by fluorescence microscopy. **(D)** H3-GFP WT and K4A cells were exposed to ionizing radiation (IR), immunostained as above, and foci formation enumerated. **(E-F)** HeLa cells were transfected with the indicated siRNAs, exposed to IR, and formation of RIF1 or RPA2 foci monitored as above. **(G)** Representative images of HeLa cells from Figure 6K. Arrows denote transfected cells. **(H)** U-2-OS-FokI cells were treated with 4-OHT and/or doxycycline, chromatin was isolated and ChIP was perfromed with the indicated antibodies and then quantified by qPCR. Data represent the average signal across the four primer pairs represented in the schematic in Figure 6N or Figure S6J. **(I)** Quantification of PLA signals between γH2AX and H3K4me3 in HeLa cells transfected with the indicated siRNAs and exposed to IR. **(J)** U-2-OS-FokI cells were transfected with the indicated siRNAs, undamaged chromatin was isolated and ChIP was performed with the indicated antibodies and quantified by qPCR. Data represent the average signal across the four primer pairs represented in the schematic. In all cases n=3 biologically independent experiments. Error bars indicate mean ± s.e.m. *P* values, unpaired two-tailed *t*-test except (B and J) (Mann Whitney), $^{*}p \leq 0.05$, $^{**}p \leq 0.01$ and $^{***}p \leq 0.001$.

Figure S7:

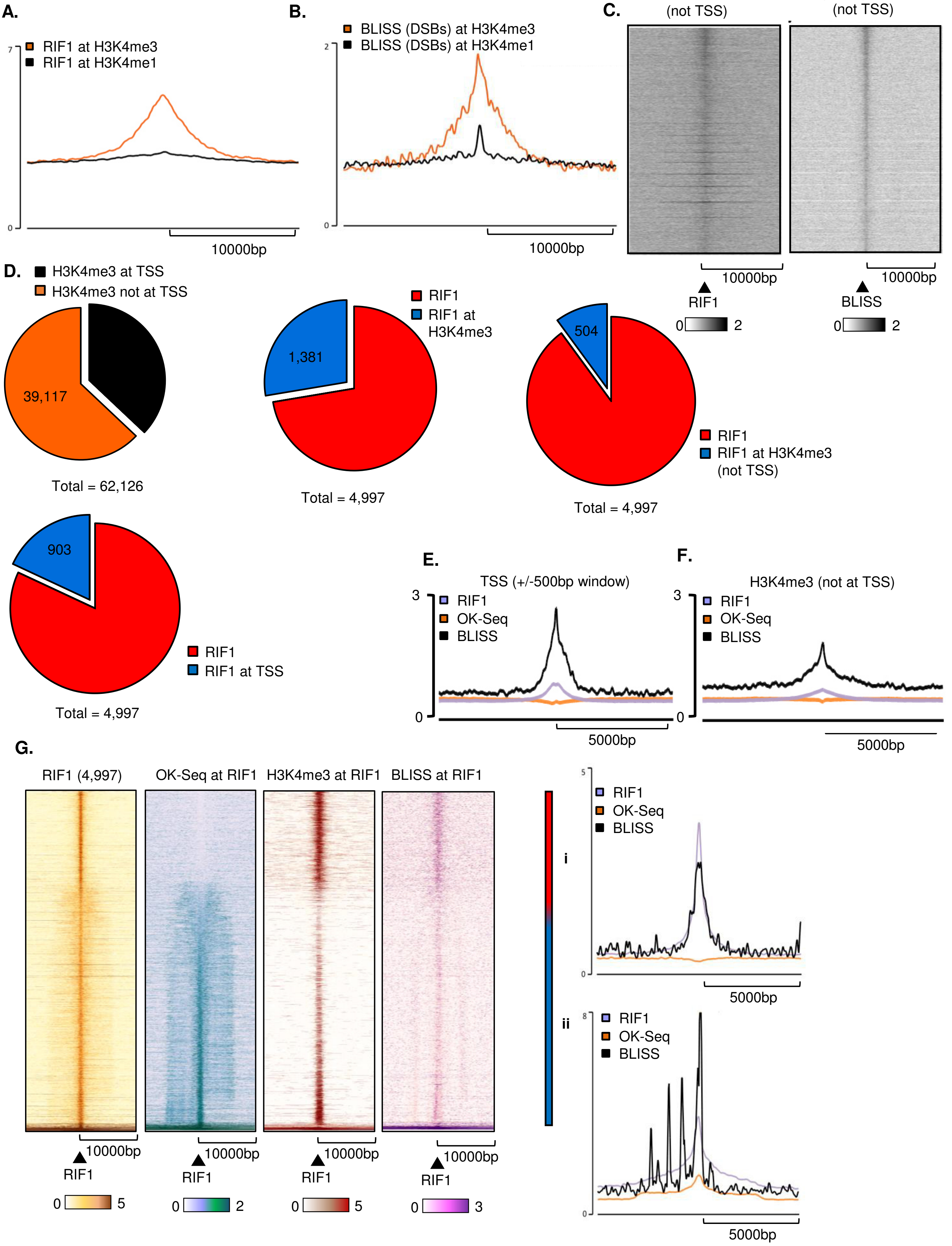
A.
RIF1 at H3K4me3
RIF1 at H3K4me1
10000bp
B.
BLISS (DSBs) at H3K4me3
BLISS (DSBs) at H3K4me1
10000bp
C.
RIF1 at H3K4me3 (not TSS)
BLISS (DSBs) at H3K4me3 (not TSS)
10000bp
RIF1
BLISS
D.
H3K4me3 at TSS
H3K4me3 not at TSS
39,117
Total = 62,126
RIF1
RIF1 at H3K4me3
1,381
Total = 4,997
504
RIF1
RIF1 at H3K4me3 (not TSS)
Total = 4,997
903
RIF1
RIF1 at TSS
Total = 4,997
E.
TSS (+/-500bp window)
RIF1
OK-Seq
BLISS
5000bp
F.
H3K4me3 (not at TSS)
RIF1
OK-Seq
BLISS
5000bp
G.
RIF1 (4,997)
OK-Seq at RIF1
H3K4me3 at RIF1
BLISS at RIF1
10000bp
RIF1
i
ii
RIF1
OK-Seq
BLISS
5000bp

Figure S7, related to Figure 7: RIF1 coincides with sites of endogenous DSBs marked by H3K4me3 in mouse ESCs. (A-B) Chromatin immunoprecipitation profiles of murine RIF1 and profiles of BLISS DSB signals at peak sites of H3K4me1 and H3K4me3. **(C)** Genomic profiles of RIF1 chromatin immoprecipitation and BLISS signals over H3K4me3-positive areas that lie outside areas defined by ENCODE as TSS. **(D)** Overlap of chromatin immunoprecipitation peaks sites of murine RIF1, H3K4me3 or ENCODE TSS. Total number of peaks is denoted below. **(E-F)** Chromatin immunoprecipitation profiles of murine RIF1, profiles of BLISS DSB signals and Okazaki fragment sequencing (OK-seq) profiles at ENCODE TSS (E) or peak sites of H3K4me3 (F). **(G)** Genomic profiles of BLISS signals, H3K4me3 chromatin immunoprecipitation profiles and OK-seq profiles over RIF1 peak sites. Profiles are then subdivided into 2 groups with profiles of Rif, BLISS and OK-seq signals denoted: (i) areas with no OK-seq signal and strong BLISS and H3K4me3 signals; (ii) areas with a strong OK-seq signal and variable BLISS and H3K4me3 signals. Data is from Yan *et al.,* 2017 and Petruk *et al.,* 2018.

Table S1:

**Figure**	**Number and genotype of animals**
Fig 5A	6 *Bod1l*$^{+/+}$ *Cd19*$^{+/Cre}$ (3♀; 3♂) and 6 *Bod1l*$^{F/F}$ *Cd19*$^{+/Cre}$ (4♀; 2♂)
Fig B-C	2 *Bod1l*$^{F/F}$ *R26*$^{+/+}$ (1♀; 1♂) and 3 *Bod1l*$^{F/F}$ *R26*$^{CreERT2/+}$ (4♀; 1♂)
Fig 5D and S5E-F	6 *Bod1l*$^{+/+}$ *Cd19*$^{+/Cre}$ (3♀; 3♂) and 8 *Bod1l*$^{F/F}$ *Cd19*$^{+/Cre}$ (5♀; 3♂)
Fig 5E-F	5 *Bod1l*$^{+/+}$ *Cd19*$^{+/Cre}$ (3♀; 2♂) and 8 *Bod1l*$^{F/F}$ *Cd19*$^{+/Cre}$ (3♀; 5♂)

Table S1, related to STAR Methods: Details of the animals used in the study. The individual numbers, genotype and gender of the animals used for this study are detailed. ♂ denotes male, ♀ denotes female.
