# Supplementary material for: H3K4 methylation by SETD1A/BOD1L facilitates RIF1-dependent NHEJ
Source: Mol Cell. 2022 May 19;82(10):1924–1939.e10. doi: 10.1016/j.molcel.2022.03.030 (PMC9616806; doi:10.1016/j.molcel.2022.03.030)
Supplement: Document S2. Article plus supplemental information [file mmc2.pdf]

Molecular Cell

Article

H3K4 methylation by SETD1A/BOD1L facilitates RIF1-dependent NHEJ

Graphical abstract

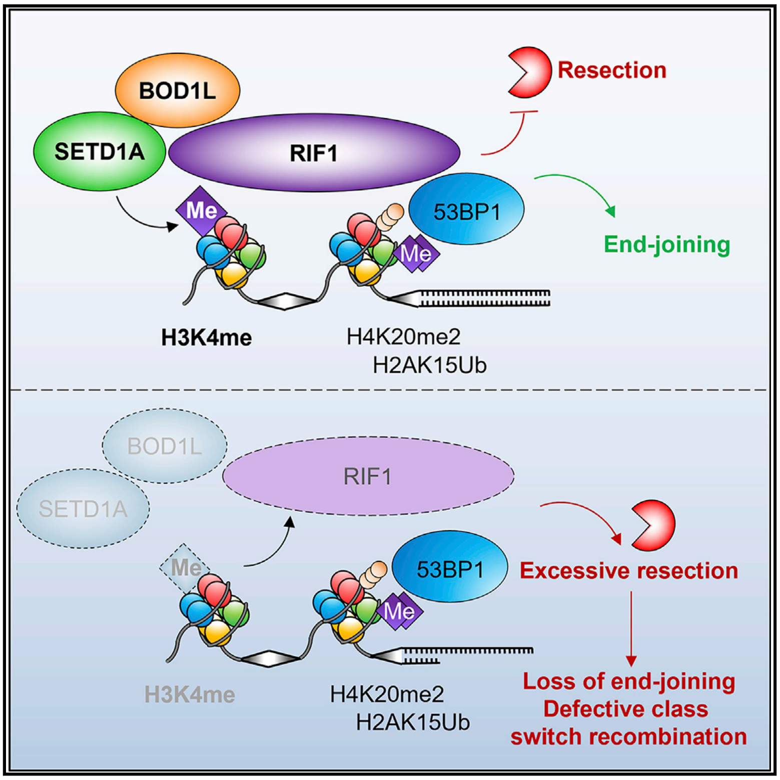

Authors

Rachel Bayley, Valerie Borel, Rhiannon J. Moss, ..., Grant S. Stewart, Simon J. Boulton, Martin R. Higgs

Correspondence

g.s.stewart@bham.ac.uk (G.S.S.),
simon.boulton@crick.ac.uk (S.J.B.),
m.r.higgs@bham.ac.uk (M.R.H.)

In brief

Bayley et al. identify histone H3K4 methylation by SETD1A as vital for DNA repair, by promoting RIF1 accumulation at damaged sites. Deficiencies in H3 methylation or the SETD1A-BOD1L complex impairs end-joining of broken DNA ends, abrogates class switch recombination, promotes uncontrolled end-resection, and compromises the efficacy of PARP inhibitors.

Highlights

- BOD1L, SET1A, and H3K4me3 promote RIF1 accumulation at DNA break sites
- SETD1A-dependent H3K4 methylation promotes end-joining and suppresses end-resection
- Perturbing SETD1A confers resistance to PARP inhibitors in BRCA1-deficient cells
- RIF1 binds directly to methylated H3K4

Bayley et al., 2022, Molecular Cell *82*, 1924–1939
May 19, 2022 © 2022 The Author(s). Published by Elsevier Inc.
https://doi.org/10.1016/j.molcel.2022.03.030

Article

H3K4 methylation by SETD1A/BOD1L facilitates RIF1-dependent NHEJ

Rachel Bayley,[1,3] Valerie Borel,[2,3] Rhiannon J. Moss,[1,4] Ellie Sweatman,[1] Philip Ruis,[2,5] Alice Ormrod,[1] Amalia Goula,[1] Rachel M.A. Mottram,[1] Tyler Stanage,[2] Graeme Hewitt,[2,6] Marco Saponaro,[1] Grant S. Stewart,[1,*] Simon J. Boulton,[2,*] and Martin R. Higgs[1,7,*]
[1]Institute of Cancer and Genomic Sciences, University of Birmingham, Birmingham B15 2TT, UK
[2]DSB Repair Metabolism Laboratory, The Francis Crick Institute, Midland Road, London, UK
[3]These authors contributed equally
[4]Present address: School of Biosciences, University of Birmingham, Birmingham B15 2TT, UK
[5]Present address: Imperial College London, Exhibition Rd, South Kensington, London SW7 2BU, UK
[6]Present address: School of Cancer & Pharmaceutical Sciences, King's College London, London SE1 9RT, UK
[7]Lead contact
*Correspondence: g.s.stewart@bham.ac.uk (G.S.S.), simon.boulton@crick.ac.uk (S.J.B.), m.r.higgs@bham.ac.uk (M.R.H.)
https://doi.org/10.1016/j.molcel.2022.03.030

SUMMARY

The 53BP1-RIF1-shieldin pathway maintains genome stability by suppressing nucleolytic degradation of DNA ends at double-strand breaks (DSBs). Although RIF1 interacts with damaged chromatin via phospho-53BP1 and facilitates recruitment of the shieldin complex to DSBs, it is unclear whether other regulatory cues contribute to this response. Here, we implicate methylation of histone H3 at lysine 4 by SETD1A-BOD1L in the recruitment of RIF1 to DSBs. Compromising SETD1A or BOD1L expression or deregulating H3K4 methylation allows uncontrolled resection of DNA ends, impairs end-joining of dysfunctional telomeres, and abrogates class switch recombination. Moreover, defects in RIF1 localization to DSBs are evident in patient cells bearing loss-of-function mutations in SETD1A. Loss of SETD1A-dependent RIF1 recruitment in *BRCA1*-deficient cells restores homologous recombination and leads to resistance to poly(ADP-ribose)polymerase inhibition, reinforcing the clinical relevance of these observations. Mechanistically, RIF1 binds directly to methylated H3K4, facilitating its recruitment to, or stabilization at, DSBs.

INTRODUCTION

DNA double-strand breaks (DSBs) are one of the most serious forms of DNA damage. They arise spontaneously due to replication fork collapse or after excessive oxidative damage and are induced following exposure to ionizing radiation (IR). DSBs are also formed in a programmed manner during immune system development; indeed, their induction and repair is essential for both V(D)J and class switch recombination (CSR). DSBs are repaired via two principal pathways: homologous recombination (HR) and non-homologous end-joining (NHEJ). While NHEJ involves positioning broken DNA ends in close proximity to enable direct ligation, HR requires end-resection and an intact homologous template for repair and is thus restricted to S/G2 phases. The initial processing of DSBs is therefore a key determinant of repair: in G1, end-resection is suppressed, and DNA ends are protected to favor NHEJ, while resection is activated in S/G2 to allow HR.

The 53BP1-RIF1-shieldin pathway is vital to determine how DSBs are repaired by counteracting end-resection. Critical to this are 53BP1 itself (Bothmer et al., 2010; Bunting et al., 2010; Difilippantonio et al., 2008), the 53BP1 interactors RIF1 (Chapman et al., 2013; Escribano-Díaz et al., 2013; Zimmermann et al., 2013) and PTIP (Callen et al., 2020; Daniel et al., 2010), and the downstream effector complex REV7-shieldin (Boersma et al., 2015; Dev et al., 2018; Ghezraoui et al., 2018; Noordermeer et al., 2018; Xu et al., 2015). Loss of any of these factors leads to unrestrained end-resection of G1 DSBs, impairing NHEJ and abrogating CSR in B lymphocytes. Functionally, these proteins antagonize end-resection driven by the pro-HR tumor suppressor BRCA1. Deregulation of this pathway therefore has important clinical implications in HR-deficient cancer cells such as those lacking BRCA1. Such cells are hypersensitive to poly(ADP-ribose)polymerase (PARP) inhibitors (PARPis), but loss of 53BP1, RIF1, or REV7-shieldin confer resistance to PARPi by restoring HR.

The chromatin environment is also a key regulator of DSB repair, and numerous chromatin modifiers and histone post-translational modifications (PTMs) play important roles in HR/NHEJ (Ferrand et al., 2021). This is exemplified by roles for H4K20 di-methylation and H2AK15 mono-ubiquitination in regulating 53BP1 and NHEJ. 53BP1 binds pre-existing H4K20me2 and damage-induced H2AK15Ub via its TUDOR and UDR domains, respectively, promoting its recruitment to DSBs (Fradet-Turcotte et al., 2013; Wilson et al., 2016). Conversely, H2AK15Ub and non-methylated

 © 2022 The Author(s). Published by Elsevier Inc.
This is an open access article under the CC BY license (http://creativecommons.org/licenses/by/4.0/).

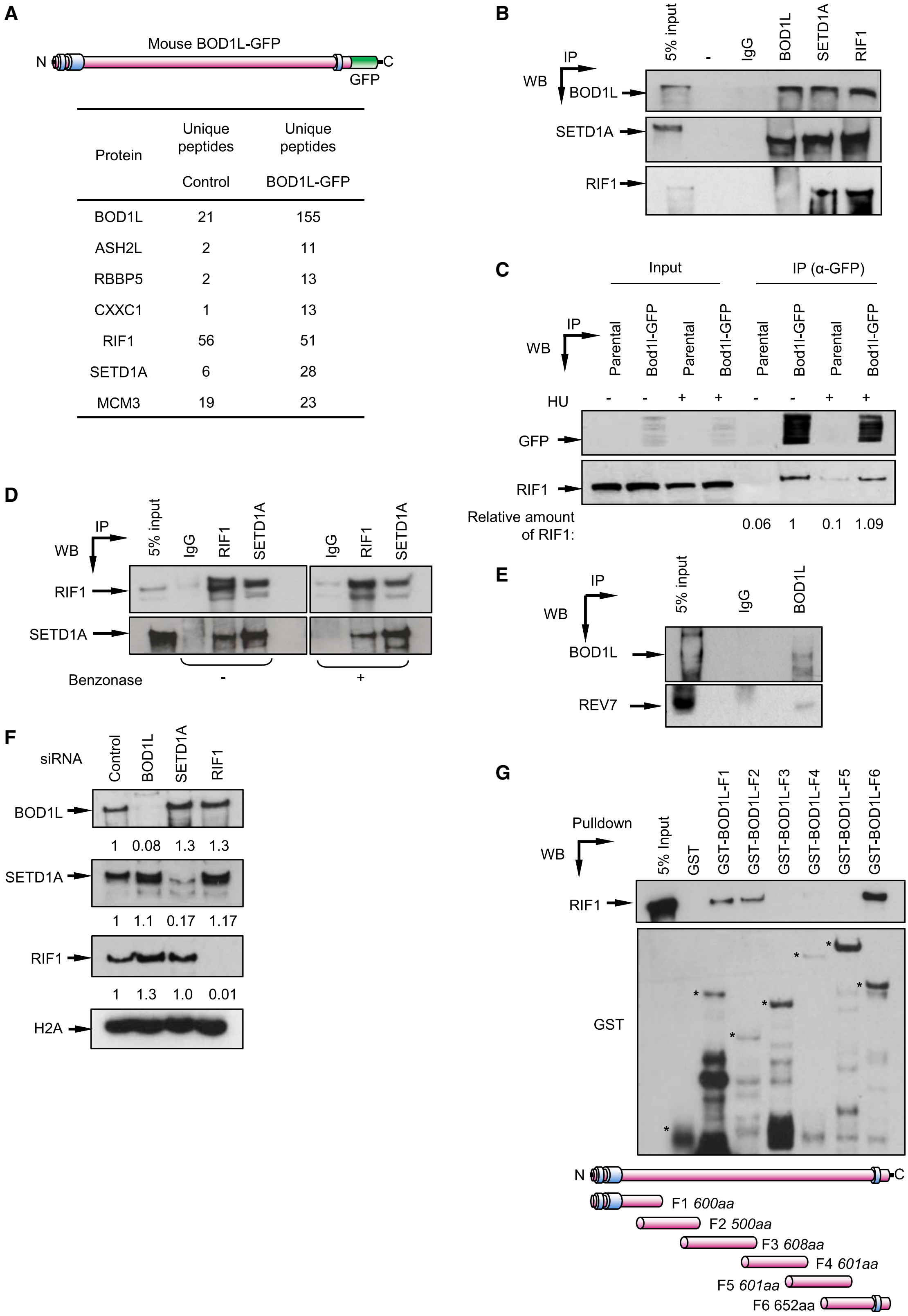

(legend on next page)

H4K20 provides a binding site for BRCA1-BARD1, suppressing 53BP1 recruitment and stimulating end-resection in post-replicative cells (Nakamura et al., 2019). Moreover, other histone PTMs negatively regulate 53BP1 binding such as H4K16 acetylation (Tang et al., 2013).

We previously implicated the chromatin modifier SETD1A in maintaining genome stability after replication stress by protecting stalled replication forks (Higgs et al., 2018). SETD1A is a lysine methyltransferase that methylates Lys 4 of histone H3 (H3K4) to regulate transcription, haematopoiesis, neurological function, and DNA repair (Higgs et al., 2018; Hoshii et al., 2018; Kranz and Anastassiadis, 2020). SETD1A exists as part of the multimeric COMPASS complex (complex of proteins associated with Set1) comprising several enzymatic co-factors and the scaffold protein BOD1L (encoded by the *BOD1L1* gene). Importantly, both BOD1L and the methyltransferase activity of SETD1A are required to protect nascent DNA (Higgs et al., 2015, 2018). Interestingly, BOD1L was first identified as a target for the apical DNA repair kinases ATM/ATR (Matsuoka et al., 2007), suggesting that damage-inducible PTMs may control COMPASS function.

Although the PTMs that govern 53BP1 chromatin recruitment are well characterized, less is known about how downstream factors such as RIF1 are regulated. Recent studies have uncovered a phospho-binding role for RIF1 toward phosphorylated 53BP1 (Setiaputra et al., 2022). Here, we demonstrate that RIF1 also physically and functionally interacts with BOD1L and SETD1A, which are required for its recruitment to DSBs. Cells lacking BOD1L or SETD1A, including those from patients harboring loss-of-function mutations in SETD1A, exhibit elevated end-resection in G1 and impaired NHEJ-mediated fusion of dysfunctional telomeres. Furthermore, genetic deletion of mouse *Bod1L* led to defective CSR in B lymphocytes, and loss of SETD1A in *BRCA1*-deficient cells confers PARPi resistance. Crucially, we show that RIF1 directly binds to methylated H3K4. Compromising SETD1A-dependent histone methylation therefore abrogates RIF1 recruitment to DSBs, increases BRCA1-dependent end-resection and gives rise to PARPi resistance in the absence of *BRCA1*. Taken together, our data establish that SETD1A-dependent H3K4 methylation plays a key role in DSB repair by promoting RIF1 recruitment to sites of DNA damage to suppress end-resection.

RESULTS

BOD1L and SETD1A interact with RIF1

BOD1L was first identified in a phospho-proteomic screen as a target for the damage-responsive kinases ATM/ATR (Matsuoka et al., 2007). BOD1L is also implicated in the replication stress response where it functionally interacts with SETD1A to protect stalled replication forks (Higgs et al., 2015, 2018). To gain further insights into how BOD1L maintains genome stability, we performed mass spectrometry to identify interacting partners of BOD1L. Consistent with previous findings (Higgs et al., 2018), several members of the COMPASS-SETD1A complex including SETD1A, ASH2L, CXXC1, and RBBP5 were enriched in murine BOD1L-GFP immunoprecipitates (Figure 1A). Unexpectedly, the pro-NHEJ and replication timing factor RIF1 was also present in these complexes. These findings were confirmed by reciprocal co-immunoprecipitation (Figures 1B and 1C), with the interaction evident in unperturbed conditions and not mediated by DNA (Figures 1C and 1D). This suggested potentially unexpected roles for BOD1L and SETD1A in DSB repair. In agreement, the RIF1 and shieldin interactor REV7 (MAD2L2) was also present in BOD1L immunoprecipitates (Figure 1E), suggesting that BOD1L and SETD1A might participate in this pro-NHEJ pathway. Importantly, depletion of BOD1L or SETD1A did not affect RIF1 protein expression (Figure 1F), suggesting that they were not required to stabilize RIF1.

Since the N terminus of BOD1L facilitates its interaction with SETD1A, likely via its "Shg1 homology" region (Higgs et al., 2018), we hypothesized that this region may also mediate RIF1 binding. To assess this, we generated GST-tagged fragments spanning ~500-aa regions of BOD1L and analyzed their ability to interact with RIF1. Surprisingly, regions within both the N and C termini of BOD1L could support RIF1 binding (Figure 1G), suggesting a conformation-dependent interaction. Collectively, these data raise the possibility that BOD1L and SETD1A may cooperate with and/or modulate the functions of RIF1 during DSB repair.

BOD1L and SETD1A promote RIF1 recruitment to DSBs

Prompted by these observations, we set out to analyze the consequences of depleting these factors from cells on DSB repair. We first monitored IR-induced foci (IRIF) of RIF1 in these cells to ascertain whether BOD1L or SETD1A affected recruitment of RIF1 to DSBs. Strikingly, the depletion of both factors using siRNA reduced RIF1 IRIF in G1-phase cells, but not in S/G2 (Figures 2A, 2B, and S1A–S1C). This was supported by findings from cells in which targeted DSBs were induced within a Lac-operator array by the mCherry-lacI-*FokI* nuclease (Figures 2C and 2D), and from mouse embryonic fibroblasts (MEFs) in which *Bod1l* had been genetically ablated using tamoxifen-regulated Cre (Figures S1D and S1E). Moreover, defects in RIF1 IRIF were also observed in lymphoblastoid cells

Figure 1. BOD1L and SETD1A interact with RIF1

(A) Murine BOD1L-GFP complexes from HeLa cells were analyzed by mass spectrometry. Unique peptide counts of selected hits are shown.

(B) HeLa nuclear cell extracts were immunoprecipitated with the denoted antibodies, and inputs and immunoprecipitates analyzed by immunoblotting.

(C) Whole-cell extracts (WCEs) of HeLa cells expressing mouse BOD1L-GFP were immunoprecipitated with the denoted antibodies in the presence/absence of hydroxyurea, and inputs and immunoprecipitates analyzed as above.

(D) RIF1 was immunoprecipitated from HeLa WCE in the presence/absence of benzonase, and inputs and immunoprecipitates analyzed as above.

(E) BOD1L was immunoprecipitated from HeLa WCE, and inputs and immunoprecipitates analyzed as above.

(F) HeLa cells were transfected with the indicated siRNAs for 72 h, and WCE analyzed by immunoblotting. Protein levels were quantified by ImageJ and expressed as a ratio compared with control cells.

(G) HeLa nuclear cell extracts were incubated with GST or GST-BOD1L fragments, complexes were isolated by glutathione-sepharose and analyzed by immunoblotting. Data in all cases are representative of ≥2 independent experiments.

CellPress
OPEN ACCESS

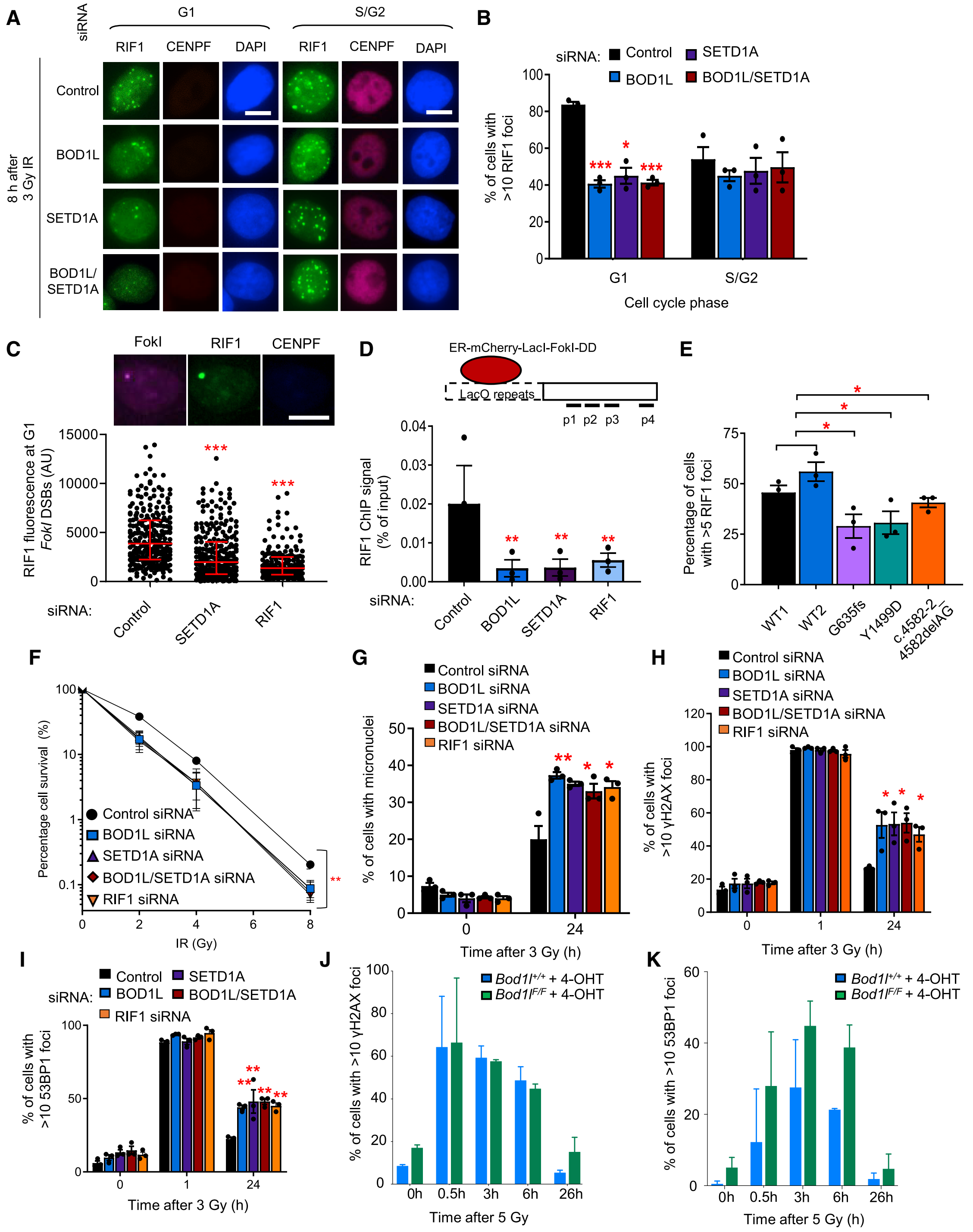

Figure 2. BOD1L and SETD1A are required for RIF1 recruitment to DSBs and for efficient DSB repair

(A and B) HeLa cells were transfected with the indicated siRNAs for 72 h, exposed to ionizing radiation (IR), and immunostained with antibodies to CENPF and RIF1. Representative fluorescence microscopy images are shown (A); scale bars, 10 μm. Foci formation was quantified (B).

(C) U-2-OS-FokI cells were transfected with the indicated siRNAs for 48 h, treated with 4-OHT and immunostained with antibodies to CENPF and RIF1. Representative images are shown above (scale bars, 10 μm), and fluorescence intensity per FokI-focus was quantified using ImageJ. Lines denote mean values from three independent experiments.

(D) Chromatin isolated from cells in (C) was immunoprecipitated with the denoted antibodies and quantified by qPCR. A schematic of the relative positions of 4 qPCR amplicons is shown, and normalized amounts of RIF1 bound at FokI-induced double-strand breaks in cells across all amplicons is indicated.

(legend continued on next page)

lines (LCLs) derived from neuropsychiatric patients with heterozygous loss-of-function SETD1A mutations (Kummeling et al., 2021; Figures 2E, S1F, and S1G). Furthermore, recruitment of the downstream effector REV7 to DSBs was also compromised in cells lacking either BOD1L or SETD1A (Figure S1H), although the inability of these cells to form RIF1 or REV7 foci could not be explained by a failure to recruit 53BP1 to DSBs (Figures S1I–S1L).

We next examined whether SETD1A and BOD1L localized to sites of DNA damage. Interestingly, although neither protein formed IRIF (data not shown), both proteins localized to *FokI*-induced DSBs by chromatin immunoprecipitation (ChIP) (Figure S2A). We also used proximity ligation assays to explore any hierarchical relationships in this recruitment cascade. In line with our previous data, these revealed that RIF1 recruitment to damaged chromatin was dependent on SETD1A and BOD1L. However, the recruitment/retention of these factors to sites of DNA damage was governed by a complex interdependent relationship (Figures S2B–S2D), as SETD1A recruitment to DSB sites was dependent on RIF1, BOD1L, and 53BP1. In broad agreement, SETD1A and BOD1L were also required for the localization of RIF1 to damaged replication forks (Figure S2E), likely linked to their similar roles in protecting stalled forks from DNA2-dependent degradation (Garzón et al., 2019; Higgs et al., 2015, 2018; Mukherjee et al., 2019). Together, these data demonstrate that SETD1A and BOD1L functionally interact with RIF1 to promote its accumulation at sites of damage.

BOD1L and SETD1A are required for efficient DSB repair

Given that both BOD1L and SETD1A localize to DSBs and are required for efficient RIF1 recruitment to these lesions, we postulated that they would be required for DSB repair. We therefore depleted them from HeLa cells using siRNA and analyzed the ultimate impact on DSB repair and cell survival after exposure to IR. Depletion of SETD1A alone or in combination with BOD1L increased cellular radiosensitivity (Figure 2F), and elevated IR-induced genome instability (Figure 2G). Furthermore, these cells failed to efficiently repair IR-induced DSBs, as denoted by the persistence of γH2AX and 53BP1 foci at late time points post-irradiation (Figures 2H, 2I, S2F, S2G, and S3). Cre-mediated genetic ablation of *Bod1l* from MEFs also led to unrepaired DSBs persisting late after IR exposure (Figures 2J, 2K, S2H, S2I, and S3). These observations suggest that dysfunctional recruitment of RIF1 to DSBs in the absence of SETD1A or BOD1L compromises DNA repair and promotes genome instability.

SETD1A, BOD1L, and RIF1 act together to antagonize BRCA1-dependent resection

Since the RIF1-53BP1-shieldin pathway counteracts nucleolytic degradation of DNA ends at G1 DSBs (Boersma et al., 2015; Bothmer et al., 2010; Bunting et al., 2010; Chapman et al., 2013; Dev et al., 2018; Difilippantonio et al., 2008; Escribano-Díaz et al., 2013; Ghezraoui et al., 2018; Noordermeer et al., 2018; Xu et al., 2015; Zimmermann et al., 2013), we investigated whether BOD1L and SETD1A shared this function. Depletion of either factor from HeLa cells or genetic ablation of *Bod1l* in MEFs resulted in substantially elevated levels of end-resection after IR exposure or *FokI* induction, as revealed by increased RPA2 S4/8 phosphorylation and/or RPA2 focus formation (Figures 3A–3E and S4A–S4C). We also observed an increased number of DSBs undergoing resection in cells depleted of BOD1L, SETD1A, or RIF1, as judged by increased levels of native IdU foci per cell (Figure 3F), while there was no effect on the length of resected DNA tracts (Figure 3G), suggesting that BOD1L or SETD1A affected the interplay between HR and NHEJ. Co-depletion of BOD1L and SETD1A, or loss of RIF1, had no additional effect on any of the phenotypes observed (Figures 3F–3H), further confirming that these factors function together.

Unrestrained end-resection in the absence of the RIF1-53BP1-shieldin pathway is due to aberrant accumulation of BRCA1 in G1 and subsequent CtIP- and MRE11-dependent processing. In keeping with potential roles in this pathway, loss or haploinsufficiency of SETD1A or BOD1L alone or in the absence of RIF1 increased recruitment of BRCA1 to G1 DSBs (Figures 3I–3K and S4D). We also observed increased G1-phase RAD51 focus formation after depletion of SETD1A, BOD1L, or RIF1 (Figure 3L), consistent with increased BRCA1-dependent end-resection. Elevated end-resection in these cells was dependent on CtIP and MRE11 (Figures S4E and S4F), in keeping with previous observations (Biehs et al., 2017). Importantly, defective RIF1 localization to G1 DSBs was not due to unrestrained antagonism by BRCA1, as co-depletion of BRCA1 and SETD1A had no restorative effect on this phenotype (Figure S4G). Finally, depletion of REV7 had no additional impact on the formation of either RIF1 or BRCA1 IRIF in cells lacking SETD1A (Figures S4H and S4I). Together, these findings suggest that RIF1, BOD1L, and SETD1A act together to suppress inappropriate BRCA1-dependent DNA end-resection at G1 DSBs.

BOD1L and SETD1A promote NHEJ

Given the established role of RIF1 in end-joining, we posited that SETD1A and BOD1L would also act as pro-NHEJ factors. To test this, we exploited cells that can be conditionally inactivated for the Shelterin subunit TRF2, triggering telomere deprotection and eliciting chromosome end-to-end fusions mediated by NHEJ. Loss of pro-NHEJ factors such as RIF1 in this system attenuates these fusions. Strikingly, BOD1L or SETD1A also suppressed the fusion of dysfunctional telomeres following TRF2 deletion, or after expression of a dominant-negative

(E) Patient LCL cells haploinsufficient for SETD1A were exposed to IR, immunostained with an antibody to RIF1, and foci formation enumerated.
(F) HeLa cells were transfected with the indicated siRNAs, irradiated, left to form colonies for 14 days, and then stained with methylene blue and colonies counted.
(G) HeLa cells were transfected as in (F), exposed to IR, left for 24 h, and micronuclei formation assessed.
(H and I) HeLa cells from (F) were irradiated, immunostained with antibodies to γH2AX (H) or 53BP1 (I), and foci formation enumerated.
(J and K) *Bod1l*$^{F/F}$ and *Bod1l*$^{+/+}$ MEFs were treated with 4-OHT, irradiated, immunostained with antibodies against γH2AX (J) or 53BP1 (K), and foci formation quantified. Plots in all cases represent data from three independent experiments; error bars = mean ± SEM, p values: unpaired two-tailed t tests except (C) (Mann-Whitney) and (F) (two-way ANOVA). *$p \leq 0.05$, **$p \leq 0.01$ and ***$p \leq 0.001$. See also Figures S1–S3.

CellPress
OPEN ACCESS

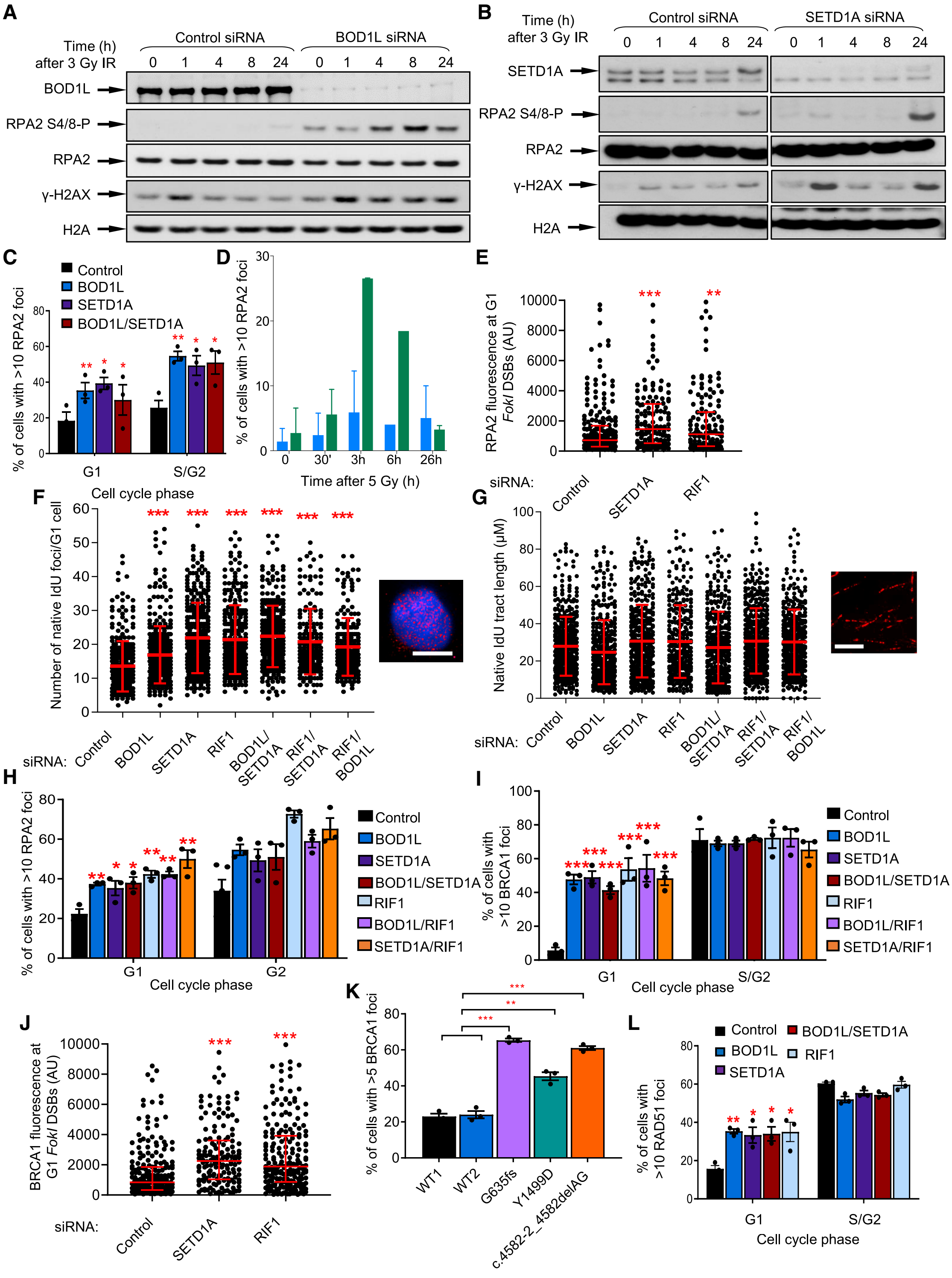

Figure 3. BOD1L and SETD1A suppress DSB resection

(A and B) HeLa cells were transfected with the indicated siRNAs, exposed to IR, harvested at the indicated times, and WCE were analyzed by immunoblotting.

(C) HeLa cells from (A) and (B) were immunostained with antibodies to CENPF and RPA2, and foci formation enumerated.

(D) *Bod1l*$^{F/F}$ and *Bod1l*$^{+/+}$ MEFs were treated with 4-OHT, irradiated, immunostained with antibodies against RPA2, and foci formation enumerated.

(E) U-2-OS-FokI cells were transfected with the indicated siRNAs, treated with 4-OHT and immunostained with antibodies to CENPF and RPA2. Fluorescence intensity per FokI-focus was quantified using ImageJ. Lines denote mean values from three independent experiments.

(legend continued on next page)

ΔBΔM version of TRF2 (Figure 4A–4D). Although NHEJ-dependent repair was compromised by loss of BOD1L or SETD1A, HR-mediated repair was unaffected (Figure 4E).

In line with these findings, depletion of SETD1A and/or RIF1 also decreased the sensitivity of *BRCA1*-deficient cells to PARPis by partially rescuing toxic NHEJ-mediated radial formation (Figures 4F–4H). This was accompanied by reinstatement of PARPi-induced RAD51 focus formation (Figure 4I), and partial restoration of functional HR (Figure 4J). Furthermore, the increased RIF1 foci formation apparent in *BRCA1*-deficient S/G2 cells (Escribano-Díaz et al., 2013) was reliant on SETD1A and BOD1L (Figure 4K). Thus, SETD1A and BOD1L promote NHEJ, and their loss is sufficient to abrogate end-joining and restore HR in *BRCA1*-deficient cells, suppressing PARPi efficacy.

One alternative mechanism for PARPi resistance in *BRCA1*-deficient cells is the restoration of defective replication fork protection (Ray Chaudhuri et al., 2016). Since BOD1L, SETD1A, and RIF1 are all required to protect stalled replication forks from DNA2-dependent degradation, we set out to examine whether loss of these factors in *BRCA1*-deficient cells also restored fork protection. Notably, depletion of either RIF1, BOD1L, or SETD1A on a BRCA1-deficient background failed to prevent degradation of nascent DNA (Figure 4L), suggesting that resistance to PARPi in these cells was specifically due to restoration of HR.

BOD1L is necessary for CSR *in vivo*

The 53BP1-RIF1-shieldin pathway also plays a vital role in the long-range joining of physiologically-induced DSBs, such as those generated during CSR (Di Virgilio et al., 2013; Difilippantonio et al., 2008). Since hematopoietic loss of *Setd1a* blocks B cell development (Tusi et al., 2015), we focused on the role of *Bod1l* in these processes. To this end, we conditionally ablated *Bod1l* specifically from murine B cells using Cd19 deleter Cre (Figure S5A), which significantly decreased the levels of pre-immune IgG in the serum (Figure 5A). To determine whether this decrease was caused by a CSR defect, we harvested B cells from tamoxifen-inducible conditional *Bod1l* mice fed with 4-hydroxytamoxifen (4-OHT) and stimulated them with IL4, LPS, and/or anti-CD40 (Figure S5B). Loss of BOD1L substantially reduced CSR, denoted by decreased production of all IgG isotypes tested and of IgE, which was independent of the number of cell divisions occurring during stimulation (Figures 5B, 5C, S5C, and S5D). Similar results were obtained when BOD1L was specifically depleted from B cells (Figures 5D, S5E, and S5F). To investigate this defect further, mice were immunized with NP-CGG and NP-specific immunoglobulins were quantified. While levels of anti-NP IgM were unaffected in *Bod1l*-deficient mice, levels of IgG1 were decreased 7–14 days post-immunization (Figures 5E and 5F). These data demonstrate that, similar to RIF1, 53BP1, and REV7-shieldin, BOD1L is important for facilitating physiological end-joining during CSR.

H3K4 methylation by SETD1A facilitates RIF1-dependent repair

Enzymatically, SETD1A methylates lysine 4 of histone H3 (H3K4). We therefore examined whether H3K4 methylation may directly affect RIF1-dependent end protection, using HeLa cell lines expressing either wild-type (WT) GFP-tagged histone H3 or a Lys4Ala (K4A) mutant (Higgs et al., 2018; Sato et al., 2012). Importantly, expression of this K4A variant recapitulated the phenotypes observed in cells lacking BOD1L, SETD1A, or RIF1, including increased IR sensitivity, defective RIF1 and REV7 IRIF formation in G1, increased BRCA1-dependent end-resection and decreased PARPi sensitivity following BRCA1 depletion (Figures 6A–6H and S6A–S6D). Depletion of SETD1A had no additional effect in H3K4A-expressing cells (Figures 6I and 6J), reinforcing our conclusion that SETD1A facilitates RIF1 localization to DSBs via H3K4 methylation. However, depletion of other related KMT2 methyltransferases, all of which target H3K4, had no effect on RIF1 or RPA IRIF formation (Figures S6E and S6F), demonstrating a specific role for SETD1A in this process. We also made use of a complementary system in which overexpression of KDM5A, an H3K4 demethylase, reduces H3K4 levels independently of SETD1A depletion. Crucially, overexpression of WT KDM5A, but not a catalytically inactive mutant (H483A), also affected RIF1 and BRCA1 recruitment to G1 DSBs in a similar fashion to loss of BOD1L or SETD1A (Figures 6K and S6G). Furthermore, exogenous expression of WT SETD1A but not a variant lacking the catalytic SET domain (ΔSET) reinstated normal RIF1 and BRCA1 IRIF in SETD1A-depleted cells, establishing that the methyltransferase activity of SETD1A is required to regulate end protection (Figures 6L and 6M). Therefore, SETD1A-dependent H3K4 methylation is required for RIF1 DSB recruitment.

We next set out to ascertain whether H3K4 methylation is a pre-existing or damage-inducible requirement for RIF1 recruitment to DSBs. To this end, we first examined levels of H3K4 methylation on a Lac-operator in the presence/absence of DSBs (induced by mCherry-lacI-*FokI*) or transcription (induced by doxycycline) using ChIP. In agreement with previous reports (Li and Tyler, 2016; Zheng et al., 2018), DSB induction evicted histone H3. Furthermore, although H3K4me3 was present on undamaged chromatin, relative levels of H3K4me3 surrounding newly-formed DSBs were increased (Figures 6N

(F and G) HeLa cells were transfected with the indicated siRNAs, pulsed with IdU for 24 h, exposed to IR for 1 h, and labeled with anti-IdU antibody. Native tract length (F) or native IdU foci (G) were calculated or enumerated. Lines denote mean values from three independent experiments, and representative images are shown (scale bars, 10 μm).

(H and I) HeLa cells from (F) were exposed to IR, immunostained with antibodies to either CENPF and RPA2 (H) or CENPF and BRCA1 (I), and foci formation assessed.

(J) U-2-OS-FokI cells were transfected with the indicated siRNAs, treated with 4-OHT and immunostained with antibodies to CENPF and BRCA1. Fluorescence intensity per FokI-focus was quantified using ImageJ. Lines denote mean values from three independent experiments.

(K) SETD1A patient LCL cells were exposed to IR, immunostained with an antibody to BRCA1, and foci formation enumerated.

(L) HeLa cells from (H) were immunostained with antibodies to CENPF and RAD51. Plots in all cases represent data from three independent experiments; error bars = mean ± SEM, p values: unpaired two-tailed t tests except (E, F, G, and J) (Mann-Whitney). *p $\leq$ 0.05, **p $\leq$ 0.01, and ***p $\leq$ 0.001.

See also Figure S4.

CellPress
OPEN ACCESS

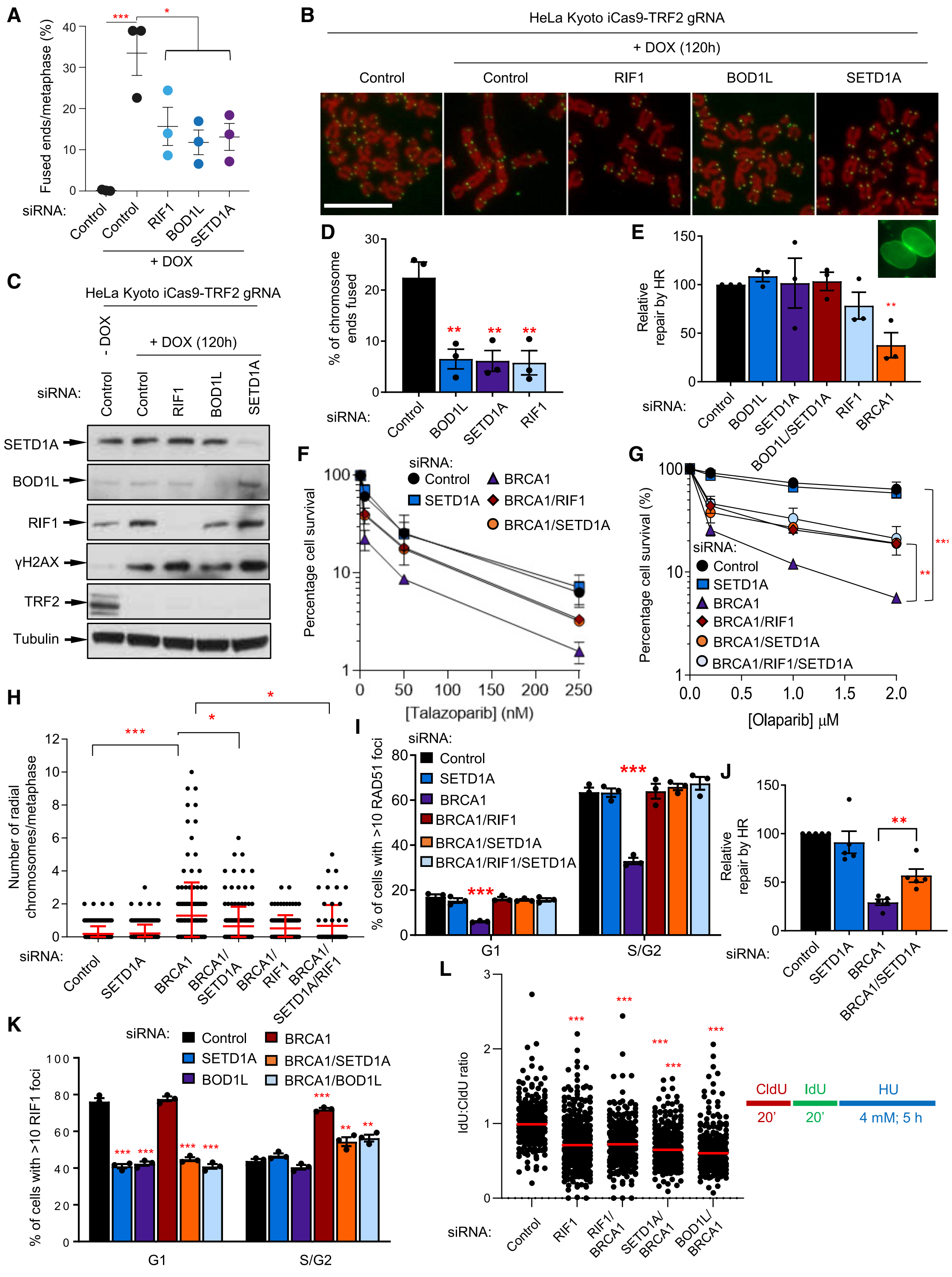

Figure 4. BOD1L, SETD1A, and RIF1 act together to promote NHEJ

(A–C) HeLa Kyoto iCas9-TRF2 gRNA cells were transfected with the indicated siRNAs, treated with doxycycline, and the percentage of telomere end-to-end fusions enumerated (A). Representative images of telomere fusions are shown in (B) (scale bars, 10 μm), and WCE were immunoblotted with the indicated antibodies (C).

(D) HeLa cells were transfected with the indicated siRNAs and a plasmid expressing dominant-negative TRF2$^{\Delta B \Delta M}$, and the percentage of telomere end-to-end fusions enumerated.

(legend continued on next page)

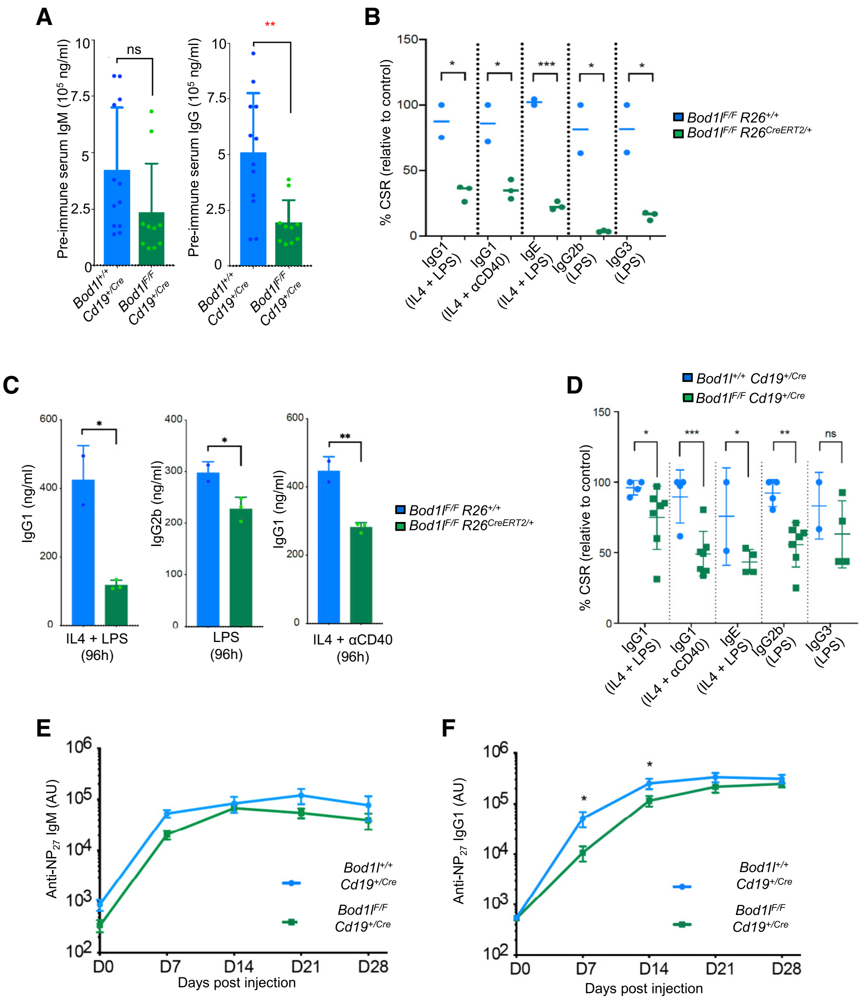

Figure 5. BOD1L is required for CSR

(A) Serum from *Bod1l*$^{+/+}$*Cd19*$^{+/Cre}$ and *Bod1l*$^{F/F}$*Cd19*$^{+/Cre}$ mice was isolated and immunoglobulins quantified by ELISA.

(B and C) CD19^{+} B cells were isolated from *Bod1l*$^{F/F}$*R26*$^{+/+}$ or *Bod1l*$^{F/F}$*R26*$^{CreERT2/+}$ mice and stimulated *in vitro* with the indicated factors for 96 h. Relative levels of CSR were quantified (B), and the quantity of immunoglobulins produced measured by ELISA (C).

(D) CD19^{+} B cells were isolated from *Bod1l*$^{+/+}$ *Cd19*$^{+/Cre}$ and *Bod1l*$^{F/F}$*Cd19*$^{+/Cre}$ mice and stimulated *in vitro* with the indicated factors for 96 h. Relative levels of CSR were quantified.

(E and F) Mice were immunized with NP-CGG, and NP-specific IgM or IgG were quantified in serum at the indicated time points after immunization. Plots in all cases represent data from n = 3 mice; error bars = mean ± SEM, p values: unpaired two-tailed t tests. *p ≤ 0.05, **p ≤ 0.01, and ***p ≤ 0.001. See also Figure S5.

H3K4 methylation on damaged chromatin is an essential pre-requisite for efficient RIF1 localization.

RIF1 associates with methylated H3K4 *in vitro* and *in vivo*

To examine the association of RIF1, DSBs, and H3K4me3 in detail, we next re-analyzed RIF1 ChIP-seq and BLISS (break labeling *In Situ* and sequencing) datasets from mouse embryonic stem cells (mESCs) (Foti et al., 2016; Yan et al., 2017) combined with data from ENCODE. This revealed that chromatin binding correlated with enrichment of H3K4me3 and coincided with endogenous DSBs detected by BLISS (Figures 7A–7C and S7A–S7C). Indeed, the ~5,000 RIF1 binding sites detected in mESCs strongly co-associated with endogenous DSBs (Figure 7C), and ~40 % overlapped with H3K4me3 (Figure S7D). This included regions independent of transcription start sites (Figures S7C–S7F) that are known "hotspots" for H3K4me3 and endogenous DSBs, and also those areas lying outside replication origins identified by

and S6H), which occurred in a BOD1L, SETD1A, and RIF1-dependent fashion (Figure 6O). In agreement, using proximity ligation assays (PLA), association of H3K4me3 with damaged chromatin occurred in a SETD1A-dependent manner (Figure S6I). Finally, RIF1 chromatin binding to the undamaged Lac-operator was also partially reliant on BOD1L/SETD1A (Figure S6J), in keeping with pre-existing SETD1A-dependent H3K4me3 at these sites. In concert, these data establish that

(E) HeLa cells were transfected with the indicated siRNAs and with CRISPR-Cas9 HR plasmids. Cells undergoing HR expressing fluorescent nuclear lamin A/C were quantified.

(F and G) HeLa cells were transfected with the indicated siRNAs, exposed to the indicated doses of Talazoparib or olaparib, left to form colonies for 14 days, stained with methylene blue and colonies counted.

(H and I) Cells from (G) were treated as above, incubated with olaparib for 24 h, and radial chromosome formation analyzed by Giemsa staining and light microscopy (H). Alternatively, cells were immunostained with antibodies to CENPF and RAD51, and foci formation enumerated (I).

(J) HeLa cells were transfected with the indicated siRNAs, and with CRISPR-Cas9 HR plasmids as in (E). Relative levels of HR were enumerated.

(K) HeLa cells were transfected with the indicated siRNAs, exposed to IR, immunostained with antibodies to CENPF and RIF1, and foci formation quantified.

(L) U-2-OS cells were transfected with the indicated siRNAs for 72 h, pulsed for 20 min each with CldU and IdU, and exposed to 4 mM HU for 5 h (as in the schematic). DNA was visualized with antibodies to CldU and IdU, and tract length was calculated. Graph denotes average ratios of IdU:CldU label length. Plots in all cases represent data from three independent experiments; error bars = mean ± SEM, p values: one-way (A) and two-way (F and G) ANOVA; unpaired two-tailed t tests (D, E, I, J, and K); Mann-Whitney (H and L). *p ≤ 0.05, **p ≤ 0.01 and ***p ≤ 0.001.

CellPress
OPEN ACCESS

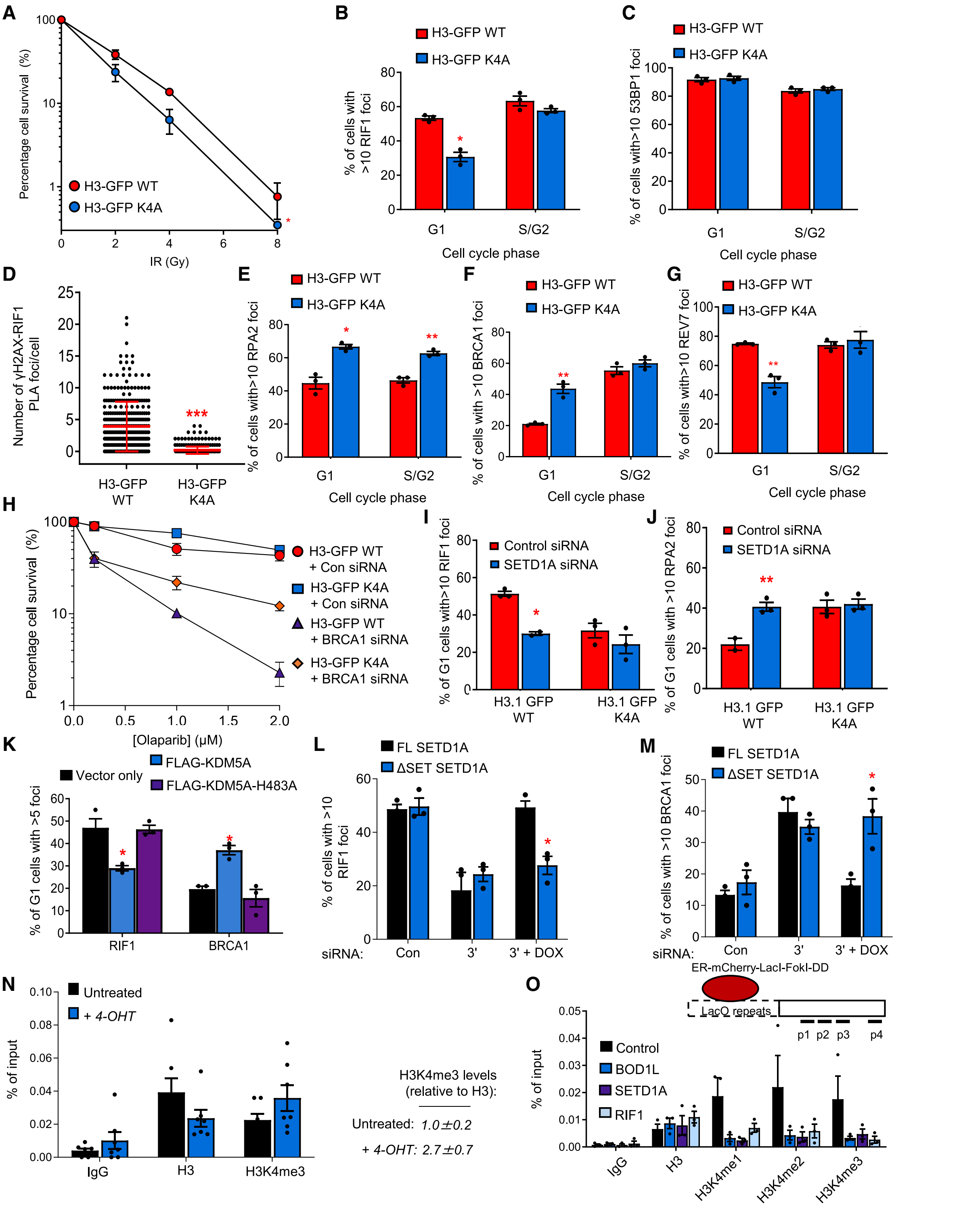

Figure 6. H3K4 methylation by SETD1A is required for RIF1-dependent DNA repair

(A) H3-GFP WT and K4A cells were exposed to ionizing radiation (IR), left to form colonies for 10 days, stained with methylene blue and colonies counted.

(B and C) Cells from (A) were immunostained with antibodies to CENPF and RIF1 (B) or CENPF and 53BP1 (C), and foci formation enumerated.

(D) Quantification of proximity ligation assay (PLA) signals between γH2AX and RIF1 in H3-GFP WT and K4A cells.

(E–G) Cells from (A) were immunostained with antibodies to CENPF and RPA2 (E), CENPF and BRCA1 (F), or CENPF and REV7 (G) and foci formation assessed.

(H) H3-GFP WT and K4A cells were transfected with the indicated siRNAs, exposed to olaparib, left to form colonies for 10 days, stained with methylene blue and colonies counted.

(legend continued on next page)

Okazaki fragment sequencing (OK-seq) (Petryk et al., 2018; Figure S7G).

To probe the underlying mechanisms, we investigated the possibility that RIF1 might interact with methylated histones. *In vitro* pull-down assays of irradiated nuclear extracts revealed that RIF1 bound to recombinant histone H3 methylated at Lys4, but not to unmethylated H3 (Figure 7D), in a manner similar to PHF8, a known reader of H3K4 methylation (Feng et al., 2010). Moreover, recombinant RIF1 and H3K4me3 bound in the absence of other factors, suggesting a direct interaction (Figure 7E). Finally, deletion of the N-terminal HEAT repeats (aa 1–976) of RIF1 abrogated binding to H3K4me3, while a sub-region (aa 1–411) of these repeats supported this interaction when expressed in mammalian cells (Figures 7F and 7G). Together, these data support a model whereby RIF1 binds SETD1A-mediated H3K4me3 via its N-terminal HEAT repeats, which cooperates with phospho-53BP1 binding to facilitate its localization/stabilization at DSB sites.

In summary, our data identify that H3K4 methylation catalyzed by BOD1L and SETD1A counteracts end-resection at DSBs in G1-phase by facilitating RIF1 recruitment (Figure 7H). This protects these breaks from BRCA1-dependent end-resection and promotes repair by NHEJ. In the absence of SETD1A or BOD1L, or when histone methylation is perturbed, RIF1 localization to G1 DSBs is attenuated, NHEJ is severely compromised, and breaks undergo deleterious resection, giving rise to genome instability and hypersensitivity to IR.

DISCUSSION

With the recent identification of shieldin and characterization of RIF1-53BP1 binding, substantial progress has been made in elucidating how the 53BP1 end protection pathway functions. However, there is still significant debate surrounding how precisely these proteins promote end protection, and how this pathway is regulated. Here, we have established that the BOD1L-SETD1A complex plays an important role in recruiting RIF1 to DSBs, with a subsequent impact on REV7-shieldin and end-joining. Critically, we have shown that methylation of H3K4 by SETD1A is vital for RIF1 accumulation on damaged chromatin in G1. Consequently, loss of COMPASS subunits or deregulation of H3K4 methylation leads to inappropriate end-resection, perturbs NHEJ, and has a deleterious impact on CSR. These findings demonstrate that H3K4 methylation plays a direct role in regulating DSB repair.

Roles for the BOD1L, SETD1A, and RIF1 complex

Although BOD1L and SETD1A suppress degradation of stalled/reversed replication forks (Higgs et al., 2015, 2018), their roles in other DNA repair pathways have hitherto not been investigated. Here, we demonstrate that BOD1L and SETD1A functionally interact with RIF1 and its downstream partner REV7 in an intricate interdependent fashion. Indeed, depletion of BOD1L or RIF1 abrogates localization of SETD1A to damaged chromatin, while loss of SETD1A or BOD1L prevents RIF1 recruitment (Figures 1, 2, S1, and S2). These findings are consistent with two scenarios: (1) that these proteins exist in a tripartite complex prior to DSB formation or (2) that they are independently recruited to damaged chromatin and form a complex thereafter. In support of the former hypothesis, our data suggest that BOD1L, SETD1A, and RIF1 interact in unperturbed cells, which is not enhanced by treatment with genotoxic agents. Furthermore, the identification of BOD1L as a potential substrate for ATM/ATR-mediated phosphorylation (Matsuoka et al., 2007) suggests that it may act as a damage-regulatable protein to guide the SETD1A-COMPASS complex to sites of damage and catalyze H3K4 methylation. We therefore speculate that RIF1 is recruited to DSBs via multiple cooperative interactions with methylated H3K4, SETD1A-BOD1L, and phospho-53BP1 (Setiaputra et al., 2022).

SETD1A, BOD1L, and RIF1 also share a role in protecting stalled replication forks from degradation (Garzón et al., 2019; Higgs et al., 2015, 2018; Mukherjee et al., 2019). While we have yet to investigate whether these three factors act in concert in this pathway, it is tempting to speculate that this might be the case. Indeed, BOD1L, SETD1A, and RIF1 all protect replication forks against the helicase/nuclease DNA2, and BOD1L-SETD1A recruit RIF1 to stalled forks (Figure S2). It is important to note that this protective function of RIF1 depends on its ability to interact with the protein phosphatase PP1 (Garzón et al., 2019). However, there is some controversy as to whether PP1 is required for DSB repair: although the PP1 interaction motifs of RIF1 are dispensable for its recruitment to DSBs (Escribano-Díaz et al., 2013), PP1-RIF1 suppresses end-resection (Isobe et al., 2021). Therefore, it may be that the shared phenotypes in cells lacking these factors actually result from disparate mechanisms.

Mechanisms for epigenetic PTMs in regulating the 53BP1 pathway

Our findings further reinforce a model established by studies on H4K20me, H4K16Ac, and H2AK15Ub (Botuyan et al., 2006; Fradet-Turcotte et al., 2013; Nakamura et al., 2019; Tang et al., 2013; Wilson et al., 2016), in which pre-existing and damage-inducible chromatin PTMs directly regulate DSB repair pathways. Our data also imply that H3K4 methylation may mark G1 DSBs for end protection. This is seemingly at odds with previous

(I and J) H3-GFP WT and K4A cells were transfected with the indicated siRNA, exposed to IR, and immunostained with antibodies to CENPF and RIF1 (I), or CENPF and RPA (J), and foci formation enumerated.
(K) HeLa cells were transfected with constructs expressing WT or H483A KDM5A, and immunostained with antibodies to either CENPF and RIF1 or CENPF and BRCA1. Representative images are shown in Figure S6G.
(L and M) U-2-OS cell lines bearing inducible full-length (FL) SETD1A or a variant lacking the SET (ΔSET) domain were transfected with the indicated siRNAs, exposed to doxycycline where denoted, and exposed to IR. RIF1 or BRCA1 foci formation was then quantified as above.
(N and O) U-2-OS-FokI cells were treated with 4-OHT and/or transfected with the indicated siRNAs, chromatin isolated and ChIP was performed with the indicated antibodies. Data represent the average signal across the 4 amplicons represented in the schematic normalized to input. Plots in all cases represent data from at least three independent experiments; error bars = mean ± SEM, p values: unpaired two-tailed t tests except (A) (two-way ANOVA) and (D) (Mann-Whitney). *$p \leq 0.05$, **$p \leq 0.01$, and ***$p \leq 0.001$.
See also Figure S6.

CellPress
OPEN ACCESS

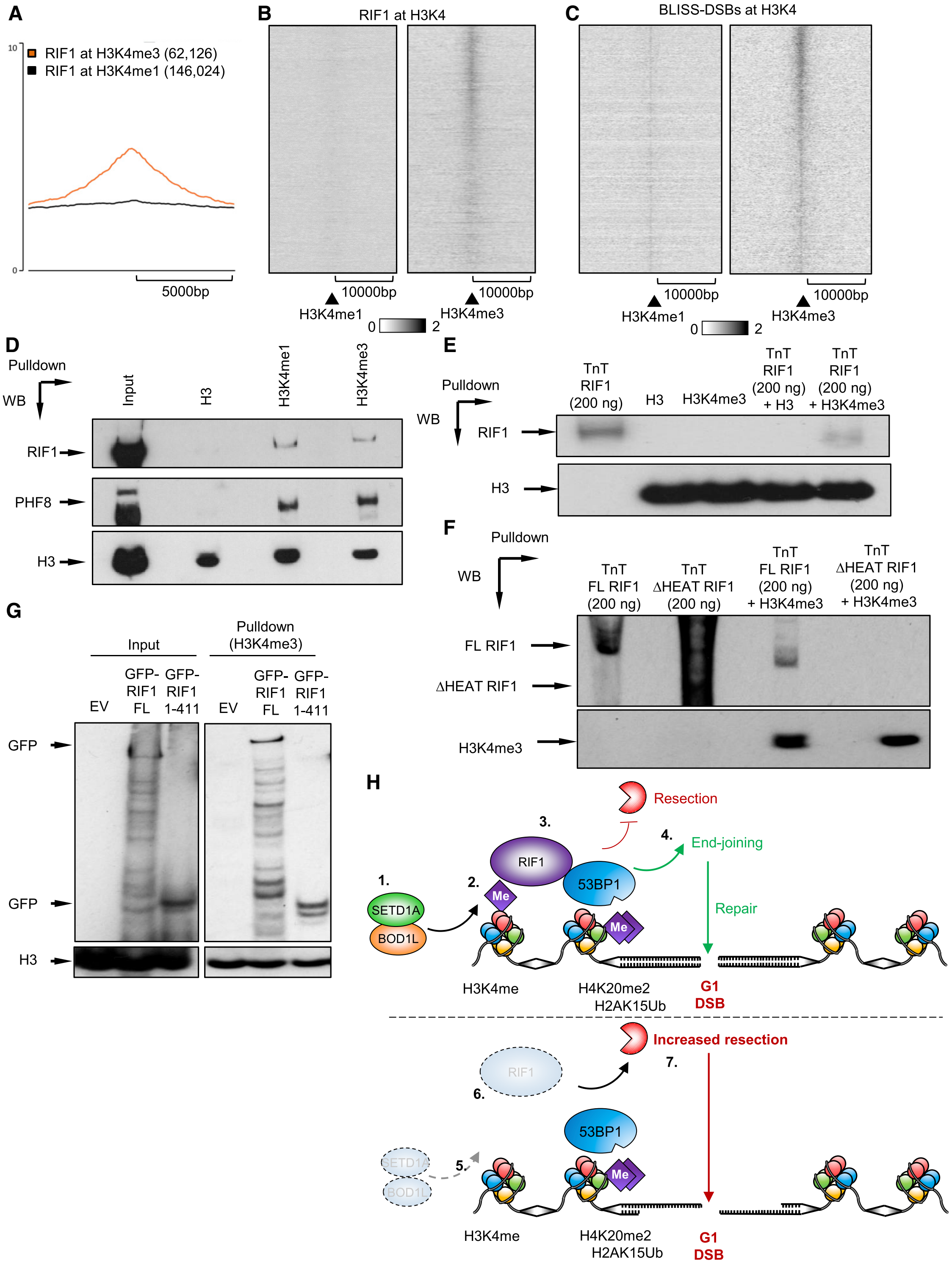

Figure 7. RIF1 associates with methylated H3K4 *in vitro* and *in vivo*

(A) Chromatin immunoprecipitation profiles of murine RIF11 at H3K4me1 and H3K4me3 peak sites in mESCs from ENCODE. Data are from Foti et al. (2016).

(B and C) Chromatin immunoprecipitation profiles of murine RIF1 and BLISS signals over H3K4me3-positive areas in mESCs that lie outside areas defined as TSS by ENCODE. Data are from Yan et al. (2017).

(D) HeLa nuclear cell extracts were incubated with biotinylated histones and analyzed by immunoblotting.

(E and F) *In vitro* transcribed/translated RIF1 was incubated with biotinylated histones and analyzed by immunoblotting.

(legend continued on next page)

conclusions that DSBs occurring within transcriptionally active genes are preferentially repaired by HR (Clouaire et al., 2018), and with other data suggesting a role for lysine demethylation in DSB repair (Bayo et al., 2018; Li et al., 2014; Mosammaparast et al., 2013), especially at *FokI*-induced DSBs (Gong et al., 2017). In contrast, other studies provide evidence that levels of H3K4me3 are either unchanged (Moyal et al., 2011) or increased (Faucher and Wellinger, 2010; Nakamura et al., 2011) at DSBs induced by microirradiation or the *I-SceI* and *HO* nucleases.

While these discrepancies are seemingly difficult to reconcile, our demonstration that RIF1 binds methylated histones *in vitro*, combined with our genome-wide analyses and ChIP and PLA data, convincingly demonstrates that H3K4me3 directly regulates RIF1 recruitment. Although the precise regions of RIF1 that bind methylated H3K4 remain to be determined, our data (Figure 7) suggest that the N-terminal HEAT repeats of RIF1 mediate this interaction. Although HEAT repeats are not considered canonical methyl-binding domains, recent studies of condensin suggest that other HEAT-repeat containing proteins also bind methylated H3K4 (Yuen et al., 2017). This is indirectly supported by data demonstrating that the N-terminal HEAT repeats of RIF1 are indispensable for its recruitment to DSBs (Escribano-Díaz et al., 2013), and that they mediate interactions with phospho-53BP1 and the shieldin complex (Setiaputra et al., 2022). These findings force us to re-evaluate the role of chromatin modifications in controlling the 53BP1-RIF1-shieldin pathway. We hypothesize that RIF1, similar to 53BP1 (Fradet-Turcotte et al., 2013), reads the epigenetic status of chromatin surrounding a DSB to control repair. Characterizing the precise nature and timing of RIF1 recruitment to H3K4 methylation remains an important avenue for further investigation.

Clinical implications of perturbing the RIF1-SETD1A-BOD1L axis

From a clinical perspective, this study reveals that loss of SETD1A or BOD1L confers PARPi resistance in *BRCA1*-deficient cells by restoring HR, in a manner similar to loss of 53BP1, RIF1, or components of the shieldin complex (Chapman et al., 2013; Dev et al., 2018; Ghezraoui et al., 2018; Noordermeer et al., 2018). Therefore, compromising the activity of the COMPASS complex may represent a potential mechanism by which *BRCA1*-mutant tumors become resistant to PARPi. However, it remains to be determined whether this is a bona fide pathological mechanism of resistance in patients. It is intriguing that while loss of RIF1 or SETD1A gives rise to PARPi resistance in the absence of BRCA1, it does not rescue the excessive fork degradation seen in *BRCA1*-deficient cells (Figure 4). Since this represents an alternative mechanism of PARPi resistance (Ray Chaudhuri et al., 2016), these findings suggest that restoration of HR can override loss of fork protection and that even in cells lacking the same genetic factors, mechanisms of resistance are likely multi-factorial.

In addition, cell lines from SETD1A haploinsufficient patients also exhibit defects in RIF1 recruitment to DSBs (Figures 2 and 3). SETD1A haploinsufficiency is associated with a range of neuropsychiatric conditions including schizophrenia, epilepsy with seizures, obsessive compulsive disorder, psychotic episodes, and intellectual disability (Kummeling et al., 2021; Nagahama et al., 2020; Singh et al., 2016; Yu et al., 2019). While it is unclear how allelic imbalance of SETD1A causes these symptoms, they are likely due to a combination of altered chromatin state and transcriptional changes (Cameron et al., 2019), affecting neuronal fitness and inter-neuronal communication. Although it is plausible that SETD1A (and BOD1L) might contribute to neuronal fitness by promoting DSB repair, these patients show no overt defects in immunoglobulin class-switching. Therefore, further work is needed to determine whether haploinsufficient mutations in SETD1A reduce the DSB repair capacity of neuronal cells.

Limitations of the study

Our work supports a role for H3K4 methylation in recruiting RIF1 to DSBs. However, our ability to distinguish between *de novo* and pre-existing histone modifications at DSBs is limited. In part, this is due to the systems used, which are reliant on enzyme-induced DSBs induced in highly repetitive genomic regions, probably concentrated in open chromatin. Furthermore, although techniques such as ChIP are useful to understand broad changes in chromatin at specific sites, they do not provide detailed temporal or positional information to comprehend the dynamics of RIF1 recruitment and stabilization. Precisely how SETD1A, BOD1L, H3K4, and RIF1 cooperate during DSB repair therefore remains an open question.

Lastly, although perturbation of H3K4 methylation affects RIF1 localization in multiple systems (Figures 6 and 7), we cannot exclude the possibility that this may involve indirect perturbation of other cellular pathways. Indeed, since H3K4me3 is linked with active transcription, it is entirely plausible that DSB-associated transcription helps mediate RIF1 localization. Moreover, delineating the importance of mono-, di-, and tri-methylation of H3K4me is not trivial, as seen in *in vitro* and *in vivo* data from Figure 7. Elucidating the mechanisms by which SETD1A, BOD1L, and RIF1 are recruited to DSBs in the future will require techniques such as super-resolution microscopy.

STAR★METHODS

Detailed methods are provided in the online version of this paper and include the following:

- KEY RESOURCES TABLE
- RESOURCE AVAILABILITY
  - Lead contact
  - Materials availability

(G) HEK-293 cells were transfected with plasmids expressing the indicated RIF1 proteins, left for 48 h, and cell lysates incubated with purified biotinylated histones and analyzed by immunoblotting. Data in (D)–(G) represent ≥ 2 independent experiments.

(H) (Upper) Upon DSB formation in G1, pre-existing H4K20me2 and H2AK15Ub recruit 53BP1 to sites of DSBs. H3K4me catalyzed by SETD1A and its co-factor BOD1L (1 and 2) stabilize the recruitment of RIF1 (3), allowing downstream cascades (not shown). This suppresses BRCA1-dependent resection and promotes NHEJ (4). (Lower) In the absence of H3K4me (5), RIF1 recruitment is destabilized (6), leading to inappropriate BRCA1-dependent resection of DSBs (7) and mis-repair. See also Figure S7.

CellPress
OPEN ACCESS

- ○ Data and code availability
- ● EXPERIMENTAL MODEL AND SUBJECT DETAILS
  - ○ Cell lines and culture
  - ○ Mouse models
  - ○ Plasmids and cloning
- ● METHOD DETAILS
  - ○ Transfections
  - ○ ELISA
  - ○ Antibodies
  - ○ Clonogenic survival assays
  - ○ Immunofluorescence, microscopy and image analysis
  - ○ DNA fibre analysis
  - ○ Proximity ligation assay
  - ○ Chromatin immunoprecipitation
  - ○ Western blotting, pull-downs and immunoprecipitation
  - ○ Metaphase spreads and telomere FISH
  - ○ Class switch recombination assays
  - ○ Homologous recombination assay
  - ○ Mass spectrometry and proteomics
- ● QUANTIFICATION AND STATISTICAL ANALYSIS
  - ○ Data analysis of H3K4/RIF1 binding
  - ○ Statistical analysis

SUPPLEMENTAL INFORMATION

Supplemental information can be found online at https://doi.org/10.1016/j.molcel.2022.03.030.

ACKNOWLEDGMENTS

We thank Clare Davies, Jo Morris, and other members of the Birmingham Centre for Genome Biology (BCGB) for invaluable discussions. We are grateful to Robert Klose for providing KDM5A expression reagents and Graham Dellaire for providing plasmids for the CRISPR-based HR assay. We thank the Crick BRF and GEMs for support with animal production and experiments. R.B. was funded by a Birmingham Fellowship and a Wellcome Institutional Strategic Support Fund (204846/Z/16/Z) awarded to M.R.H. by the University of Birmingham. E.S. is funded by the University of Birmingham and supported by Cancer Research UK (C17422/A25154). R.M.A.M. was funded by the UK Medical Research Council and University of Birmingham. M.R.H., A.O., and A.G. are funded by a UK Medical Research Council Career Development Fellowship (MR/P009085/1). M.S. is supported by a Birmingham Fellowship, the Wellcome Trust (202115/Z/16/Z), Royal Society (RG170246), and BBSRC (BB/S016155/1). G.S.S. is funded by a CR-UK Programme Grant (C17183/A23303). S.J.B., P.R., T.S., and V.B. are supported by the Francis Crick Institute, which receives its core funding from Cancer Research UK (FC0010048), the UK Medical Research Council (FC0010048), and the Wellcome Trust (FC0010048). S.J.B. is also funded by a European Research Council (ERC) Advanced Investigator Grant (TelMetab) and Wellcome Trust Senior Investigator and Collaborative Grants.

AUTHOR CONTRIBUTIONS

Conceptualization, M.R.H., G.S.S., and S.J.B.; investigation, R.B., V.B., R.J.M., E.S., P.R., A.O., A.G., R.M.A.M., M.S., G.S.S., and M.R.H.; resources, T.S. and G.H.; writing—original draft, R.B., V.B., S.J.B., and M.R.H. with comments from all authors; writing—review & editing, M.R.H., S.J.B., and R.B.; funding acquisition, M.R.H., G.S.S., and S.J.B.; supervision, M.R.H., G.S.S., and S.J.B.

DECLARATION OF INTERESTS

S.J.B. is a co-founder and VP in Science Strategy at Artios Pharma and is a member of the *Molecular Cell* advisory board. The authors declare no other competing interests.

Received: May 10, 2021
Revised: December 14, 2021
Accepted: March 23, 2022
Published: April 18, 2022

REFERENCES

Afgan, E., Baker, D., Batut, B., van den Beek, M., Bouvier, D., Cech, M., Chilton, J., Clements, D., Coraor, N., Grüning, B.A., et al. (2018). The Galaxy platform for accessible, reproducible and collaborative biomedical analyses: 2018 update. Nucleic Acids Res. *46*, W537–W544.

Bayo, J., Tran, T.A., Wang, L., Peña-Llopis, S., Das, A.K., and Martinez, E.D. (2018). Jumonji inhibitors overcome radioresistance in cancer through changes in H3K4 methylation at double-strand breaks. Cell Rep. *25*, 1040–1050.e5.

Biehs, R., Steinlage, M., Barton, O., Juhász, S., Künzel, J., Spies, J., Shibata, A., Jeggo, P.A., and Löbrich, M. (2017). DNA double-strand break resection occurs during non-homologous end joining in G1 but is distinct from resection during homologous recombination. Mol. Cell *65*, 671–684.e5.

Boersma, V., Moatti, N., Segura-Bayona, S., Peuscher, M.H., van der Torre, J., Wevers, B.A., Orthwein, A., Durocher, D., and Jacobs, J.J.L. (2015). MAD2L2 controls DNA repair at telomeres and DNA breaks by inhibiting 5′ end resection. Nature *521*, 537–540.

Bothmer, A., Robbiani, D.F., Feldhahn, N., Gazumyan, A., Nussenzweig, A., and Nussenzweig, M.C. (2010). 53BP1 regulates DNA resection and the choice between classical and alternative end joining during class switch recombination. J. Exp. Med. *207*, 855–865.

Botuyan, M.V., Lee, J., Ward, I.M., Kim, J.E., Thompson, J.R., Chen, J., and Mer, G. (2006). Structural basis for the methylation state-specific recognition of histone H4-K20 by 53BP1 and Crb2 in DNA repair. Cell *127*, 1361–1373.

Bunting, S.F., Callén, E., Wong, N., Chen, H.T., Polato, F., Gunn, A., Bothmer, A., Feldhahn, N., Fernandez-Capetillo, O., Cao, L., et al. (2010). 53BP1 inhibits homologous recombination in BRCA1-deficient cells by blocking resection of DNA breaks. Cell *141*, 243–254.

Callen, E., Zong, D., Wu, W., Wong, N., Stanlie, A., Ishikawa, M., Pavani, R., Dumitrache, L.C., Byrum, A.K., Mendez-Dorantes, C., et al. (2020). 53BP1 enforces distinct pre- and post-resection blocks on homologous recombination. Mol. Cell *77*, 26–38.e7.

Cameron, D., Blake, D.J., Bray, N.J., and Hill, M.J. (2019). Transcriptional changes following cellular knockdown of the Schizophrenia risk gene SETD1A are enriched for common variant association with the disorder. Mol. Neuropsychiatr. *5*, 109–114.

Chapman, J.R., Barral, P., Vannier, J.B., Borel, V., Steger, M., Tomas-Loba, A., Sartori, A.A., Adams, I.R., Batista, F.D., and Boulton, S.J. (2013). RIF1 is essential for 53BP1-dependent nonhomologous end joining and suppression of DNA double-strand break resection. Mol. Cell *49*, 858–871.

Clouaire, T., Rocher, V., Lashgari, A., Arnould, C., Aguirrebengoa, M., Biernacka, A., Skrzypczak, M., Aymard, F., Fongang, B., Dojer, N., et al. (2018). Comprehensive mapping of histone modifications at DNA double-strand breaks deciphers repair pathway chromatin signatures. Mol. Cell *72*, 250–262.e6.

Cox, J., and Mann, M. (2008). MaxQuant enables high peptide identification rates, individualized p.p.b.-range mass accuracies and proteome-wide protein quantification. Nat. Biotechnol. *26*, 1367–1372.

Daniel, J.A., Santos, M.A., Wang, Z., Zang, C., Schwab, K.R., Jankovic, M., Filsuf, D., Chen, H.T., Gazumyan, A., Yamane, A., et al. (2010). PTIP promotes chromatin changes critical for immunoglobulin class switch recombination. Science *329*, 917–923.

Dev, H., Chiang, T.W., Lescale, C., de Krijger, I., Martin, A.G., Pilger, D., Coates, J., Sczaniecka-Clift, M., Wei, W., Ostermaier, M., et al. (2018). Shieldin complex promotes DNA end-joining and counters homologous recombination in BRCA1-null cells. Nat. Cell Biol. *20*, 954–965.

Di Virgilio, M., Callen, E., Yamane, A., Zhang, W., Jankovic, M., Gitlin, A.D., Feldhahn, N., Resch, W., Oliveira, T.Y., Chait, B.T., et al. (2013). Rif1 prevents

resection of DNA breaks and promotes immunoglobulin class switching. Science *339*, 711–715.

Difilippantonio, S., Gapud, E., Wong, N., Huang, C.Y., Mahowald, G., Chen, H.T., Kruhlak, M.J., Callen, E., Livak, F., Nussenzweig, M.C., et al. (2008). 53BP1 facilitates long-range DNA end-joining during V(D)J recombination. Nature *456*, 529–533.

Escribano-Díaz, C., Orthwein, A., Fradet-Turcotte, A., Xing, M., Young, J.T., Tkáč, J., Cook, M.A., Rosebrock, A.P., Munro, M., Canny, M.D., et al. (2013). A cell cycle-dependent regulatory circuit composed of 53BP1-RIF1 and BRCA1-CtIP controls DNA repair pathway choice. Mol. Cell *49*, 872–883.

Faucher, D., and Wellinger, R.J. (2010). Methylated H3K4, a transcription-associated histone modification, is involved in the DNA damage response pathway. PLoS Genet. *6*, e1001082.

Feng, W., Yonezawa, M., Ye, J., Jenuwein, T., and Grummt, I. (2010). PHF8 activates transcription of rRNA genes through H3K4me3 binding and H3K9me1/2 demethylation. Nat. Struct. Mol. Biol. *17*, 445–450.

Ferrand, J., Plessier, A., and Polo, S.E. (2021). Control of the chromatin response to DNA damage: histone proteins pull the strings. Semin. Cell Dev. Biol. *113*, 75–87.

Foti, R., Gnan, S., Cornacchia, D., Dileep, V., Bulut-Karslioglu, A., Diehl, S., Buness, A., Klein, F.A., Huber, W., Johnstone, E., et al. (2016). Nuclear architecture organized by Rif1 underpins the replication-timing program. Mol. Cell *61*, 260–273.

Fradet-Turcotte, A., Canny, M.D., Escribano-Díaz, C., Orthwein, A., Leung, C.C., Huang, H., Landry, M.C., Kitevski-LeBlanc, J., Noordermeer, S.M., Sicheri, F., and Durocher, D. (2013). 53BP1 is a reader of the DNA-damage-induced H2A Lys 15 ubiquitin mark. Nature *499*, 50–54.

Garzón, J., Ursich, S., Lopes, M., Hiraga, S.I., and Donaldson, A.D. (2019). Human RIF1-protein phosphatase 1 prevents degradation and breakage of nascent DNA on replication stalling. Cell Rep. *27*, 2558–2566.e4.

Ghezraoui, H., Oliveira, C., Becker, J.R., Bilham, K., Moralli, D., Anzilotti, C., Fischer, R., Deobagkar-Lele, M., Sanchiz-Calvo, M., Fueyo-Marcos, E., et al. (2018). 53BP1 cooperation with the REV7-shieldin complex underpins DNA structure-specific NHEJ. Nature *560*, 122–127.

Gong, F., Clouaire, T., Aguirrebengoa, M., Legube, G., and Miller, K.M. (2017). Histone demethylase KDM5A regulates the ZMYND8-NuRD chromatin remodeler to promote DNA repair. J. Cell Biol. *216*, 1959–1974.

Hewitt, G., Borel, V., Segura-Bayona, S., Takaki, T., Ruis, P., Bellelli, R., Lehmann, L.C., Sommerova, L., Vancevska, A., Tomas-Loba, A., et al. (2021). Defective ALC1 nucleosome remodeling confers PARPi sensitization and synthetic lethality with HRD. Mol. Cell *81*, 767–783.e11.

Higgs, M.R., Reynolds, J.J., Winczura, A., Blackford, A.N., Borel, V., Miller, E.S., Zlatanou, A., Nieminuszczy, J., Ryan, E.L., Davies, N.J., et al. (2015). BOD1L is required to suppress deleterious resection of stressed replication forks. Mol. Cell *59*, 462–477.

Higgs, M.R., Sato, K., Reynolds, J.J., Begum, S., Bayley, R., Goula, A., Vernet, A., Paquin, K.L., Skalnik, D.G., Kobayashi, W., et al. (2018). Histone methylation by SETD1A protects nascent DNA through the nucleosome chaperone activity of FANCD2. Mol. Cell *71*, 25–41.e6.

Hoshii, T., Cifani, P., Feng, Z., Huang, C.H., Koche, R., Chen, C.W., Delaney, C.D., Lowe, S.W., Kentsis, A., and Armstrong, S.A. (2018). A non-catalytic function of SETD1A regulates cyclin K and the DNA damage response. Cell *172*, 1007–1021.e17.

Isobe, S.Y., Hiraga, S.I., Nagao, K., Sasanuma, H., Donaldson, A.D., and Obuse, C. (2021). Protein phosphatase 1 acts as a RIF1 effector to suppress DSB resection prior to Shieldin action. Cell Rep. *36*, 109383.

Klose, R.J., Yan, Q., Tothova, Z., Yamane, K., Erdjument-Bromage, H., Tempst, P., Gilliland, D.G., Zhang, Y., and Kaelin, W.G., Jr. (2007). The retinoblastoma binding protein RBP2 is an H3K4 demethylase. Cell *128*, 889–900.

Kranz, A., and Anastassiadis, K. (2020). The role of SETD1A and SETD1B in development and disease. Biochim. Biophys. Acta Gene Regul. Mech. *1863*, 194578.

Kummeling, J., Stremmelaar, D.E., Raun, N., Reijnders, M.R.F., Willemsen, M.H., Ruiterkamp-Versteeg, M., Schepens, M., Man, C.C.O., Gilissen, C., Cho, M.T., et al. (2021). Characterization of SETD1A haploinsufficiency in humans and *Drosophila* defines a novel neurodevelopmental syndrome. Mol. Psychiatry *26*, 2013–2024.

Li, X., Liu, L., Yang, S., Song, N., Zhou, X., Gao, J., Yu, N., Shan, L., Wang, Q., Liang, J., et al. (2014). Histone demethylase KDM5B is a key regulator of genome stability. Proc. Natl. Acad. Sci. USA *111*, 7096–7101.

Li, X., and Tyler, J.K. (2016). Nucleosome disassembly during human non-homologous end joining followed by concerted HIRA- and CAF-1-dependent reassembly. Elife *5*, e15129.

Matsuoka, S., Ballif, B.A., Smogorzewska, A., McDonald, E.R., 3rd, Hurov, K.E., Luo, J., Bakalarski, C.E., Zhao, Z., Solimini, N., Lerenthal, Y., et al. (2007). ATM and ATR substrate analysis reveals extensive protein networks responsive to DNA damage. Science *316*, 1160–1166.

McNee, G., Eales, K.L., Wei, W., Williams, D.S., Barkhuizen, A., Bartlett, D.B., Essex, S., Anandram, S., Filer, A., Moss, P.A., et al. (2017). Citrullination of histone H3 drives IL-6 production by bone marrow mesenchymal stem cells in MGUS and multiple myeloma. Leukemia *31*, 373–381.

Mosammaparast, N., Kim, H., Laurent, B., Zhao, Y., Lim, H.J., Majid, M.C., Dango, S., Luo, Y., Hempel, K., Sowa, M.E., et al. (2013). The histone demethylase LSD1/KDM1A promotes the DNA damage response. J. Cell Biol. *203*, 457–470.

Moyal, L., Lerenthal, Y., Gana-Weisz, M., Mass, G., So, S., Wang, S.Y., Eppink, B., Chung, Y.M., Shalev, G., Shema, E., et al. (2011). Requirement of ATM-dependent monoubiquitylation of histone H2B for timely repair of DNA double-strand breaks. Mol. Cell *41*, 529–542.

Mukherjee, C., Tripathi, V., Manolika, E.M., Heijink, A.M., Ricci, G., Merzouk, S., de Boer, H.R., Demmers, J., van Vugt, M.A.T.M., and Ray Chaudhuri, A. (2019). RIF1 promotes replication fork protection and efficient restart to maintain genome stability. Nat. Commun. *10*, 3287.

Nagahama, K., Sakoori, K., Watanabe, T., Kishi, Y., Kawaji, K., Koebis, M., Nakao, K., Gotoh, Y., Aiba, A., Uesaka, N., and Kano, M. (2020). Setd1a insufficiency in mice attenuates excitatory synaptic function and recapitulates schizophrenia-related behavioral abnormalities. Cell Rep. *32*, 108126.

Nakamura, K., Kato, A., Kobayashi, J., Yanagihara, H., Sakamoto, S., Oliveira, D.V., Shimada, M., Tauchi, H., Suzuki, H., Tashiro, S., et al. (2011). Regulation of homologous recombination by RNF20-dependent H2B ubiquitination. Mol. Cell *41*, 515–528, H2B.

Nakamura, K., Saredi, G., Becker, J.R., Foster, B.M., Nguyen, N.V., Beyer, T.E., Cesa, L.C., Faull, P.A., Lukauskas, S., Frimurer, T., et al. (2019). H4K20me0 recognition by BRCA1-BARD1 directs homologous recombination to sister chromatids. Nat. Cell Biol. *21*, 311–318.

Noordermeer, S.M., Adam, S., Setiaputra, D., Barazas, M., Pettitt, S.J., Ling, A.K., Olivieri, M., Álvarez-Quilón, A., Moatti, N., Zimmermann, M., et al. (2018). The shieldin complex mediates 53BP1-dependent DNA repair. Nature *560*, 117–121.

Petermann, E., Orta, M.L., Issaeva, N., Schultz, N., and Helleday, T. (2010). Hydroxyurea-stalled replication forks become progressively inactivated and require two different RAD51-mediated pathways for restart and repair. Mol. Cell *37*, 492–502.

Petryk, N., Dalby, M., Wenger, A., Stromme, C.B., Strandsby, A., Andersson, R., and Groth, A. (2018). MCM2 promotes symmetric inheritance of modified histones during DNA replication. Science *361*, 1389–1392.

Pinder, J., Salsman, J., and Dellaire, G. (2015). Nuclear domain 'knock-in' screen for the evaluation and identification of small molecule enhancers of CRISPR-based genome editing. Nucleic Acids Res. *43*, 9379–9392.

Ray Chaudhuri, A., Callen, E., Ding, X., Gogola, E., Duarte, A.A., Lee, J.E., Wong, N., Lafarga, V., Calvo, J.A., Panzarino, N.J., et al. (2016). Replication fork stability confers chemoresistance in BRCA-deficient cells. Nature *535*, 382–387.

Ruis, P., Van Ly, D., Borel, V., Kafer, G.R., McCarthy, A., Howell, S., Blassberg, R., Snijders, A.P., Briscoe, J., Niakan, K.K., et al. (2021). TRF2-independent chromosome end protection during pluripotency. Nature *589*, 103–109.

CellPress
OPEN ACCESS

Sato, K., Ishiai, M., Toda, K., Furukoshi, S., Osakabe, A., Tachiwana, H., Takizawa, Y., Kagawa, W., Kitao, H., Dohmae, N., et al. (2012). Histone chaperone activity of fanconi anemia proteins, FANCD2 and FANCI, is required for DNA crosslink repair. EMBO J. *31*, 3524–3536.

Setiaputra, D., Escribano-Díaz, C., Reinert, J.K., Sadana, P., Zong, D., Callen, E., Sifri, C., Seebacher, J., Nussenzweig, A., Thomä, N.H., et al. (2022). RIF1 acts in DNA repair through phosphopeptide recognition of 53BP1. Mol. Cell. *82*, 1359–1371.e9.

Shanbhag, N.M., and Greenberg, R.A. (2013). The dynamics of DNA damage repair and transcription. Methods Mol. Biol. *1042*, 227–235.

Singh, T., Kurki, M.I., Curtis, D., Purcell, S.M., Crooks, L., McRae, J., Suvisaari, J., Chheda, H., Blackwood, D., Breen, G., et al. (2016). Rare loss-of-function variants in SETD1A are associated with schizophrenia and developmental disorders. Nat. Neurosci. *19*, 571–577.

Stringer, B.W., Day, B.W., D'Souza, R.C.J., Jamieson, P.R., Ensbey, K.S., Bruce, Z.C., Lim, Y.C., Goasdoué, K., Offenhäuser, C., Akgül, S., et al. (2019). A reference collection of patient-derived cell line and xenograft models of proneural, classical and mesenchymal glioblastoma. Sci. Rep. *9*, 4902.

Tang, J., Cho, N.W., Cui, G., Manion, E.M., Shanbhag, N.M., Botuyan, M.V., Mer, G., and Greenberg, R.A. (2013). Acetylation limits 53BP1 association with damaged chromatin to promote homologous recombination. Nat. Struct. Mol. Biol. *20*, 317–325.

Tusi, B.K., Deng, C., Salz, T., Zeumer, L., Li, Y., So, C.W., Morel, L.M., Qiu, Y., and Huang, S. (2015). Setd1a regulates progenitor B-cell-to-precursor B-cell development through histone H3 lysine 4 trimethylation and Ig heavy-chain rearrangement. FASEB J. *29*, 1505–1515.

Tyanova, S., Temu, T., Sinitcyn, P., Carlson, A., Hein, M.Y., Geiger, T., Mann, M., and Cox, J. (2016). The Perseus computational platform for comprehensive analysis of (prote)omics data. Nat. Methods *13*, 731–740.

Wilson, M.D., Benlekbir, S., Fradet-Turcotte, A., Sherker, A., Julien, J.P., McEwan, A., Noordermeer, S.M., Sicheri, F., Rubinstein, J.L., and Durocher, D. (2016). The structural basis of modified nucleosome recognition by 53BP1. Nature *536*, 100–103.

Xu, G., Chapman, J.R., Brandsma, I., Yuan, J., Mistrik, M., Bouwman, P., Bartkova, J., Gogola, E., Warmerdam, D., Barazas, M., et al. (2015). REV7 counteracts DNA double-strand break resection and affects PARP inhibition. Nature *521*, 541–544.

Yan, W.X., Mirzazadeh, R., Garnerone, S., Scott, D., Schneider, M.W., Kallas, T., Custodio, J., Wernersson, E., Li, Y., Gao, L., et al. (2017). BLISS is a versatile and quantitative method for genome-wide profiling of DNA double-strand breaks. Nat. Commun. *8*, 15058.

Yu, X., Yang, L., Li, J., Li, W., Li, D., Wang, R., Wu, K., Chen, W., Zhang, Y., Qiu, Z., and Zhou, W. (2019). *De novo* and inherited SETD1A variants in early-onset epilepsy. Neurosci. Bull. *35*, 1045–1057.

Yuen, K.C., Slaughter, B.D., and Gerton, J.L. (2017). Condensin II is anchored by TFIIIC and H3K4me3 in the mammalian genome and supports the expression of active dense gene clusters. Sci. Adv. *3*, e1700191.

Zheng, S., Li, D., Lu, Z., Liu, G., Wang, M., Xing, P., Wang, M., Dong, Y., Wang, X., Li, J., and Chen, X. (2018). Bre1-dependent H2B ubiquitination promotes homologous recombination by stimulating histone eviction at DNA breaks. Nucleic Acids Res. *46*, 11326–11339.

Zimmermann, M., Lottersberger, F., Buonomo, S.B., Sfeir, A., and de Lange, T. (2013). 53BP1 regulates DSB repair using Rif1 to control 5′ end resection. Science *339*, 700–704.

STAR★METHODS

KEY RESOURCES TABLE

REAGENT or RESOURCE	SOURCE	IDENTIFIER
Antibodies		
BOD1L	Grant Stewart (Higgs et al., 2015)	N/A
SETD1A	Bethyl	Cat# A300-289A; RRID: AB_263413
RPA	Millipore	Cat# NA18; RRID: AB_10682810
P-RPA (S4/8)	Bethyl	Cat# A300-245A; RRID: AB_210547
RAD51	Millipore	Cat# PC130; RRID: AB_2238184
γH2AX	Millipore	Cat# 05-636; RRID: AB_309864
H2A	Millipore	Cat# 07-146; RRID: AB_11212920
IdU (BrdU)	Becton Dickinson	Cat# 347580; RRID: AB_10015219
H3	Abcam	Cat# ab1791; RRID: AB_302613
H3K4me1	Abcam	Cat# ab8895; RRID: AB_306847
H3K4me2	Millipore	Cat# 04-790; RRID: AB_10562969
H3K4me3	Abcam	Cat# ab8580; RRID: AB_306649
RIF1	Bethyl	Cat# A300-568A; RRID:AB_669806
REV7	Abcam	Cat# ab180579; RRID: AB_2890174
53BP1	Novus	Cat# NB100-904; RRID: AB_10002714
P-53BP1 (S/S)	Simon Boulton	N/A
P-53BP1 (S824)	Simon Boulton	N/A
BRCA1	Santa Cruz	Cat # sc-6954; RRID:AB_626761
PTIP	Millipore	Cat# ABE69; RRID:AB_10807305
CHD4	Cell Signalling	Cat# 11912; RRID:AB_2751014
P-KAP1 (S824)	Abcam	Cat# 70369; RRID:AB_1209417
CHK1	Sigma Aldrich	Cat# C9358; RRID:AB_259159
P-CHK1 (S345)	Cell Signalling	Cat# 2348; RRID:AB_331212
CHK2	Millipore	Cat# 05-649; RRID:AB_2244941
P-RPA (S33)	Bethyl	Cat# A300-246A; RRID:AB_2180847
PCNA	Santa Cruz	Cat# sc-7907; RRID:AB_2160375
CENPF (mouse)	BD	Cat# 610768; RRID: AB_398091

(*Continued on next page*)

CellPress
OPEN ACCESS

Continued

REAGENT or RESOURCE	SOURCE	IDENTIFIER
CENPF (rabbit)	Abcam	Cat# Ab5; RRID: AB_304721
DYKDDDDK	Novus	Cat# NBP1-06712; RRID:AB_1625981
MAD2L2	Abcam	Cat# Ab180579; RRID:AB_2890174
GST	ThermoFisher	Cat# 700775; RRID: AB_2532343
Alexa-Fluor anti-mouse 488	ThermoFisher	Cat# A11029; RRID: AB_138404
Alexa-Fluor anti-rabbit 488	ThermoFisher	Cat# A11070; RRID: AB_142134
Alexa-Fluor anti-mouse 594	ThermoFisher	Cat# A11032; RRID: AB_141672
Alexa-Fluor anti-rabbit 594	ThermoFisher	Cat# A-21207; RRID:AB_141637
Alexa-Fluor anti-rat 633	ThermoFisher	Cat# A-21094; RRID:AB_141553
Alexa-Fluor anti-mouse 350	ThermoFisher	Cat# A-11045; RRID:AB_142754
Alexa-Fluor anti-rabbit 350	ThermoFisher	Cat# A-11046; RRID:AB_142716
Anti-rabbit HRP	Agilent	Cat# P0399; RRID: AB_2617141
Anti-mouse HRP	Agilent	Cat# P0447; RRID: AB_2617137
Goat anti-mouse IgM AP	Southern Biotech	Cat# 1020-04; RRID:AB_2794200
Goat Anti-Mouse IgG AP	Southern Biotech	Cat# 1036-04; RRID:AB_2794347
CD40	Miltenyi Biotec	Cat# FGK45.5; RRID:AB_871687
Anti-mouse IgG1-PE	Biolegend	Cat# 406608; RRID: AB_10551618
Anti-mouse IgG2b-APC	Biolegend	Cat# 406712; RRID: AB_2750278
Anti-mouse IgG3-FITC	BD Biosciences	Cat# 553403; RRID: AB_394840
Anti-mouse IgE-BV421	BD Biosciences	Cat# 564207; RRID:AB_2738668
Biotin (mouse)	Jackson ImmunoResearch	Cat# 200-002-211, RRID:AB_2339006
IgG	Agilent	Cat# X0903
**Bacterial and virus strains**		
BL21-CodonPlus (DE3)-RP-X Competent cells	Agilent	Cat# 260275
MAX Efficiency™ Stbl2™ Competent Cells	Life Technologies	Cat# 10268019
Edit-R inducible lentiviral Cas9 vector	Horizon Discovery	Cat# CAS11229
Lentiguide-Puro	Addgene (Stringer et al., 2019)	Cat# 104990
**Biological samples**		
HeLa nuclear cell extracts	Ipracell	Cat# CC-01-20-50
**Chemicals, peptides, and recombinant proteins**		
Hydroxyurea	Sigma Aldrich	Cat# H8627
Olaparib	Selleckchem	Cat# S1060
Talazoparib	Selleckchem	Cat# S7048
Crystal Violet	Sigma Aldrich	Cat# HT90132

(*Continued on next page*)

Continued

REAGENT or RESOURCE	SOURCE	IDENTIFIER
Doxycycline	Cayman Chemical	Cat# 14422
IdU	Sigma Aldrich	Cat# I7125
EdU	Life Technologies	Cat# 11590926
Diazo-Biotin Azide	Stratech	Cat# CLK-1041-10-JEN
Shield-1	Clontech	Cat# 632189
4-OHT	Sigma Aldrich	Cat# H6278
Oligofectamine	Life Technologies	Cat# 2252011
Lipofectamine2000	Life Technologies	Cat# 11668-019
Entellan Mounting Media	Millipore	Cat# HX61088761
Prolong Gold Anti-fade mounting medium with DAPI	Life Technologies	Cat# P36941
Vectashield (with DAPI)	Vectorlabs	Cat# H-1200
Fluoroshield	Sigma Aldrich	Cat# F6182
Protein A Sepharose	GE Healthcare	Cat# 17-0780-01
Glutathione Sepharose	GE Healthcare	Cat# 17-0756-01
Streptavadin Agarose	Sigma Aldrich	Cat# S1638
Magnetic Protein A beads	ThermoFisher	Cat# 88845
Magnetic Protein G beads	ThermoFisher	Cat# 88847
Proteinase K	Sigma Aldrich	Cat# V3021
Anti-CD43 Dynabeads	Life Technologies	Cat# 11422D
NP-CGG	Biosearch Technologies	Cat# N-5055A
NP-BSA	Biosearch Technologies	Cat# N-5050L
LPS	Sigma Aldrich	Cat# L7770
IL-4	Peprotech	Cat# 214-14-20
Zombie Near InfraRed viability dye	Biolegend	Cat# 423105
Cell Trace Violet	Life Technologies	Cat# C34571
Cell Trace Red	Life Technologies	Cat# C34572
Histone H3 - biotinylated	Active Motif	Cat# 31296
Histone H3K4me1 (EPL) - biotinylated	Active Motif	Cat# 31284
Histone H3K4me3 (EPL) - biotinylated	Active Motif	Cat# 31282
Phosphatase substrate	Sigma Aldrich	Cat# P4744
TelC-Cy5	Panagene	Cat# F1003
Imject Alum adjuvant	ThermoFisher	Cat# 77161
Critical commercial assays		
Duolink® *In Situ* Red Starter Kit Mouse/Rabbit	Sigma Aldrich	Cat# DUO92101
TnT® Quick Coupled Transcription/ Translation System	Promega	Cat# L1170
Mouse IgG ELISA Kit	Bethyl	Cat# E99-131
Mouse IgM ELISA Kit	Bethyl	Cat# E90-101
Deposited data		
Rif1 ChIP-Seq datasets from ES cells	(Foti et al., 2016)	E-MTAB-3502
BLISS datasets from ES cells	(Yan et al., 2017)	SRP099132
OK-seq datasets from ES cells	(Petryk et al., 2018)	GSM3290342
TSS locations from RNA-seq (mm10)	ENCODE	ENCSR000CGU
H3K4me1 ChIP-Seq datasets from ES cells	ENCODE	ENCFF671UNN.bed
H3K4me3 ChIP-Seq datasets from ES cells	ENCODE	ENCFF824AFZ.bed
Raw immunoblotting and immunofluorescence data	This paper	https://doi.org/10.17632/zkzrpph946.1
Mass spectrometry of HeLa-C-Flap cells	ProteomeXchange via PRIDE	PXD032231

(*Continued on next page*)

Continued

REAGENT or RESOURCE	SOURCE	IDENTIFIER
**Experimental models: cell lines**		
HeLa-H3-GFP (WT and K4A)	Hiroshi Kimura (Sato et al., 2012)	N/A
HeLa	ATCC	Cat# CCL-2
HEK-293	ATCC	Cat# CRL-1573
HeLa Kyoto	Simon Boulton	RRID: CVCL_1922
HeLa-CFlap	Simon Boulton (Higgs et al., 2015)	N/A
HeLa-CFlap-BOD1L	Simon Boulton (Higgs et al., 2015)	N/A
U-2-OS	ATCC	Cat# HTB-96
U-2-OS Flp-In TRex	Stephen Taylor (University of Manchester)	N/A
U-2-OS-*FokI*	Roger Greenberg (Shanbhag and Greenberg, 2013)	N/A
U-2-OS-Flp-In-FLAG-SETD1A variants	Grant Stewart (Higgs et al., 2018)	N/A
SETD1A patient LCLs	Tjitske Kleefstra (Kummeling et al., 2021)	N/A
Bod1l$^{F/F}$ or *Bod1l*$^{+/+}$ MEFs	This paper	N/A
**Experimental models: organisms/strains**		
Mouse: Cd19$^{tm1(cre)Cgn}$	MGI	Cat# 1931143
Mouse: *Gt(ROSA)26Sor*$^{tm9(cre/ESR1)Arte}$	MGI	Cat# 3763211
Mouse: Bod1l$^{F/F}$ R26$^{CreERT2/+}$	This paper	N/A
Mouse: Bod1l$^{F/F}$ R26$^{+/+}$	This paper	N/A
Mouse: Bod1l$^{+/+}$ Cd19$^{+/Cre}$	This paper	N/A
Mouse: Bod1l$^{F/F}$ Cd19$^{+/Cre}$	This paper	N/A
**Oligonucleotides**		
SETD1A siRNA (3’ UTR)	Qiagen	Cat# SI05029045
SETD1A siRNA (SmartPool; SP)	Dharmacon	Cat# L-022793-01-0010
BOD1L siRNA (SP)	Dharmacon	Cat# L-017033-02-0005
RIF1 siRNA (SP)	Dharmacon	Cat# L-027983-01-0005
SETD1B siRNA (SP)	Dharmacon	Cat# J-027025-09-0005
KMT2A siRNA (SP)	Dharmacon	Cat# L-009914-00-0005
KMT2B siRNA (SP)	Dharmacon	Cat# L-009670-00-0005
KMT2C siRNA (SP)	Dharmacon	Cat# L-007039-00-0005
KMT2D siRNA (SP)	Dharmacon	Cat# L-004828-00-0005
KMT2E siRNA (SP)	Dharmacon	Cat# L010580-00-0005
MRE11 siRNA (SP)	Dharmacon	Cat# L-009271-00-0005
BRCA1 siRNA (SP)	Dharmacon	Cat# L-003461-00-0005
MAD2L2 siRNA (SP)	Dharmacon	Cat# L-003272-00-0005
CtIP siRNA (GCUAAAACAGG AACGAAUCdTdT)	Dharmacon	Cat# CTM-675072
Control siRNA (luciferase) (CGUACGCGG AAUACUUCGdTdT)	Dharmacon	Cat# CTM-334043
BOD1L F3 Fwd (AGAACGGTCGACACAA AGAGCTTGTTAGAAGAGAAA)	Sigma Aldrich	N/A
BOD1L F3 Rev (ACGGATAGCGGCCGCA GTTGCCACATCCTCAGTTTGTCC)	Sigma Aldrich	N/A
BOD1L F4 Fwd (ATCACCAAGGAGGGCG GCCTGGTGGACATGGCCAAG)	Sigma Aldrich	N/A
BOD1L F4 Rev (GGAGATGGTGGTGTCCT CCACCTTCTCCAGCTG)	Sigma Aldrich	N/A

(*Continued on next page*)

Continued		
REAGENT or RESOURCE	SOURCE	IDENTIFIER
BOD1L F5 Fwd (GAGTCCGCCGAGGGCG ACTCCCAGATCGGCACCGTG)	Sigma Aldrich	N/A
BOD1L F5 Rev (GTTGGCGTTGCCCTCCAG GCCCCGGCCGGCGGAGTA)	Sigma Aldrich	N/A
BOD1L F6 Fwd (GAGTGAGTCGACTTGGCA GTGAGCACCCAGGAGGGG)	Sigma Aldrich	N/A
BOD1L F6 Rev (TTGTAGAGGGGCCGCTTA TCGCTTCGCTTTTTTCACAGG)	Sigma Aldrich	N/A
Bod1l gRNA:<br>5': 5'-TAGTACTGCAGCTACTCCA-3';<br>3': 5'-ACAGGAACATGCATTTCTGC-3'	Sigma Aldrich	N/A
RIF1 pET23a Fwd (ATAAAGAATGCGGCCGC TAAACTATAAATAGAATTTTCATGGGA)	Sigma Aldrich	N/A
RIF1 pET23a Rev (CGCGGATCCGCGATGA CGGCCAGGGGTCAG)	Sigma Aldrich	N/A
RIF1-HEATless Fwd (AAACAAAAATTTCTGCTCCTGTTG)	Sigma Aldrich	N/A
RIF1-HEATless Rev (ACCCATTTGCTGTCCACC)	Sigma Aldrich	N/A
TRF2 gRNA:TCTGTCTGAAGTCCCCGTAC	Sigma Aldrich	N/A
FokI ChIP primer 1 Fwd (GGAAGATGTCC CTTGTATCACCAT)	Sigma Aldrich (Shanbhag and Greenberg, 2013)	N/A
FokI ChIP primer 1 Rev (TGGTTGTCAACAGAG TAGAAAGTGAA)	Sigma Aldrich (Shanbhag and Greenberg, 2013)	N/A
FokI ChIP primer 2 Fwd (GCTGGTGTGGCCAATGC)	Sigma Aldrich (Shanbhag and Greenberg, 2013)	N/A
FokI ChIP primer 2 Rev (TGGCAGAGGGAA AAAGATCTCA)	Sigma Aldrich (Shanbhag and Greenberg, 2013)	N/A
FokI ChIP primer 3 Fwd (GGCATTTCAGTCAG TTGCTCAA)	Sigma Aldrich (Shanbhag and Greenberg, 2013)	N/A
FokI ChIP primer 3 Rev (TTGGCCGATTCATTAATGCA)	(Shanbhag and Greenberg, 2013)	N/A
FokI ChIP primer 4 Fwd (GGCATTTCAGTCA GTTGCTCAA)	Sigma Aldrich (Shanbhag and Greenberg, 2013)	N/A
FokI ChIP primer 4 Rev (GATCCCTCGAGG ACGAAAGG)	Sigma Aldrich (Shanbhag and Greenberg, 2013)	N/A
Bod1l-common (CCAGCATGGTGCATTTTATG)	Sigma Aldrich	N/A
Bod1l-WT (GAGGTTGAGAGAGGCACGAC)	Sigma Aldrich	N/A
Bod1l-mut (GAACCCTTTCCCACACCAC)	Sigma Aldrich	N/A
Recombinant DNA		
pGEX-3x-BOD1L F1 (aa 1-600 of BOD1L)	(Higgs et al., 2018)	N/A
pGEX-3x-BOD1L F2 (aa 500-1000 of BOD1L)	(Higgs et al., 2018)	N/A
pGEX-5x-BOD1L F3 (aa 900-1508 of BOD1L)	This paper	N/A
pGEX-5x-BOD1L F4 (aa 1400-2001 of BOD1L)	This paper	N/A
pGEX-5x-BOD1L F5 (aa 1900-2501 of BOD1L)	This paper	N/A
pGEX-5x-BOD1L F6 (aa 2399-3051 of BOD1L)	This paper	N/A
pET23a-RIF1 (aa1-2446 of RIF1)	This paper	N/A
pET23a-RIF1-ΔHEAT (aa978-2446 of RIF1	This paper	N/A
pLPC-Myc-TRF2$^{\Delta B \Delta M}$	(Chapman et al., 2013)	N/A
pCDNA5-FRT-T/O-eGFP-RIF1 (aa1-2446 of RIF1)	Escribano-Diaz et al., 2013)	N/A
pCDNA5-FRT-T/O-eGFP-RIF1-HEAT (aa1-411 of RIF1)	This paper	N/A
pLX330-LMNA-gRNA#1	Graham Dellaire (Pinder et al., 2015)	N/A
pCR2.1-Clover-LMNA-donor#1	Graham Dellaire (Pinder et al., 2015)	N/A
pmax-GFP	Lonza	N/A

(Continued on next page)

Continued

REAGENT or RESOURCE	SOURCE	IDENTIFIER
pcDNA3	Life Technologies	V79020
pcDNA3/HA-FLAG-KDM5A	Rob Klose (Klose et al., 2007)	N/A
pcDNA3/HA-FLAG-KDM5A-H483A	Rob Klose (Klose et al., 2007)	N/A
Software and algorithms		
FlowJo	BD	RRID: SCR_008520
ImageJ	NIH	RRID: SCR_003070
Nikon Elements (v4.5)	Nikon	RRID: SCR_014329
GraphPad Prism	GraphPad Software	RRID: SCR_002798
Galaxy	(Afgan et al., 2018)	N/A
X-calibur	ThermoFisher	N/A
MaxQuant	(Cox and Mann, 2008)	N/A
Perseus	(Tyanova et al., 2016)	N/A
EaSeq	Mads Lerdrup, University of Copenhagen	N/A

RESOURCE AVAILABILITY

Lead contact

Further information and requests for resources and reagents should be directed to the lead contact, Martin Higgs (m.r.higgs@bham.ac.uk).

Materials availability

All materials are available upon reasonable request to the lead contact, Martin Higgs (m.r.higgs@bham.ac.uk).

Data and code availability

- Mass spectrometry data have been deposited to the ProteomeXchange Consortium via the PRIDE partner repository with the dataset identifier PXD032231. Original western blot images have been deposited at Mendeley and are publicly available as of the date of publication. The DOIs for these data are listed in the key resources table. Microscopy data reported in this paper will be shared by the lead contact, Martin Higgs (m.r.higgs@bham.ac.uk), on reasonable request.
- This paper does not report original code.
- Any additional information required to reanalyze the data reported in this paper is available from the lead contact upon request.

EXPERIMENTAL MODEL AND SUBJECT DETAILS

Cell lines and culture

HeLa (ATCC), HeLa Kyoto, U-2-OS-FokI (Shanbhag and Greenberg, 2013), HEK-293 (ATCC), HeLa-CFlap-BOD1L and HeLa-H3-GFP cells (Higgs et al., 2018; Sato et al., 2012) were cultured in Dulbecco's modified Eagle's medium supplemented with 10 % fetal calf serum (FCS) (Life Technologies) and penicillin/streptomycin. Patient-derived lymphoblastoid cell lines (LCLs) (Kummeling et al., 2021) were maintained in RPMI 1640 medium supplemented with 15% FCS and penicillin/streptomycin. U-2-OS cells (ATCC) were cultured in McCoys 5A medium (Life Technologies), supplemented with 10 % FBS and penicillin/streptomycin. U-2-OS-FLAG-SETD1A FL and ΔSET cells (Higgs et al., 2018) were maintained in McCoys 5A medium, supplemented with 10 % Tet-free FBS and penicillin/streptomycin. Expression of SETD1A variants was induced by addition of doxycycline (Cayman Chemical).

Mouse embryonic fibroblasts (MEFs) were derived at 13.5dpc using standard protocol and cultured in Dulbecco's modified Eagle's medium (DMEM) (Invitrogen) supplemented with 15 % fetal bovine serum (FBS) and 1 % penicillin-streptomycin (Invitrogen). MEFs immortalized by Large T-SV40 were maintained with 10 % FBS. Deletion of floxed alleles in $Bod1l^{+/+}$ $R26^{CreERT2/+}$ and $Bod1l^{F/F}$ $R26^{CreERT2/+}$ MEFs was performed by treating the cells with 500 nM of 4-hydroxytamoxifen (4-OHT) for 16 h and successful deletion was tested by genotyping PCR with validated primers.

HeLa Kyoto cells were transduced with the Edit-R inducible lentiviral Cas9 vector (Horizon Discovery), selected with blasticidin and single cell clones were seeded by limiting dilution in a 96 well plate. Cas9 editing efficiency and Dox regulation was tested as described previously (Hewitt et al., 2021), and a clone with tightly regulated doxycycline-induced Cas9 activity was selected for subsequent experiments.

Mouse models

Conditional mice for BOD1L were generated via CRISPR-Cas9 based repair by pronuclear microinjection of Cas9 protein, guide RNA (5' : 5'-TAGTACTGCAGCTACTCCA-3'; 3': 5'-ACAGGAACATGCATTTCTGC-3') and repair templates containing Lox P sites to flank exon 3 of the mouse Bod1l gene. Correct targeting was verified by sequencing of founder (F0) and first generation (F1) mice. Conditional *Bod1l* mice have then been bred to a tamoxifen inducible Cre strain (*Gt(ROSA)26Sor*$^{tm9(cre/ESR1)Arte}$; MGI: 3763211). Aged-matched male and female mice aged 8-16 weeks were used for all experiments. The precise numbers, genotype and sex of the animals used is detailed in Table S1.

Bod1l$^{F/F}$ *R26*$^{CreERT2/+}$ and *Bod1l*$^{F/F}$ *R26*$^{+/+}$ mice were administered 4.5 mg of tamoxifen (4-OHT; Sigma) 3 times over 5 days and then sacrificed for downstream experiments 7 days after tamoxifen administration. Correct deletion was verified by PCR genotyping using the following primers (Bod1L-common: 5'-CCAGCATGGTGCATTTTATG-3'; Bod1L-WT: 5'-GAGGTTGAGAGAGGCACGAC-3'; Bod1L-Mut: 5'-GAACCCTTTCCCACACCAC-3').

Conditional Bod1l mice were also mated with B cell specific deleter Cre (*Cd19-Cre*; MGI: 1931143). *Bod1l*$^{+/+}$ *Cd19*$^{+/Cre}$ and *Bod1l*$^{F/F}$ *Cd19*$^{+/Cre}$ mice were immunized intraperitoneally with 50 mg of NP-CGG (Biosearch Technologies) resuspended in Imject Alum adjuvant (Pierce, Thermo Fisher Scientific). Blood samples were collected from the tail vein at 0, 7, 14, 21 and 28 days after immunization.

All animal experimentations were undertaken in compliance with UK Home Office legislation under the Animals (Scientific Procedures) Act 1986 under project license number 70/8527 and following the ARRIVE guidelines.

Plasmids and cloning

GST-tagged BOD1L fragments were amplified by PCR from human cDNA and cloned into the SalI-NotI restriction sites of pGEX-5X. Constructs encoding pCDNA5-FRT-T/O-eGFP-RIF1 was obtained from Dan Durocher (Escribano-Diaz et al., 2013). RIF1 was subcloned from this vector into the NotI-BamHI sites of pET23a by PCR to create pET23a-RIF1. A ΔHEAT mutant lacking aa1-977 of human RIF1 was created using a New England Biolabs Q5 Mutagenesis kit and corresponding primers. See key resources table for primer sequences and corresponding amino acid designations.

METHOD DETAILS

Transfections

SMARTpool siRNA (Horizon Discovery) or SETD1A 3' UTR siRNA (Qiagen) were transfected into cells using Oligofectamine (Life Technologies) at a final concentration of 100 nM. An siRNA targeting lacZ (Horizon Discovery) was used as a control. Plasmid DNA was transfected into cells using Lipofectamine (Life Technologies) and amounts of DNA transfected are indicated in individual experiments.

ELISA

Enzyme-linked immunosorbent assays (ELISAs) were used to quantify the production of NP-specific antibodies in mice serum. 96 well plates were coated with 1 μg/ml NP-BSA (Biosearch Technologies) in bicarbonate buffer, blocked with 5% milk in PBS and incubated with serial dilutions of serum collected at different time points from immunized mice. Plates were then incubated with alkaline phosphatase-coupled antibodies against mouse IgM and IgG1 (Southern Biotech). Phosphatase substrate (Sigma) was used for detection and optical density measured at 405nm. For IgG1, pooled blood from post-immunisation wild type mice was used as a standard and serially diluted into a standard curve. The first dilution was established as 1000 arbitrary units. For IgM, pooled blood from day 7 was used as a standard.

Ig concentrations in mouse serum or culture supernatants were determined by sandwich ELISA. Total IgG, IgM was measured with mouse IgG and IgM ELISA kits, respectively (Bethyl Laboratories), according to the manufacturer instructions. Mouse serum with known Ig concentrations of each Ig was used as a standard.

Antibodies

Antibodies used in this study are detailed in the key resources table.

Clonogenic survival assays

HeLa or HeLa-H3-GFP cells transfected with siRNA were plated at low density and exposed to increasing doses of ionizing radiation or increasing concentrations of Olaparib or Talazoparib (Selleckchem). Colonies were fixed and stained after 10 days with 0.5 % crystal violet (Sigma-Aldrich) in ddH_2O. Data are expressed as a percentage survival normalized to an untreated control for each siRNA.

Immunofluorescence, microscopy and image analysis

HeLa, U-2-OS-FokI, HeLa-H3-GFP and U-2-OS-FLAG-SETD1A cells were grown on glass coverslips. LCL cells were allowed to attach to poly-L-lysine coated microscope slides by gravity. Cells were irradiated with 3 Gy of ionizing radiation in all cases. For overexpression of the KDM5A demethylase, HeLa cells were transfected with 4 μg of either pcDNA3, pcDNA3/HA-FLAG-KDM5A

or pcDNA3/HA-FLAG-KDM5A-H483A plasmid (Klose et al., 2007) and incubated for 48 h prior to fixation. In all cases, cells were permeabilised with nuclear extraction buffer (20 mM NaCl, 3 mM MgCl2, 300 mM sucrose, 10 mM PIPES, 0.5 % Triton X-100, pH 6.8) for 5 min on ice (cells on coverslips) or 2 min at room temperature (cells on slides) and then fixed with 4% paraformaldehyde for 10 min at RT. Following fixation, cells were washed three times with PBS and blocked for 1 h at RT using 10 % FCS/PBS. Cells were incubated with primary antibodies diluted in 3% FCS/PBS for 1 h at RT, washed three times with PBS and incubated with Alexa Fluor-conjugated secondary antibodies diluted 1:1000 in 3 % FCS/PBS for 1 h at RT. Cells were washed three times in PBS, once with ddH_2O and then mounted with Duolink in situ mounting medium containing DAPI (Sigma-Aldrich). Images were taken using a Nikon E600 Eclipse equipped with a 60 x oil lens and foci numbers and intensity analysed using ImageJ software.

A similar protocol was used for MEFs. Briefly MEFs were preextracted with nuclear extraction buffer (20mM Hepes pH 8.0, 20mM NaCl, 5mM MgCl2, 1mM DTT, 0.5% NP40, 300 mM sucrose) for 20 min on ice, then fixed with 2% PFA for 20 min and blocked for 30min in antibody dilution buffer (ADB: 0.1% Triton X-100, 0.1% Saponin, 10% goat serum, PBS). MEFs were incubated with primary antibodies overnight at 4 degrees, washed and then incubated with secondary antibodies (Alexa Fluor) in ADB for 1 h at RT. Cells were them washed and mounted in Prolong antifade mountant with DAPI (Life Technologies). Images were taken using an Olympus FV1000 confocal microscope using a 40X lens and were analysed using Image J software.

DNA fibre analysis

DNA fibre analysis was carried out as described previously (Petermann et al., 2010) with minor modifications. Forty-eight hours post transfection with siRNA, U-2-OS cells were incubated with 250 μM IdU for 24 h and then exposed to 10 Gy of ionizing radiation. One hour after treatment, cells were harvested and DNA fibres spread onto microscope slides. Immunostaining of DNA was carried out as described previously (Petermann et al., 2010), omitting the HCl denaturation step to allow quantification of native single-stranded DNA structures. The lengths of labelled tracts were measured using ImageJ and arbitrary lengths converted into micrometers using scale bars captured on images using a Nikon E600 Eclipse equipped with a 60 x oil lens.

Proximity ligation assay

Forty-eight hours post transfection with siRNA, HeLa cells were seeded onto glass coverslips and irradiated with 3 Gy of ionizing radiation. Eight hours after treatment, cells were permeabilised with nuclear extraction buffer for 5 min on ice and then fixed with 4 % paraformaldehyde for 10 min at RT. Following fixation, cells were washed three times with PBS and blocked for 1 h at RT using 3 % BSA/PBS. Cells were incubated with primary antibodies to RIF1, SETD1A and ɣH2AX diluted 1:100 in 3% FCS/PBS for 1 h at RT and then proximity ligation was carried out using a Duolink Detection Kit in combination with anti-Mouse PLUS and anti-Rabbit MINUS PLA Probes (Sigma-Aldrich) according to the manufacturer's instructions. Images were taken using a Nikon E600 Eclipse equipped with a 60 x oil lens and foci numbers and intensity analysed using ImageJ software

Alternatively, EdU-PLA to detect proteins at nascent DNA was performed as described (Higgs et al., 2018). Cells were exposed to 4 mM HU for 5 h before being permeabilised and fixed as above. EdU was then conjugated to biotin by incubating cells in Click reaction buffer for 1 h at room temperature containing 10 μM Diazo-biotin Azide, 10 mM sodium ascorbate, and 1 mM copper (II) sulfate in PBS. Following the Click reaction, cells were blocked in ABD before being incubated in primary antibodies and proximity ligation carried out as above.

Chromatin immunoprecipitation

Chromatin immunoprecipitation was performed as described previously (McNee et al., 2017). Briefly, 48 h post transfection with siRNA, double-strand breaks were induced in U-2-OS-FokI cells by addition of 1 μM Shield1 ligand (Clontech) and 1 μM 4-hydroxytamoxifen (Sigma-Aldrich) for 4 h, or left untreated. Samples were crosslinked with 1 % formaldehyde and neutralized with 0.125 M glycine. Cells were lysed and DNA sheared to 300-1000 bp by sonication. Samples were pre-cleared with rabbit immunoglobulins (Dako) and chromatin was co-immunoprecipitated with antibodies to BOD1L, SETD1A, RIF1, histone H3, H3K4me1, H3K4me2 and H3K4me3. Protein-DNA complexes were washed and eluted from magnetic protein G beads, cross-links were reversed and samples treated with proteinase K. DNA was purified using a PCR purification kit (Qiagen) and quantified by qPCR using 4 primer pairs (see Figure 6O for location of amplicons, and key resources table for sequences).

Western blotting, pull-downs and immunoprecipitation

For western blotting, cells were lysed in UTB buffer (8 M Urea, 50 mM Tris, 150 mM β-mercaptoethanol, and protease inhibitor cocktail (Roche)). Cell extracts were clarified via centrifugation and the protein concentration in the lysate was determined by a Bradford assay (Bio-Rad). Proteins were separated by SDS-PAGE and transferred to a nitrocellulose membrane. Membranes were incubated with primary antibodies diluted in 5 % dried milk/TBST overnight and then in HRP-conjugated secondary antibody for 1 h at RT. Proteins were visualised using ECL detection reagents (GE Healthcare).

For immunoprecipitations, HeLa nuclear cell extracts (Ipracell) were mixed with 5 μg of the indicated antibodies or IgG and rotated at 4 °C for 3 h. Extracts were clarified by centrifugation at 44,000 xg and immune complexes were isolated via binding to protein A magnetic beads (Pierce). Samples were then analysed by western blotting as described above.

For GST-pulldowns, HeLa nuclear cell extracts were mixed with 1 μg of GST fusion protein or purified GST and rotated at 4 °C for 3 h. Protein complexes were isolated via binding to glutathione sepharose (GE Healthcare). Samples were then analysed by western blotting as described above.

For histone pull-downs, HeLa cells were subjected to 20 Gy ionizing radiation and 1 h later lysed in NETN buffer (250 mM NaCl, 50 mM Tris pH 8, 1 % NP-40, 2 mM $MgCl_2$, 90 units/ml benzonase and protease inhibitor cocktail). Cell extracts were clarified via centrifugation and the protein concentration in the lysate was determined by a Bradford assay. Alternatively, RIF1 was *in vitro* transcribed and translated using a Promega T7-based transcription/translation kit with T7 PCR enhancer at 30 ^{O} for 75 minutes, and protein quantified using BSA standards. Lyophilised recombinant biotinylated H3, H4K4me1 or H3K4me3 (EPL) (Active Motif) were resuspended at 1 μg/μl in 25 mM Tris pH 7.5, 150 mM NaCl, 5 % glycerol. HeLa whole cell extracts or 200 ng of *in vitro* translated protein were mixed with 1 μg of recombinant histones in binding buffer (20 mM Tris pH 7.5, 150 mM KCl, 300 mM sucrose, 1 mM $MgCl_2$ and protease inhibitor cocktail) and rotated at 4 °C for 3 h. Protein complexes were isolated via binding to streptavidin agarose beads (Sigma-Aldrich) and washed in binding buffer with 0.1% Triton x-100. Samples were then analysed by western blotting as described above.

Metaphase spreads and telomere FISH

Telomere fusions were assessed as follows: a HeLa Kyoto clone inducibly expressing Cas9 (above) was transduced with Lentiguide-Puro (Addgene #104990; Stringer et al., 2019) modified to contain a sgRNA targeting human TRF2 at an MOI of 0.3 and transductants were selected with 2 ug/ml puromycin for 3 days. For experiments, Cas9 expression was induced for 5 days using 1 ug/ml doxycycline treatment. 48 hours later, cells were transfected with the indicated siRNAs as described above. 120 hours after doxycycline treatment, 72 hours after siRNA treatment, cells were harvested for immunoblotting and telomere fusion assays which were performed as described previously (Ruis et al., 2021). Alternatively, HeLa cells were transfected with 6 μg pLPC-Myc-TRF2$^{\Delta B\Delta M}$ plasmid (Chapman et al., 2013) 48 h post transfection with siRNA, and incubated for a further 48 h.

Radial chromosomes were quantified in DAPI stained metaphase spreads from transfected HeLa and H3-GFP cells. Colcemid (ThermoFisher Scientific) was added to HeLa or HeLa-H3-GFP cells at a final concentration of 0.1 μg/ml 3 h prior to harvesting. Cells were harvested by trypsinization, exposed to 0.075 M KCl for 10 min at 37 °C and fixed in 3:1 methanol:acetic acid solution. Cells were dropped onto microscope slides pre-treated with fixative solution, placed on a heating block at 80 °C for 1 min and allowed to dry. To assess radial chromosome formation, slides were mounted with Duolink in situ mounting medium containing DAPI (Sigma-Aldrich). To assess telomere fusions, slides were incubated with TelC-Cy5 (Panagene) diluted in hybridisation buffer (70 % deionised formamide, 0.5 % blocking reagent, 10 mM Tris pH 7.5) for 2 h at RT. Slides were washed twice with 70 % formamide, 10 mM Tris pH 7.5, three times in PBS and mounted with Duolink in situ mounting medium containing DAPI (Sigma-Aldrich). Images were taken using a Nikon E600 Eclipse microscope equipped with a 100 x oil lens and radial chromosome numbers and telomere fusions analysed using ImageJ software.

Class switch recombination assays

Ex vivo CSR assays from lymphocytes were carried out as previously described (Chapman et al., 2013). Briefly B cells were purified from single-cell suspensions of mouse spleens by magnetic negative selection using anti-CD43 Dynabeads (Life Technologies). B cells (3×10^5 per well in a 96-well plate) were cultured in RPMI supplemented with 10% FCS, 100 U/ml penicillin, 100 ng/ml streptomycin, 2 mM l-glutamine, 1× MEM nonessential amino acids, 1 mM sodium pyruvate and 50 μM β-mercaptoethanol. B cells were stimulated with 10 μg/ml LPS (Sigma, L7770-1MG), 10 ng/ml mouse recombinant IL-4 (Peprotech, 214-14-20), and agonist anti-CD40 antibody (5 μg/ml; Miltenyi Biotec; FGK45.5). Cultures were grown at 37 °C with 5% CO2 under ambient oxygen conditions. Four days after seeding, stimulated B cells were analysed using a BD LSRFortessa and analysis was performed using FlowJo. Cells were resuspended in FACS buffer, blocked with Mouse BD Fc Block, and immunostained with the following antibodies: anti-mouse IgG1-PE (1:200, Biolegend), anti-mouse IgG2b-APC (1:200, Biolegend), anti-mouse IgG3-FITC (1:200, BD Biosciences) and anti-mouse IgE-BV421 (1:200, BD Biosciences). Live/dead cells were discriminated after staining with Zombie Near InfraRed viability dye. Cell proliferation was assessed using Cell Trace Violet or Cell Trace Red according to manufacturer's instructions (CellTrace, Life Technologies).

Homologous recombination assay

Homologous recombination was measured using a CRISPR-based assay (Pinder et al., 2015). Briefly, 24 h post transfection with siRNA, HeLa cells were seeded onto glass coverslips and transfected with 0.5 μg pLX330-LMNA-gRNA#1 and 0.5 μg pCR2.1-Clover-LMNA-donor#1 (Pinder et al., 2015) or 0.1 μg pmax-GFP plasmids (Lonza). Forty-eight hours later cells were fixed with 4 % paraformaldehyde for 10 min at RT and then permeabilised with 0.5 % Triton-X-100/PBS for 5 min at RT. Cells were blocked for 1 h at RT using 10 % FCS/PBS and then mounted with Duolink in situ mounting medium containing DAPI (Sigma-Aldrich). Homologous recombination was quantified by counting green-ringed cells and normalised to the efficiency of pmax-GFP transfection.

Mass spectrometry and proteomics

HeLa-C-Flap-BOD1L and HeLa-C-Flap cells were collected and lysed in benzonase lysis buffer (20 mM Tris-Cl, pH 7.5, 75 mM NaCl, 5% glycerol, 2 mM MgCl2, 1% CHAPS, 30 U ml−1 benzonase, protease inhibitors). NaCl concentration was adjusted to 150 mM,

CellPress
OPEN ACCESS

EDTA to 3 mM and lysates were cleared by centrifugation. Supernatants were pre-cleared with Protein G agarose beads for 30 min at 4 °C. Pre-cleared lysates were incubated with anti-Flag affinity agarose resin (Sigma) for 4 h at 4 °C. Beads were washed five times with wash buffer (20 mM Tris-Cl, pH 7.5, 300 mM NaCl, 3 mM EDTA, 1% CHAPS) and once with PBS. Bound proteins were eluted by boiling in SDS–PAGE sample buffer and eluates were resolved on NuPAGE Bis-Tris gels (Invitrogen) and stained with Coomassie Blue (Abcam). Gel slices were excised and processed for mass spectrometry. Proteins were digested with trypsin and peptides sequentially extracted according to the protocols established by the Crick proteomics lab. Peptide mixtures were resuspended in 10 ul of 0.1% TFA to retain hydrophilic peptides on the trapping column, separated on a 50 cm, 75um I.D. Pepmap column over a 30-minute gradient and then eluted directly onto an Orbitrap instrument. X-calibur software was used to control the data acquisition. All data analysis was performed using the MaxQuant bioinformatics suite (Cox and Mann, 2008). The "light" version of intensity based absolute quantification (iBAQ) was used for label free protein quantification and data was exported to Perseus software (Tyanova et al., 2016) for viewing. The mass spectrometry proteomics data have been deposited to the ProteomeXchange Consortium via the PRIDE partner repository with the dataset identifier PXD032231.

QUANTIFICATION AND STATISTICAL ANALYSIS

Data analysis of H3K4/RIF1 binding

RIF1 ChIP-Seq (Foti et al., 2016), BLISS (Yan et al., 2017), OK-seq (Petryk et al., 2018), H3K4me1 and H3K4me3 ENCODE datasets from mouse ESCs were mapped to mm10 using Bowtie2 v.2.3.4.2 on the online platform Galaxy (Afgan et al., 2018) (https://usegalaxy.org). Alternatively, H3K4me1 and H3K4me3 ENCODE peak.bed files were used to analyse RIF1 ChP-Seq levels at H3K4me sites. Transcription start sites were identified as regions +/- 500 bp from the start of transcripts from mm10. Profiles and heatmaps were generated using the computation environment EaSeq (v1.101).

Statistical analysis

Statistical differences for IdU tract length, IdU foci, foci intensity, foci numbers, radial chromosomes and PLA were determined by Mann-Whitney rank sum test. Clonogenic survival assays were analysed by two-way ANOVA. In all other cases, statistical differences were determined by Student's *t*-test. Statistical tests were performed using GraphPad Prism Version 8.3.0 (GraphPad Software, LLC) and unless otherwise stated determined by comparison to control-treated samples. * p=<0.05; ** p=<0.01; *** p=<0.001.

Molecular Cell, Volume *82*

Supplemental information

H3K4 methylation by SETD1A/BOD1L

facilitates RIF1-dependent NHEJ

Rachel Bayley, Valerie Borel, Rhiannon J. Moss, Ellie Sweatman, Philip Ruis, Alice Ormrod, Amalia Goula, Rachel M.A. Mottram, Tyler Stanage, Graeme Hewitt, Marco Saponaro, Grant S. Stewart, Simon J. Boulton, and Martin R. Higgs

Figure S1:

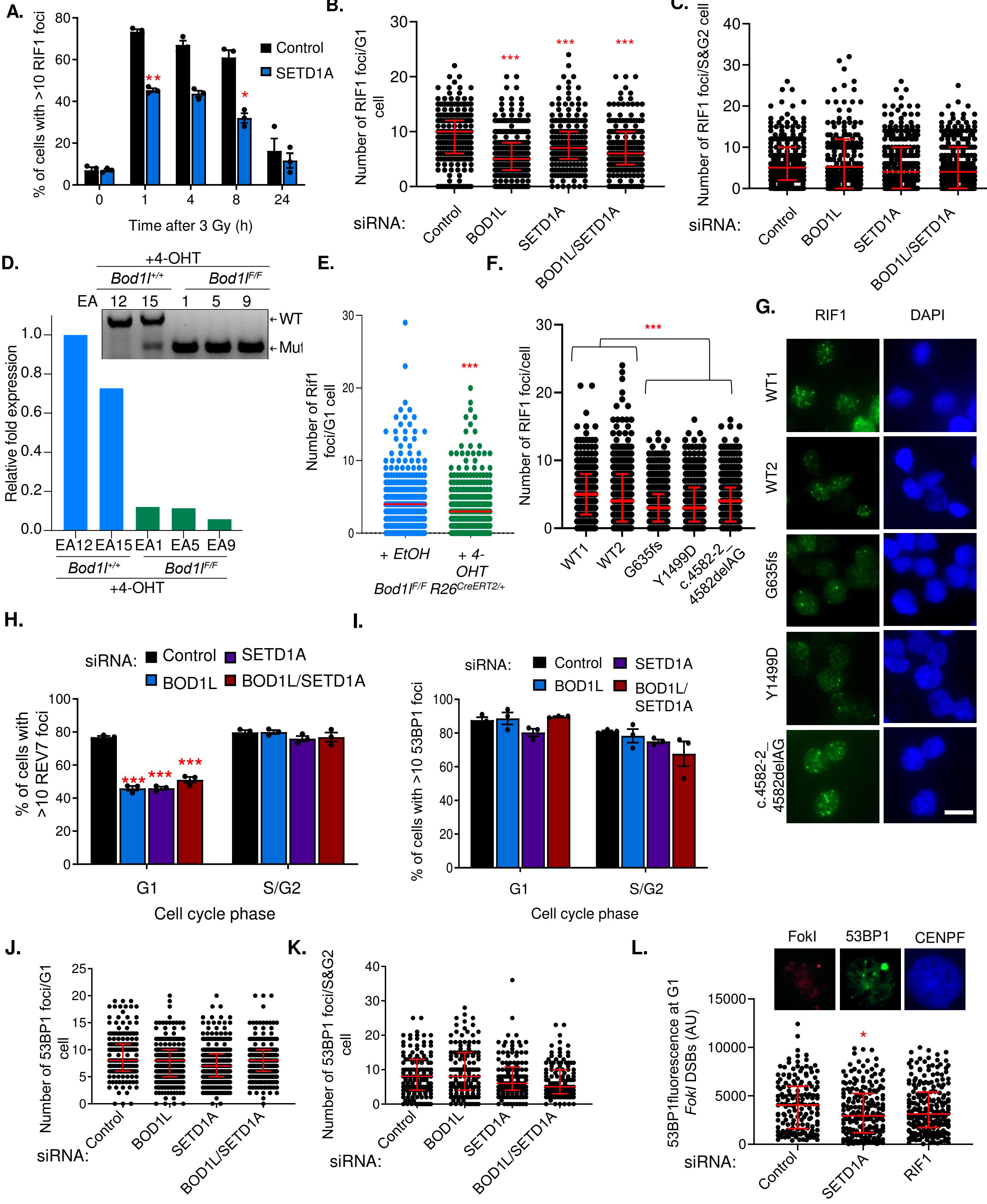
A.
% of cells with >10 RIF1 foci
Control
SETD1A
Time after 3 Gy (h)
B.
Number of RIF1 foci/G1 cell
C.
Number of RIF1 foci/S&G2 cell
siRNA:
Control
BOD1L
SETD1A
BOD1L/SETD1A
D.
+4-OHT
Bod1l+/+
Bod1lF/F
EA 12 15 1 5 9
WT
Mut
Relative fold expression
EA12 EA15 EA1 EA5 EA9
E.
Number of Rif1 foci/G1 cell
+ EtOH
+ 4-OHT
Bod1lF/F R26CreERT2/+
F.
Number of RIF1 foci/cell
WT1
WT2
G635fs
Y1499D
c.4582-2_4582delAG
G.
RIF1
DAPI
H.
siRNA:
Control
SETD1A
BOD1L
BOD1L/SETD1A
% of cells with >10 REV7 foci
G1
S/G2
Cell cycle phase
I.
% of cells with >10 53BP1 foci
J.
Number of 53BP1 foci/G1 cell
K.
Number of 53BP1 foci/S&G2 cell
L.
FokI
53BP1
CENPF
53BP1fluorescence at G1 FokI DSBs (AU)
RIF1

Figure S1, related to Figure 2: SETD1A and BOD1L are required for recruitment of RIF1, but not 53BP1, to DNA double-strand breaks. (A) HeLa cells were transfected with the indicated siRNA, exposed to ionizing radiation (IR), and RIF1 foci formation enumerated by immunofluorescence microscopy at the indicated times. **(B-C)** RIF1 foci formation was enumerated in CENPF-negative (B) or positive (C) cells from (A) at 8 h post IR. Lines denote mean values from three independent experiments. **(D)** *Bod1l*$^{F/F}$ or *Bod1l*$^{+/+}$ MEFs were treated with 4-OHT, and the expression of mutant *Bod1l* alleles (above) and relative quantification of *Bod1l* gene expression by qPCR (below) was assessed. **(E)** MEFs from (D) were irradiated, and immunostained with antibodies to CENPF and RIF1. Foci formation was enumerated by fluorescence microscopy, and the number of foci per G1 cell is indicated. Lines denote mean values from three independent experiments. **(F-G)** RIF1 foci formation and representative images of RIF1 foci in cells bearing heterozygous SETD1A mutations after exposure to IR (see Figure 2E). Scale bars = 10 µm. **(H)** HeLa cells were transfected with the indicated siRNA, irradiated, and immunostained with antibodies to CENPF and REV7. Foci formation was enumerated by fluorescence microscopy, and the number of cells with >10 foci per cell is denoted. **(I-K)** HeLa cells were transfected with the indicated siRNA, irradiated, and immunostained with antibodies to CENPF and 53BP1. Foci formation was enumerated by fluorescence microscopy, and the number of cells with >10 foci per cell (I), as well as the number of foci per cell (J-K) is indicated. Lines denote mean values from three independent experiments. **(L)** U-2-OS-FokI cells were transfected with the indicated siRNA, treated with 4-OHT and immunostained with antibodies to CENPF and 53BP1. Representative images are shown above, and fluorescence intensity per FokI-positive focus was quantified using ImageJ. Lines denote mean values from three independent experiments. Plots in all cases represent mean data from three independent experiments. Error bars = mean ± SEM, *P* values: unpaired two-tailed t-tests (A, H) or Mann-Whitney (B, C, E, F, J-L). *p≤0.05, **p ≤ 0.01 and ***p ≤ 0.001.

Figure S2:

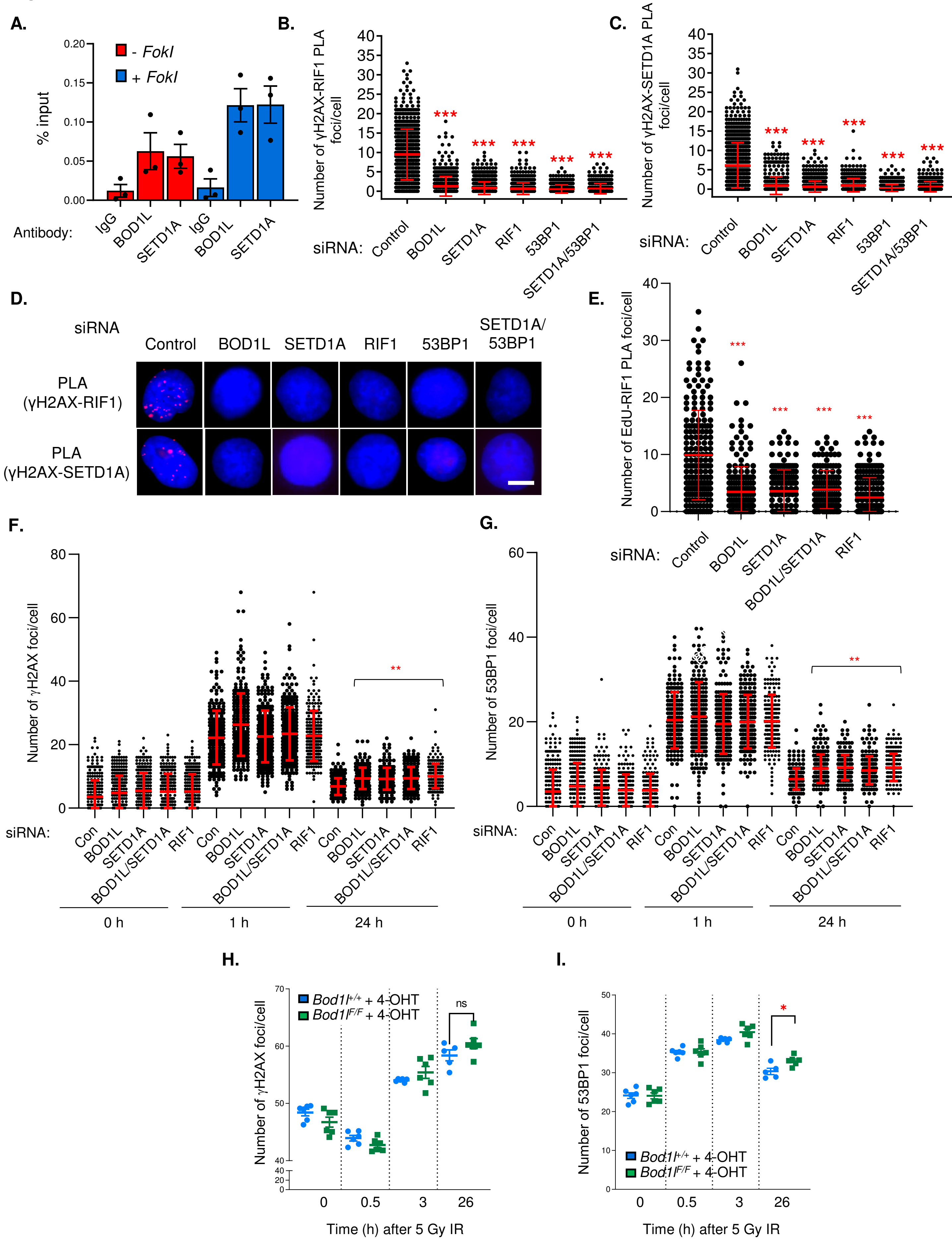

A.
- FokI
+ FokI
% input
Antibody: IgG BOD1L SETD1A IgG BOD1L SETD1A
B.
Number of γH2AX-RIF1 PLA foci/cell
siRNA: Control BOD1L SETD1A RIF1 53BP1 SETD1A/53BP1
C.
Number of γH2AX-SETD1A PLA foci/cell
siRNA: Control BOD1L SETD1A RIF1 53BP1 SETD1A/53BP1
D.
siRNA
Control BOD1L SETD1A RIF1 53BP1 SETD1A/53BP1
PLA (γH2AX-RIF1)
PLA (γH2AX-SETD1A)
E.
Number of EdU-RIF1 PLA foci/cell
siRNA: Control BOD1L SETD1A BOD1L/SETD1A RIF1
F.
Number of γH2AX foci/cell
siRNA: Con BOD1L SETD1A BOD1L/SETD1A RIF1
0 h
1 h
24 h
G.
Number of 53BP1 foci/cell
siRNA: Con BOD1L SETD1A BOD1L/SETD1A RIF1
0 h
1 h
24 h
H.
Bod1l+/+ + 4-OHT
Bod1lF/F + 4-OHT
ns
Number of γH2AX foci/cell
Time (h) after 5 Gy IR
I.
Number of 53BP1 foci/cell
Bod1l+/+ + 4-OHT
Bod1lF/F + 4-OHT
Time (h) after 5 Gy IR

Figure S2, related to Figure 2: SETD1A and BOD1L suppress DSB resection after IR. (A) U-2-OS-FokI cells were treated with 4-OHT, chromatin was isolated and immunoprecipitated with the indicated antibodies and quantified by qPCR. **(B-D)** Quantification of PLA signals between γH2AX and RIF1 (B), or between γH2AX and SETD1A (C), in HeLa cells transfected with the indicated siRNAs and exposed to IR. Representative images are shown in (D). **(E)** Quantification of PLA signals between EdU and RIF1 in HeLa cells transfected with the indicated siRNAs and exposed to 5 mM hydroxyurea for 4 hours. **(F-G)** Enumeration of gH2AX (F) and 53BP1 (G) foci in HeLa cells from Figure 2G. Lines denote mean values from three independent experiments. **(H-I)** Enumeration of γH2AX (H) and 53BP1 (I) foci in MEFs from Figure 2J. Plots in all cases represent mean data from three independent experiments. Error bars = mean ± SEM, *P* values: One-way ANOVA (H, I); Mann-Whitney (B, C, E, F, G). *p≤0.05, **p ≤ 0.01 and ***p ≤ 0.001.

Figure S3:

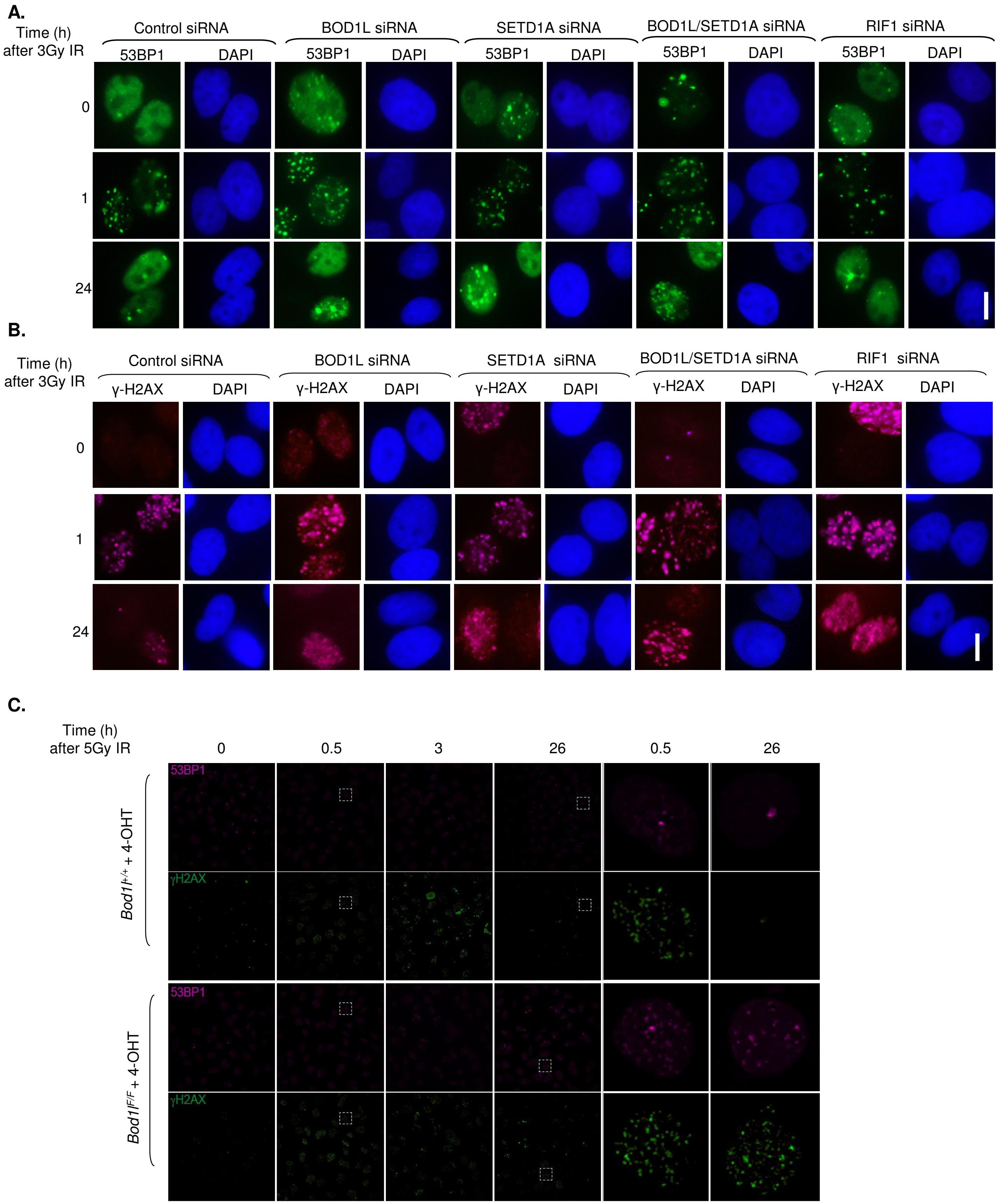
A.
Time (h) after 3Gy IR
Control siRNA
BOD1L siRNA
SETD1A siRNA
BOD1L/SETD1A siRNA
RIF1 siRNA
53BP1
DAPI
0
1
24
B.
Time (h) after 3Gy IR
Control siRNA
BOD1L siRNA
SETD1A siRNA
BOD1L/SETD1A siRNA
RIF1 siRNA
γ-H2AX
DAPI
0
1
24
C.
Time (h) after 5Gy IR
0
0.5
3
26
0.5
26
53BP1
γH2AX
Bod1l+/+ + 4-OHT
Bod1lF/F + 4-OHT

Figure S3, related to Figure 2: Loss of SETD1A and BOD1L compromises efficient DSB repair (A-C) Representative images of γH2AX or 53BP1 foci formation in irradiated HeLa cells or MEFs from Figure 2G-K. Scale bars = 10 µm.

Figure S4.

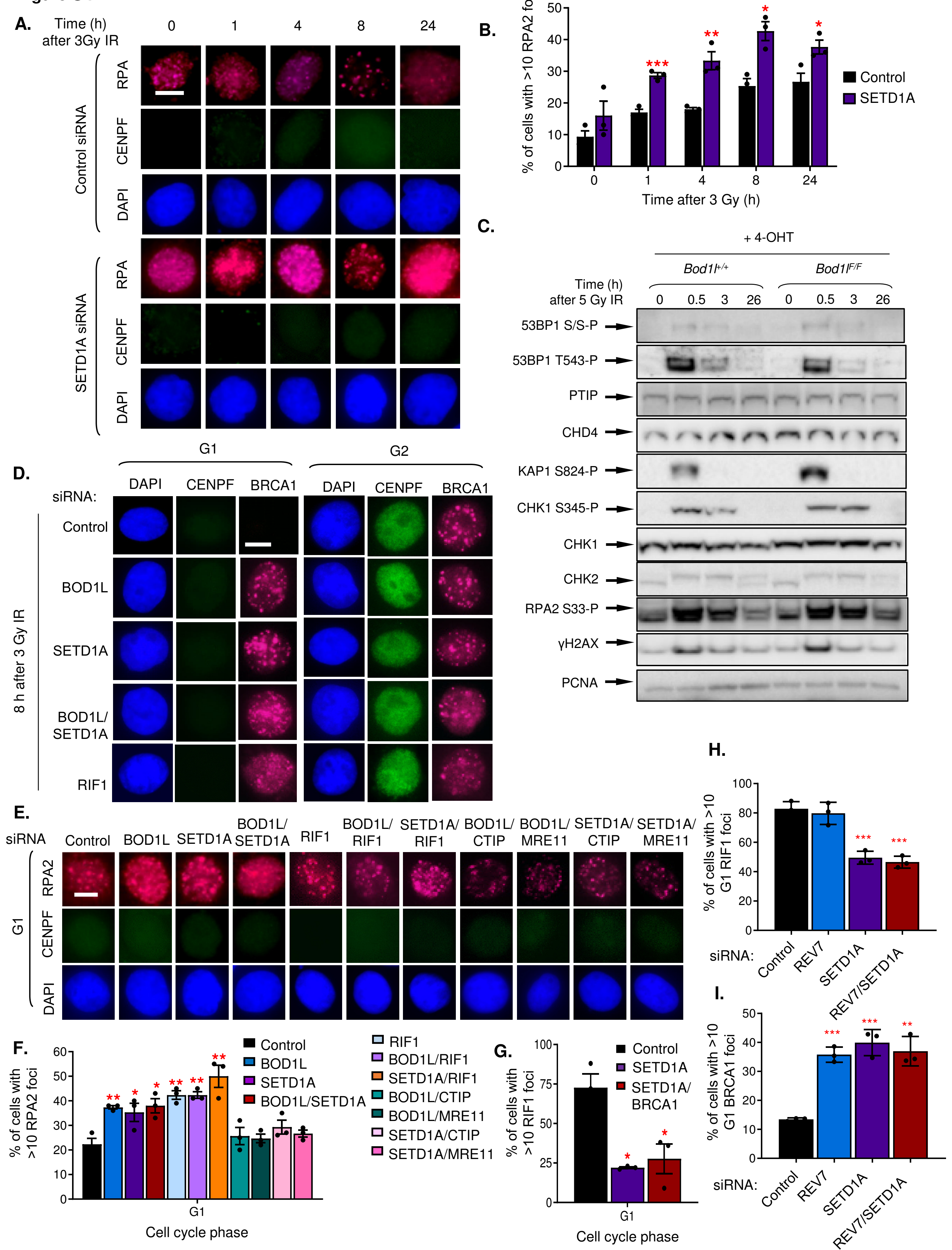
A.
Time (h) after 3Gy IR
0
1
4
8
24
Control siRNA
SETD1A siRNA
RPA
CENPF
DAPI
B.
% of cells with >10 RPA2 foci
Time after 3 Gy (h)
Control
SETD1A
C.
+ 4-OHT
Bod1l+/+
Bod1lF/F
Time (h) after 5 Gy IR
0
0.5
3
26
53BP1 S/S-P
53BP1 T543-P
PTIP
CHD4
KAP1 S824-P
CHK1 S345-P
CHK1
CHK2
RPA2 S33-P
γH2AX
PCNA
D.
G1
G2
siRNA:
DAPI
CENPF
BRCA1
Control
BOD1L
SETD1A
BOD1L/ SETD1A
RIF1
8 h after 3 Gy IR
E.
siRNA
Control
BOD1L
SETD1A
BOD1L/ SETD1A
RIF1
BOD1L/ RIF1
SETD1A/ RIF1
BOD1L/ CTIP
BOD1L/ MRE11
SETD1A/ CTIP
SETD1A/ MRE11
G1
RPA2
CENPF
DAPI
F.
% of cells with >10 RPA2 foci
Control
BOD1L
SETD1A
BOD1L/SETD1A
RIF1
BOD1L/RIF1
SETD1A/RIF1
BOD1L/CTIP
BOD1L/MRE11
SETD1A/CTIP
SETD1A/MRE11
G1
Cell cycle phase
G.
% of cells with >10 RIF1 foci
Control
SETD1A
SETD1A/ BRCA1
G1
Cell cycle phase
H.
% of cells with >10 G1 RIF1 foci
siRNA:
Control
REV7
SETD1A
REV7/SETD1A
I.
% of cells with >10 G1 BRCA1 foci
siRNA:
Control
REV7
SETD1A
REV7/SETD1A

Figure S4, related to Figure 3: Loss of BOD1L and SETD1A increases BRCA1-, CtIP- and MRE11-dependent end-resection of DNA double-strand breaks. (A-B) HeLa cells were transfected with the denoted siRNAs, irradiated, and immunostained with antibodies against RPA2 and CENPF. Representative images (A) and percentage of cells with >10 RPA2 foci (B) enumerated by immunofluorescence microscopy are shown. Scale bars = 10 µm. **(C)** *Bod1l*$^{F/F}$ or *Bod1l*$^{+/+}$ MEFs were treated with 4-OHT, exposed to ionizing radiation, and whole cell extracts from the indicated time points immunoblotted with the indicated antibodies. **(D)** Representative images of ionizing radiation-induced BRCA1 foci in HeLa cells from Figure 3J following BOD1L, SETD1A or RIF1 depletion. **(E-F)** HeLa cells were transfected with the denoted siRNAs, irradiated, and immunostained with antibodies against RPA2 and CENPF. Representative images (E) and percentage of cells with >10 RPA2 foci (F) enumerated by immunofluorescence microscopy are shown. **(G-I)** HeLa cells were transfected with the denoted siRNAs, irradiated, immunostained with antibodies against either RIF1 and CENPF (G-H) or BRCA1 and CENPF (I), and foci enumerated by immunofluorescence microscopy. Plots in all cases represent mean data from three independent experiments. Error bars = mean ± SEM, *P* values: unpaired two-tailed t-test (A, F, G, H, I). *p≤0.05, **p ≤ 0.01 and ***p ≤ 0.001.

Figure S5.

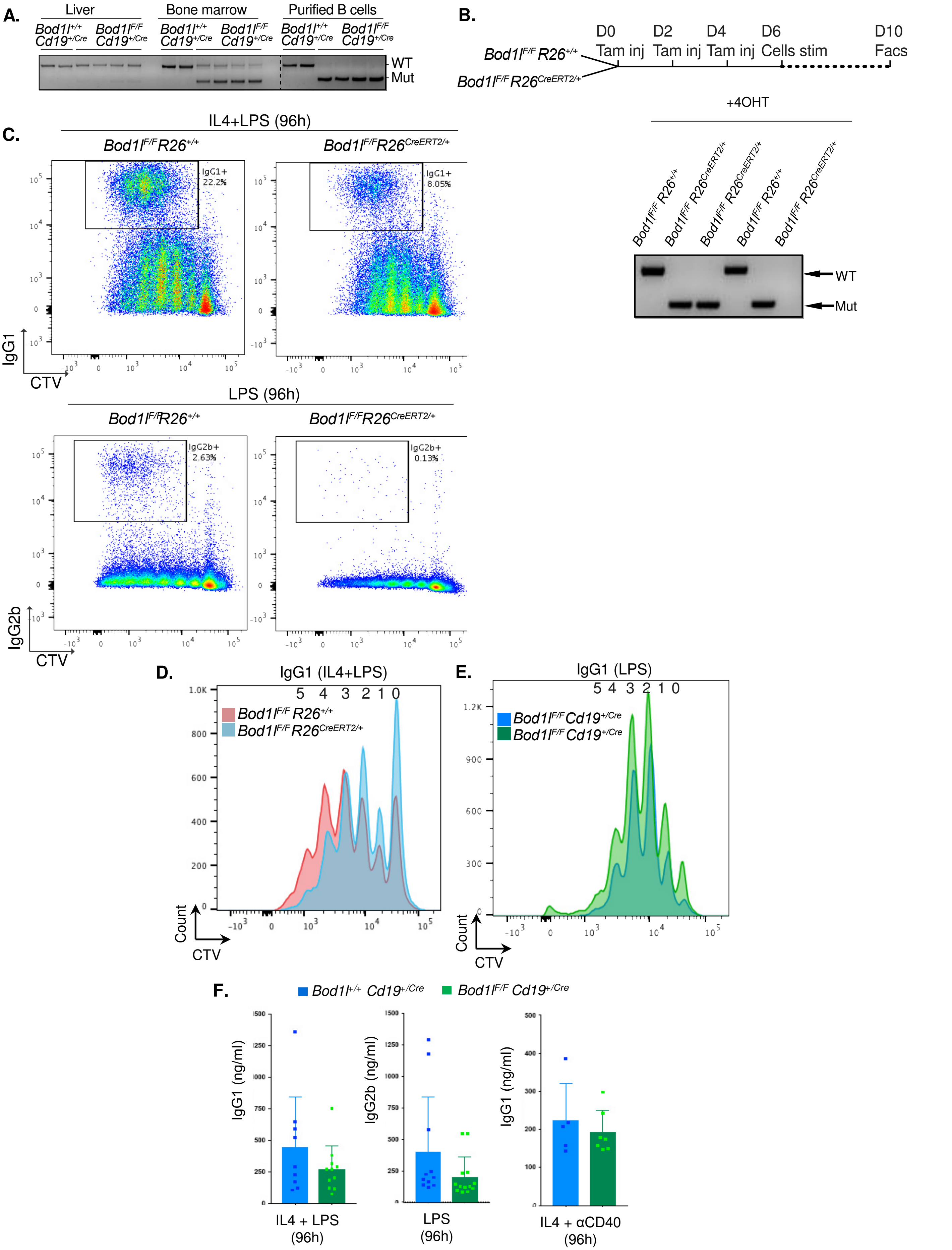

A.
Liver
Bone marrow
Purified B cells
Bod1l+/+ Cd19+/Cre
Bod1lF/F Cd19+/Cre
WT
Mut
B.
Bod1lF/F R26+/+
Bod1lF/F R26CreERT2/+
D0 Tam inj
D2 Tam inj
D4 Tam inj
D6 Cells stim
D10 Facs
+4OHT
WT
Mut
C.
IL4+LPS (96h)
Bod1lF/F R26+/+
Bod1lF/F R26CreERT2/+
IgG1+ 22.2%
IgG1+ 8.05%
IgG1
CTV
LPS (96h)
IgG2b+ 2.63%
IgG2b+ 0.13%
IgG2b
CTV
D.
IgG1 (IL4+LPS)
5 4 3 2 1 0
Count
CTV
E.
IgG1 (LPS)
5 4 3 2 1 0
Bod1lF/F Cd19+/Cre
Bod1lF/F Cd19+/Cre
Count
CTV
F.
Bod1l+/+ Cd19+/Cre
Bod1lF/F Cd19+/Cre
IgG1 (ng/ml)
IgG2b (ng/ml)
IgG1 (ng/ml)
IL4 + LPS (96h)
LPS (96h)
IL4 + αCD40 (96h)

Figure S5, related to Figure 5: Bod1l deletion leads to defective class switch recombination. (A) Cells were isolated from *Bod1l*$^{+/+}$*Cd19*$^{+/Cre}$ or *Bod1l*$^{F/F}$*Cd19*$^{+/Cre}$ mice, and the expression of mutant *Bod1l* alleles was analysed by PCR. **(B)** Schematic of tamoxifen treatments in *Bod1l*$^{F/F}$*R26*$^{+/+}$ or *Bod1l*$^{F/F}$*R26*$^{CreERT2/+}$ mice, and genotyping of *Bod1l* alleles in splenic cells (below). **(C)** Splenic B cells from *Bod1l*$^{F/F}$*R26*$^{+/+}$ or *Bod1l*$^{F/F}$*R26*$^{CreERT2/+}$ mice were stimulated as indicated and stained for surface IgG1 or IgE. **(D-E)** Cell trace violet (CTV) dilutions in purified B cells isolated from the denoted mouse genotype, cultured for 96 h in the presence of LPS and IL-4 and then assessed by FACS. **(F)** CD19+ B cells were isolated from *Bod1l*$^{+/+}$*Cd19*$^{+/Cre}$ or *Bod1l*$^{F/F}$*Cd19*$^{+/Cre}$ mice 96 h after *in vitro* stimulation with the indicated factors, and immunoglobulins quantified by ELISA. Plots in all cases represent mean data from n=3 mice. Error bars = mean ± SEM.

Figure S6:

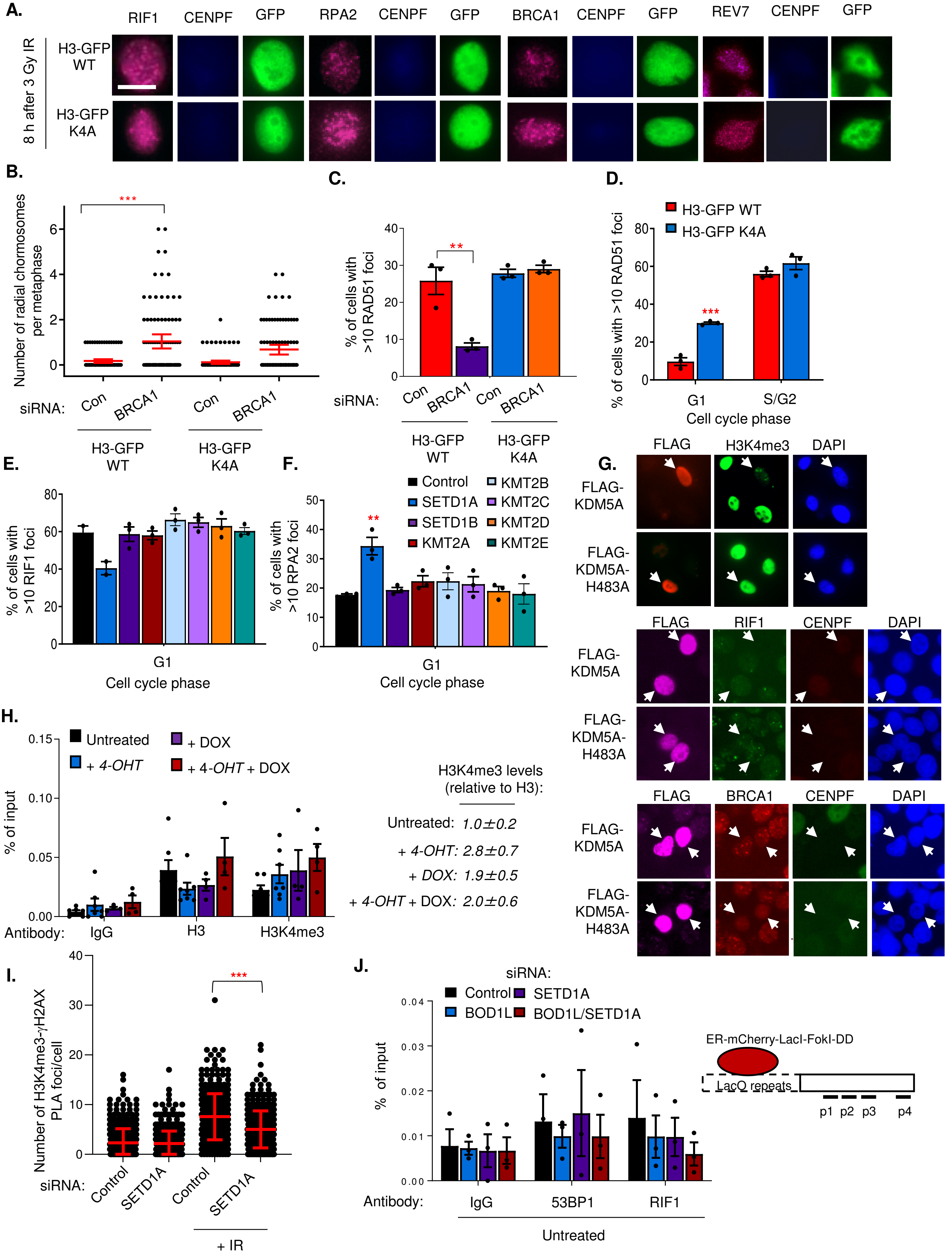
A.
RIF1
CENPF
GFP
RPA2
BRCA1
REV7
8 h after 3 Gy IR
H3-GFP WT
H3-GFP K4A
B.
***
Number of radial chromosomes per metaphase
siRNA:
Con
BRCA1
H3-GFP WT
H3-GFP K4A
C.
**
% of cells with >10 RAD51 foci
D.
H3-GFP WT
H3-GFP K4A
***
% of cells with >10 RAD51 foci
G1
S/G2
Cell cycle phase
E.
% of cells with >10 RIF1 foci
F.
Control
SETD1A
SETD1B
KMT2A
KMT2B
KMT2C
KMT2D
KMT2E
% of cells with >10 RPA2 foci
G.
FLAG
H3K4me3
DAPI
FLAG-KDM5A
FLAG-KDM5A-H483A
CENPF
H.
Untreated
+ 4-OHT
+ DOX
+ 4-OHT + DOX
% of input
Antibody:
IgG
H3
H3K4me3
H3K4me3 levels (relative to H3):
Untreated: 1.0±0.2
+ 4-OHT: 2.8±0.7
+ DOX: 1.9±0.5
+ 4-OHT + DOX: 2.0±0.6
I.
Number of H3K4me3-γH2AX PLA foci/cell
Control
+ IR
J.
siRNA:
BOD1L
BOD1L/SETD1A
53BP1
Untreated
ER-mCherry-LacI-FokI-DD
LacO repeats
p1
p2
p3
p4

Figure S6, related to Figure 6: H3K4 methylation promotes RIF1 recruitment to DNA double-strand breaks and supresses BRCA1-mediated end resection. (A) Representative images of ionizing radiation-induced RIF1, RPA2 and BRCA1 foci in H3-GFP WT and K4A from Figure 6B-G. Scale bars = 10 µm. **(B)** Radial chromosome formation in cells from Figure 6H was analysed by Giemsa staining and light microscopy. **(C)** Cells from Figure 6H were harvested 24 h post olaparib treatment, immunostained with an antibody against RAD51, and foci formation enumerated by fluorescence microscopy. **(D)** H3-GFP WT and K4A cells were exposed to ionizing radiation (IR), immunostained as above, and foci formation enumerated. **(E-F)** HeLa cells were transfected with the indicated siRNAs, exposed to IR, and formation of RIF1 or RPA2 foci monitored as above. **(G)** Representative images of HeLa cells from Figure 6K. Arrows denote transfected cells. **(H)** U-2-OS-FokI cells were treated with 4-OHT and/or doxycycline, chromatin was isolated and ChIP was perfromed with the indicated antibodies and then quantified by qPCR. Data represent the average signal across the four primer pairs represented in the schematic in Figure 6N or Figure S6J. **(I)** Quantification of PLA signals between γH2AX and H3K4me3 in HeLa cells transfected with the indicated siRNAs and exposed to IR. **(J)** U-2-OS-FokI cells were transfected with the indicated siRNAs, undamaged chromatin was isolated and ChIP was performed with the indicated antibodies and quantified by qPCR. Data represent the average signal across the four primer pairs represented in the schematic. In all cases n=3 biologically independent experiments. Error bars indicate mean ± s.e.m. *P* values, unpaired two-tailed *t*-test except (B and J) (Mann Whitney), $^{*}p \leq 0.05$, $^{**}p \leq 0.01$ and $^{***}p \leq 0.001$.

Figure S7:

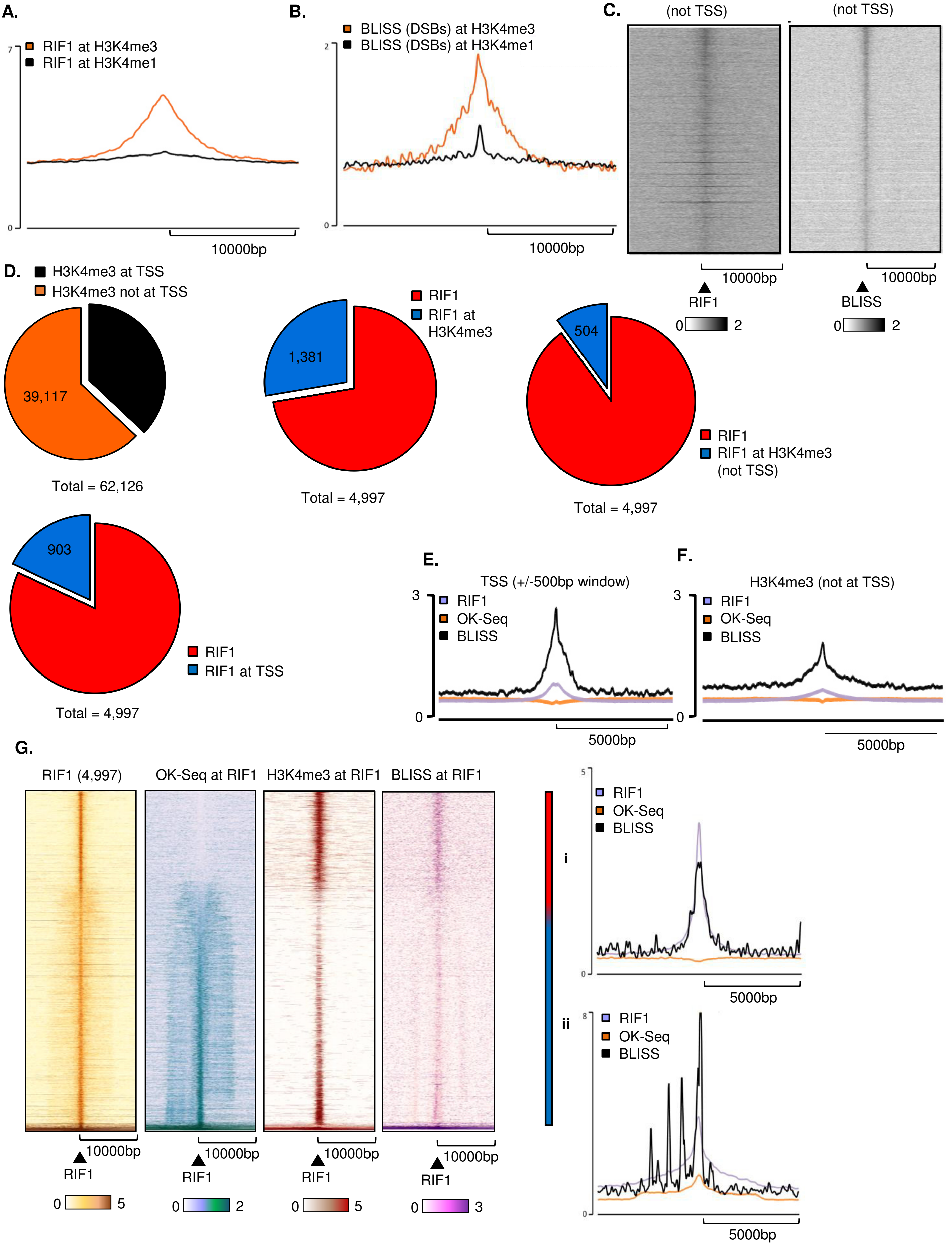

A.
RIF1 at H3K4me3
RIF1 at H3K4me1
7
0
10000bp
B.
BLISS (DSBs) at H3K4me3
BLISS (DSBs) at H3K4me1
2
0
10000bp
C.
RIF1 at H3K4me3 (not TSS)
BLISS (DSBs) at H3K4me3 (not TSS)
10000bp
RIF1
0 2
10000bp
BLISS
0 2
D.
H3K4me3 at TSS
H3K4me3 not at TSS
39,117
Total = 62,126
RIF1
RIF1 at H3K4me3
1,381
Total = 4,997
504
RIF1
RIF1 at H3K4me3 (not TSS)
Total = 4,997
903
RIF1
RIF1 at TSS
Total = 4,997
E.
TSS (+/-500bp window)
3
0
RIF1
OK-Seq
BLISS
5000bp
F.
H3K4me3 (not at TSS)
3
0
RIF1
OK-Seq
BLISS
5000bp
G.
RIF1 (4,997)
OK-Seq at RIF1
H3K4me3 at RIF1
BLISS at RIF1
10000bp
RIF1
0 5
10000bp
RIF1
0 2
10000bp
RIF1
0 5
10000bp
RIF1
0 3
i
5
0
RIF1
OK-Seq
BLISS
5000bp
ii
8
0
RIF1
OK-Seq
BLISS
5000bp

Figure S7, related to Figure 7: RIF1 coincides with sites of endogenous DSBs marked by H3K4me3 in mouse ESCs. **(A-B)** Chromatin immunoprecipitation profiles of murine RIF1 and profiles of BLISS DSB signals at peak sites of H3K4me1 and H3K4me3. **(C)** Genomic profiles of RIF1 chromatin immoprecipitation and BLISS signals over H3K4me3-positive areas that lie outside areas defined by ENCODE as TSS. **(D)** Overlap of chromatin immunoprecipitation peaks sites of murine RIF1, H3K4me3 or ENCODE TSS. Total number of peaks is denoted below. **(E-F)** Chromatin immunoprecipitation profiles of murine RIF1, profiles of BLISS DSB signals and Okazaki fragment sequencing (OK-seq) profiles at ENCODE TSS (E) or peak sites of H3K4me3 (F). **(G)** Genomic profiles of BLISS signals, H3K4me3 chromatin immunoprecipitation profiles and OK-seq profiles over RIF1 peak sites. Profiles are then subdivided into 2 groups with profiles of Rif, BLISS and OK-seq signals denoted: (i) areas with no OK-seq signal and strong BLISS and H3K4me3 signals; (ii) areas with a strong OK-seq signal and variable BLISS and H3K4me3 signals. Data is from Yan *et al.,* 2017 and Petruk *et al.,* 2018.

Table S1:

Figure	Number and genotype of animals
Fig 5A	6 *Bod1l*$^{+/+}$ *Cd19*$^{+/Cre}$ (3♀; 3♂) and 6 *Bod1l*$^{F/F}$ *Cd19*$^{+/Cre}$ (4♀; 2♂)
Fig B-C	2 *Bod1l*$^{F/F}$ *R26*$^{+/+}$ (1♀; 1♂) and 3 *Bod1l*$^{F/F}$ *R26*$^{CreERT2/+}$ (4♀; 1♂)
Fig 5D and S5E-F	6 *Bod1l*$^{+/+}$ *Cd19*$^{+/Cre}$ (3♀; 3♂) and 8 *Bod1l*$^{F/F}$ *Cd19*$^{+/Cre}$ (5♀; 3♂)
Fig 5E-F	5 *Bod1l*$^{+/+}$ *Cd19*$^{+/Cre}$ (3♀; 2♂) and 8 *Bod1l*$^{F/F}$ *Cd19*$^{+/Cre}$ (3♀; 5♂)

Table S1, related to STAR Methods: Details of the animals used in the study. The individual numbers, genotype and gender of the animals used for this study are detailed. ♂ denotes male, ♀ denotes female.
